# The tyrosine transporter of *Toxoplasma gondii* is a member of the newly defined apicomplexan amino acid transporter (ApiAT) family

**DOI:** 10.1371/journal.ppat.1007577

**Published:** 2019-02-11

**Authors:** Kathryn E. R. Parker, Stephen J. Fairweather, Esther Rajendran, Martin Blume, Malcolm J. McConville, Stefan Bröer, Kiaran Kirk, Giel G. van Dooren

**Affiliations:** 1 Research School of Biology, Australian National University, Canberra, ACT, Australia; 2 Department of Biochemistry and Molecular Biology and the Bio21 Institute of Molecular Science and Biotechnology, University of Melbourne, Parkville, VIC, Australia; 3 Robert Koch Institute, Berlin, Germany; University of Geneva, SWITZERLAND

## Abstract

Apicomplexan parasites are auxotrophic for a range of amino acids which must be salvaged from their host cells, either through direct uptake or degradation of host proteins. Here, we describe a family of plasma membrane-localized amino acid transporters, termed the Apicomplexan Amino acid Transporters (ApiATs), that are ubiquitous in apicomplexan parasites. Functional characterization of the ApiATs of *Toxoplasma gondii* indicate that several of these transporters are important for intracellular growth of the tachyzoite stage of the parasite, which is responsible for acute infections. We demonstrate that the ApiAT protein *Tg*ApiAT5-3 is an exchanger for aromatic and large neutral amino acids, with particular importance for L-tyrosine scavenging and amino acid homeostasis, and that *Tg*ApiAT5-3 is critical for parasite virulence. Our data indicate that *T*. *gondii* expresses additional proteins involved in the uptake of aromatic amino acids, and we present a model for the uptake and homeostasis of these amino acids. Our findings identify a family of amino acid transporters in apicomplexans, and highlight the importance of amino acid scavenging for the biology of this important phylum of intracellular parasites.

## Author summary

The Apicomplexa comprise a large number of parasitic protozoa that have obligate intracellular lifestyles and cause significant human and animal diseases, including malaria, cryptosporidiosis, toxoplasmosis, coccidiosis in poultry, and various cattle fevers. Apicomplexans must scavenge essential nutrients from their hosts in order to proliferate and cause disease, including a range of amino acids. The direct uptake of these nutrients is presumed to be mediated by transporter proteins located in the plasma membrane of intracellular stages, although the identities of these proteins are poorly defined. Using a combination of bioinformatic, genetic, cell biological, and physiological approaches, we have characterized an apicomplexan-specific family of plasma membrane-localized transporter proteins that we have called the Apicomplexan Amino acid Transporters (ApiATs). We show that *Tg*ApiAT5-3, a member of the family in the apicomplexan *Toxoplasma gondii*, is an exchanger for aromatic and large neutral amino acids. In particular, it is critical for uptake of tyrosine, and for parasite virulence in a mouse infection model. We conclude that ApiATs are a family of plasma membrane transporters that play crucial roles in amino acid scavenging by apicomplexan parasites.

## Introduction

Apicomplexans are intracellular parasites that cause a range of diseases in humans and animals, imposing a major health and economic burden on many countries. In humans, *Plasmodium* species are the causative agents of malaria [1], while *Cryptosporidium* is a major cause of diarrheal disease and death in children in the developing world [2]. *Toxoplasma gondii* can infect virtually all nucleated cells in warm-blooded animals, and is thought to chronically infect one-third of the world’s human population. *T*. *gondii* infections are usually asymptomatic, but infection in immunocompromised patients may lead to life-threatening toxoplasmic encephalitis, and congenital toxoplasmosis may result in severe birth defects or death of the developing fetus [3].

A common feature of parasites is that they rely on their hosts to supply them with the nutrients necessary for their growth and replication, such as sugars, amino acids, nucleosides, and vitamins [4–6]. Transporters are integral membrane proteins that facilitate the transfer of substrates across biological membranes. In apicomplexans, transporters provide the major route for the acquisition of nutrients and the removal of waste products across the plasma membrane [5, 7], and these proteins are important for parasite survival and virulence [8, 9]. Despite this, the transporters responsible for the uptake of many essential nutrients in apicomplexans have not been defined.

A family of Novel Putative Transporters (the NPT family) was initially identified in *Plasmodium falciparum* using a bioinformatics approach [10]. The five *P*. *falciparum* NPT family proteins were predicted to be polytopic membrane proteins with a secondary structure characteristic of solute transporters, although they have limited sequence similarity to other eukaryotic or prokaryotic transporters. The NPT family protein *Pb*NPT1 localizes to the plasma membrane of the mouse malaria-causing parasite *P*. *berghei*, and is essential for gametocyte development in the murine host and subsequent mosquito transmission [8, 9, 11]. Other *P*. *berghei* NPT family proteins, *Pb*MFR4 and *Pb*MFR5, are essential for progression through the insect stages of the life cycle, while *Pb*MFR2 and *Pb*MFR3 are important for exflagellation of male gametes and sporozoite formation, respectively, but are not essential for completion of the *P*. *berghei* life cycle [8]. A saturation mutagenesis screen revealed that all five NPTs in *P*. *falciparum* are dispensable for the growth of asexual blood stages of the parasite under *in vitro* culture conditions [12]. In *T*. *gondii*, *Tg*NPT1 localizes to the plasma membrane and is essential for parasite growth and virulence [9]. Both *Pb*NPT1 and *Tg*NPT1 are cationic amino acid transporters, with *Pb*NPT1 functioning as a general cationic amino acid transporter and *Tg*NPT1 functioning as a selective arginine transporter [9]. The functions of other NPT-family proteins are not known, although one member of the family has been associated with susceptibility to the anti-*T*. *gondii* drug sinefungin [13].

In this study, we have demonstrated that the NPTs are phylogenetically related, and broadly distributed within the apicomplexan phylum. We have characterized the NPT family proteins in *T*. *gondii*, demonstrating that 10 of the sixteen members of the family are expressed in the disease-causing tachyzoite stage of the parasite, and that the majority of these localize to the parasite periphery. We have demonstrated that at least three of these proteins are important for *in vitro* growth of the parasite. Using a combination of genetic, physiological and heterologous expression approaches, we have shown that one of the previously uncharacterized *T*. *gondii* NPT-family members transports aromatic and large neutral amino acids, and that this transporter is particularly important for the uptake of tyrosine into the parasite. We conclude that NPTs are a family of amino acid transporter proteins found in apicomplexans, and we propose that the family be renamed the Apicomplexan Amino acid Transporter (ApiAT) family.

## Results

### ApiATs are broadly-distributed in apicomplexan parasites

To identify ApiAT-family proteins in the apicomplexan parasites *T*. *gondii*, *Neospora caninum*, *Eimeria tenella*, *P*. *falciparum*, *P*. *berghei*, *Theileria annulata*, *Babesia bovis* and *Cryptosporidium parvum*, we undertook Basic Local Alignment Search Tool (BLAST) searches using each of the five *P*. *falciparum* ApiATs as initial query sequences (www.eupathdb.org; [14]) We also undertook BLAST searches of the genomes from the chromerids *Chromera velia* and *Vitrella brassicaformis*, which are close free-living relatives of apicomplexans [15] (www.eupathdb.org, www.blast.ncbi.nlm.nih.gov; [14, 16]). In addition to the previously described five *Plasmodium* ApiAT family proteins, we identified sixteen ApiAT family proteins in both *T*. *gondii* and *N*. *caninum*, eight in *E*. *tenella*, nine in *T*. *annulata*, six in *B*. *bovis*, and one in *C*. *parvum* (S1 Fig; S1 Table). Using this search strategy, we were unable to identify ApiAT family proteins in chromerids. Using *Pb*NPT1 as a search query in profile hidden Markov Model searches, we identified the LAT3 and LAT4 proteins from humans (http://hmmer.org; [17]; S2 Fig). LAT3 and LAT4 are members of the SLC43 family of the major facilitator superfamily of transporters, and mediate the transport of branched chain and other large neutral amino acids [18].

To determine the phylogenetic relationships between ApiAT family proteins, we constructed a multiple sequence alignment (S1 Fig). This revealed the presence of a major facilitator superfamily (MFS) signature sequence between transmembrane domains 2 and 3 of most ApiAT family protein (S2 Fig; [19, 20]). Most ApiAT proteins were predicted to be polytopic membrane proteins containing 12 transmembrane domains (www.cbs.dtu.dk/services/TMHMM/; [21]), and exhibited highest sequence similarity in the regions encompassing these transmembrane domains (S1 Fig). These analyses are consistent with previous studies and protein database annotations placing members of the ApiAT family into the major facilitator superfamily of transporters [9, 10].

**Fig 1:**
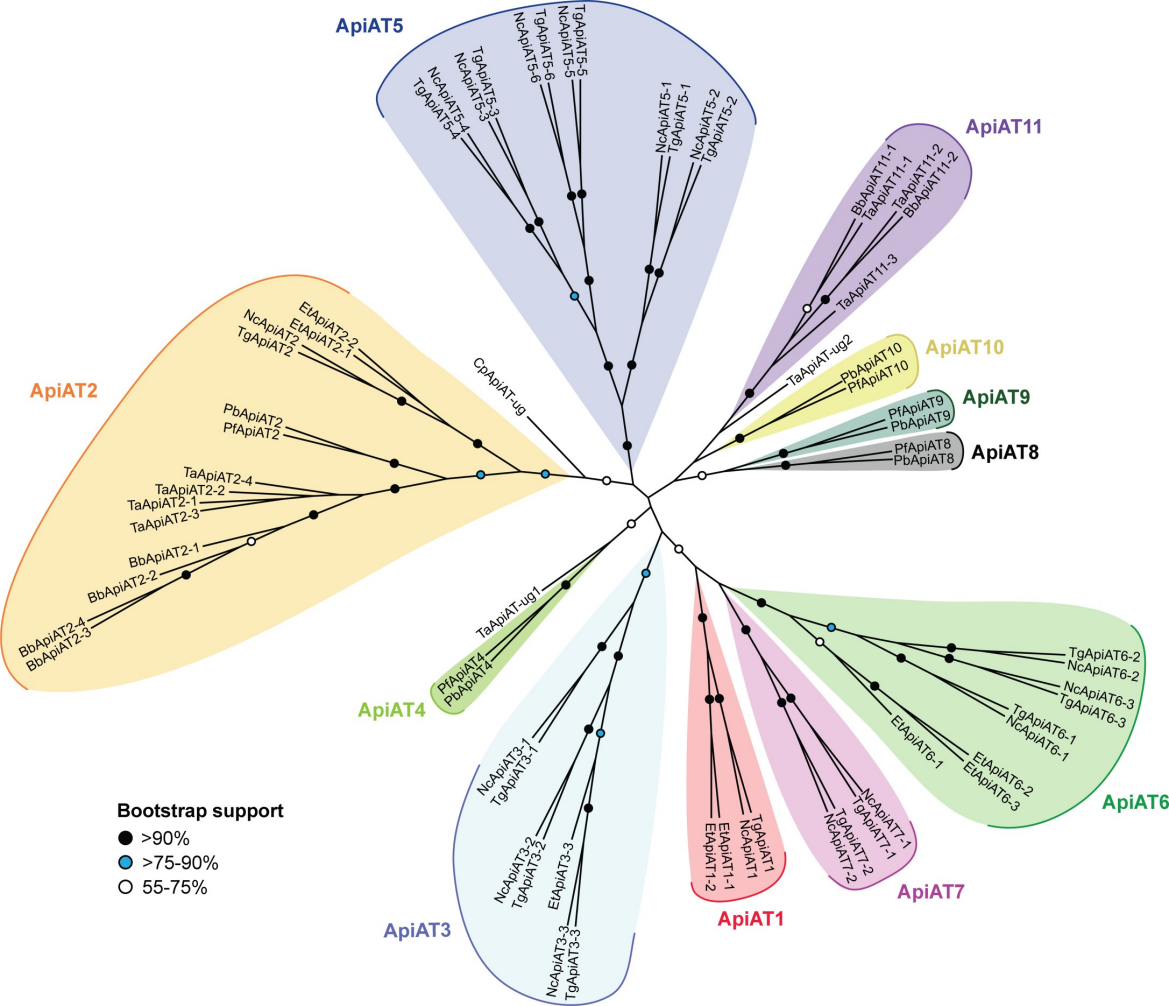
Phylogenetic analysis of ApiAT family proteins. Consensus maximum likelihood tree of ApiAT family proteins. The tree was generated from a multiple sequence alignment of 66 putative ApiAT proteins from a range of apicomplexans, with 464 residues used in the analysis. Bootstrap values are depicted by black circles (>90% support), blue circles (>75–90% support), or white circles (55–75% support). The tree is unrooted. Abbreviations: Bb, *Babesia bovis*; Cp, *Cryptosporidium parvum*; Et, *Eimeria tenella*; Nc, *Neospora caninum*; Pb. *Plasmodium berghei*; Pf, *Plasmodium falciparum*; Tg, *Toxoplasma gondii*; Ta, *Theileria annulate*; ug, ungrouped.

We next performed a maximum likelihood phylogenetic analysis. This revealed the presence of multiple ApiAT subfamilies, which we defined as groupings that had greater than 75% bootstrap support (Fig 1). Orthologs of the ApiAT2 subfamily were present in all organisms in the study with the exception of *Cryptosporidium parvum*, although the only *C*. *parvum* ApiAT protein that we identified grouped with the ApiAT2 subfamily with weak support (Fig 1). Members of the ApiAT1, ApiAT3, ApiAT5, ApiAT6 and ApiAT7 subfamilies were restricted to coccidians (a group of apicomplexans that includes *T*. *gondii* and *N*. *caninum*), the ApiAT4, ApiAT8, ApiAT9 and ApiAT10 subfamilies were restricted to the *Plasmodium* genus, and the ApiAT11 family was restricted to the piroplasms *T*. *annulata* and *B*. *bovis* (Fig 1). The ApiAT8 family includes the previously described cationic transporter *Pb*NPT1 [9], here annotated as *Pb*ApiAT8. Although similar in function to the *T*. *gondii* arginine transporter *Tg*ApiAT1 (previously *Tg*NPT1), *Pb*ApiAT8 and *Tg*ApiAT1 appear not to be orthologous.

### *T*. *gondii* ApiATs localize to the parasite periphery

Previous studies demonstrated that the *P*. *berghei* ApiAT8 protein (previously *Pb*NPT1) localized to the periphery of the parasite (likely to the plasma membrane), and that the *T*. *gondii* ApiAT1 protein (previously *Tg*NPT1) localized to the plasma membrane [9, 11]. To determine the expression pattern and localization of ApiAT family proteins in *T*. *gondii*, we introduced a hemagglutinin (HA) tag into the 3’ end of the open reading frame of the remaining fifteen ApiAT genes (S3A and S3B Fig).

**Fig 2:**
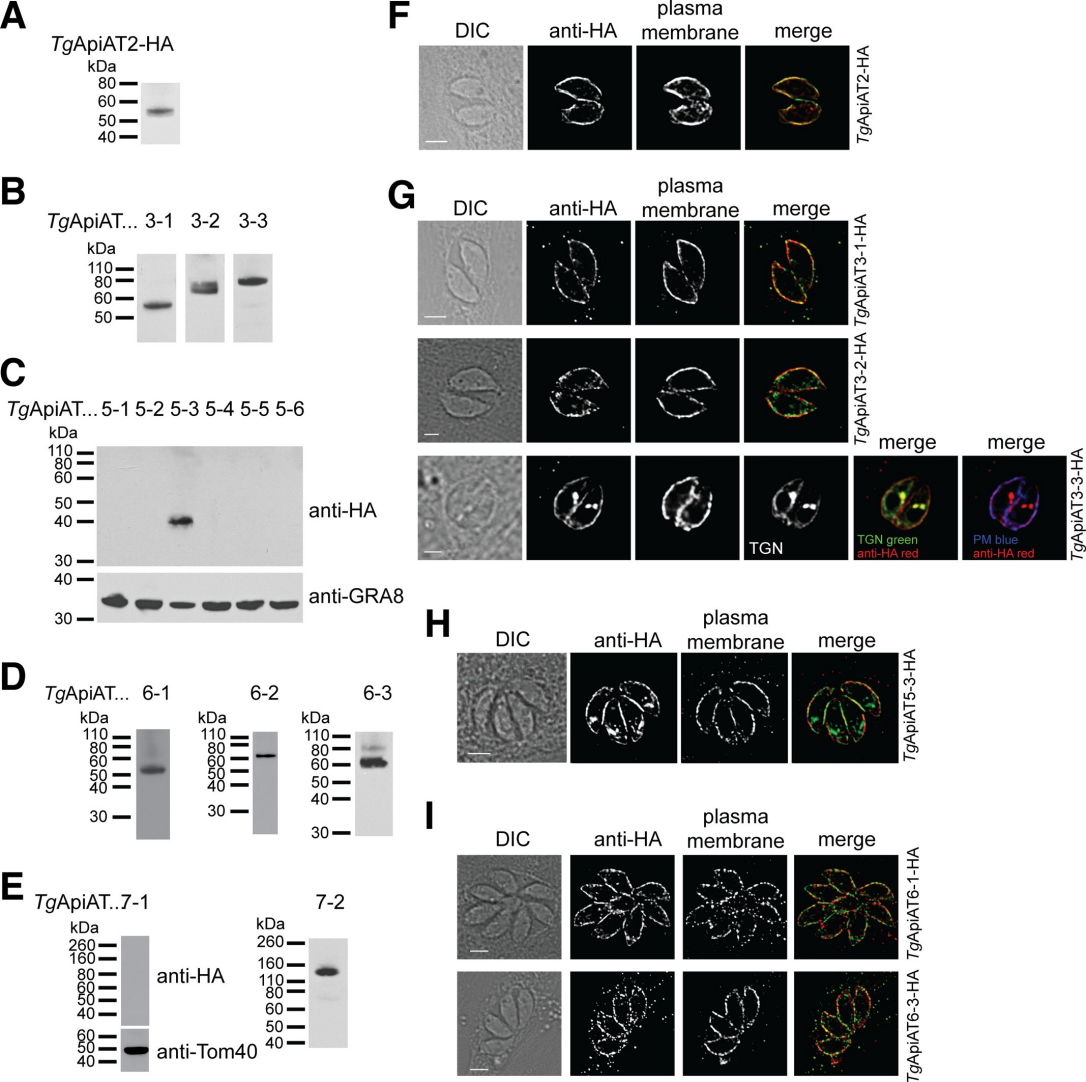
Expression and localization analysis of *T*. *gondii* ApiAT family proteins. (A-E) Western blots with anti-HA antibodies to measure the expression and molecular mass of tagged *Tg*ApiAT proteins in tachyzoites stages of the parasite. Western blots with antibodies against GRA8 and Tom40 were used to test for the presence of protein in samples where the HA-tagged *Tg*ApiAT protein was not detected. (F-I) Immunofluorescence assays with anti-HA antibodies to determine the localisation of HA-tagged *Tg*ApiAT proteins (green in merge). Samples were co-labelled with antibodies against the plasma membrane marker P30 (red in merge). *Tg*ApiAT3-3-HA-expressing parasites were co-transfected with the trans-Golgi network (TGN) marker Stx6-GFP [63], and labelled with anti-HA (red in merge), anti-P30 (plasma membrane, PM; blue in merge) and anti-GFP (green in merge) antibodies. All scale bars are 2 μm.

Western blotting indicated that *Tg*ApiAT2, *Tg*ApiAT3-1, *Tg*ApiAT3-2, *Tg*ApiAT3-3, *Tg*ApiAT5-3, *Tg*ApiAT6-1, *Tg*ApiAT6-2, *Tg*ApiAT6-3, and *Tg*ApiAT7-2 proteins were expressed in tachyzoite stage parasites (Fig 2A–2E). We were unable to detect expression of *Tg*ApiAT5-1, *Tg*ApiAT5-2, *Tg*ApiAT5-4, *Tg*ApiAT5-5, *Tg*ApiAT5-6 and *Tg*ApiAT7-1. Of the ApiATs that were expressed in *T*. *gondii* tachyzoites, most exhibited a lower mass than predicted (S1 Table). Although we cannot rule out the possibility of N-terminal processing in these proteins, we note that hydrophobic membrane proteins commonly run faster than predicted on sodium dodecyl sulfate-polyacrylamide gel electrophoresis (SDS-PAGE; [22]). Immunofluorescence assays (IFAs) demonstrated that *Tg*ApiAT2, *Tg*ApiAT3-1, *Tg*ApiAT3-2, *Tg*ApiAT3-3, *Tg*ApiAT5-3, *Tg*ApiAT6-1 (as reported previously; [23]), and *Tg*ApiAT6-3 localized to parasite periphery, overlapping with the plasma membrane marker P30 (Fig 2F–2I). *Tg*ApiAT3-3 showed additional localization to the trans-Golgi network (Fig 2G). Although detectable by western blotting (Fig 2D and 2E), we could not detect *Tg*ApiAT6-2 or *Tg*ApiAT7-2 by IFA, possibly because the level of expression of these proteins was below the detection limits of IFAs.

*T*. *gondii*, like many apicomplexans, contains a series of flattened membrane sacs termed alveolae that contribute to the inner membrane complex (IMC) that localizes just beneath the plasma membrane of these parasites [24]. The IMC first appears as the ‘buds’ of daughter parasites within the parent cell just before cytokinesis. To compare the localization of an ApiAT protein with the IMC, we co-labelled *Tg*ApiAT5-3-HA parasites with anti-HA and anti-IMC antibodies. We found that *Tg*ApiAT5-3-HA did not localize to daughter buds (S4A Fig). *Tg*ApiAT5-3-HA localization extended along the cell periphery at the apical and basal ends of the parasite, regions from which the IMC marker was absent (S4A and S4B Fig, arrows). Taken together, these data indicate that *Tg*ApiAT5-3 is localized primarily to the plasma membrane, although in the absence of higher resolution microscopy approaches, we cannot rule out that some could also be located in IMC.

We conclude that ten of the sixteen *Tg*ApiAT proteins are expressed in the tachyzoite stage of *T*. *gondii*, and that those with detectable expression by IFA all localize to the periphery of the parasite, most likely to the parasite plasma membrane.

### CRISPR/Cas9-mediated disruption of ApiAT-encoding genes identifies three family members that are important for parasite growth

To determine the importance of *Tg*ApiATs for parasite growth, we attempted to genetically disrupt all sixteen *T*. *gondii* ApiATs using a CRISPR/Cas9-based approach [25]. With this strategy, we were able to disrupt fifteen of the sixteen *Tg*ApiAT genes (S2 Table). The mutant parasites all had frameshift mutations that will lead to the production of truncated (and likely non-functional) proteins. We refer to each of these mutants on the basis of which amino acids will be absent from the final protein (e.g. *Tg*ApiAT5-3 encodes a protein of 504 amino acids; the *Tg*ApiAT5-3 mutant is truncated from residue 188 and is referred to as *apiAT5-3*^*Δ188–504*^). *Tg*ApiAT1 could only be disrupted when parasites were grown in excess arginine, as described previously [9]. We were unable to generate frameshift mutations in *Tg*ApiAT6-1 after screening 12 clones from three separate transfections of a guide RNA targeting the *Tg*ApiAT6-1 locus. Of these clones, two had a 3 bp insertion and one had a 3 bp deletion, indicating that the guide RNA was capable of targeting the *Tg*ApiAT6-1 locus (S2 Table).

**Fig 3:**
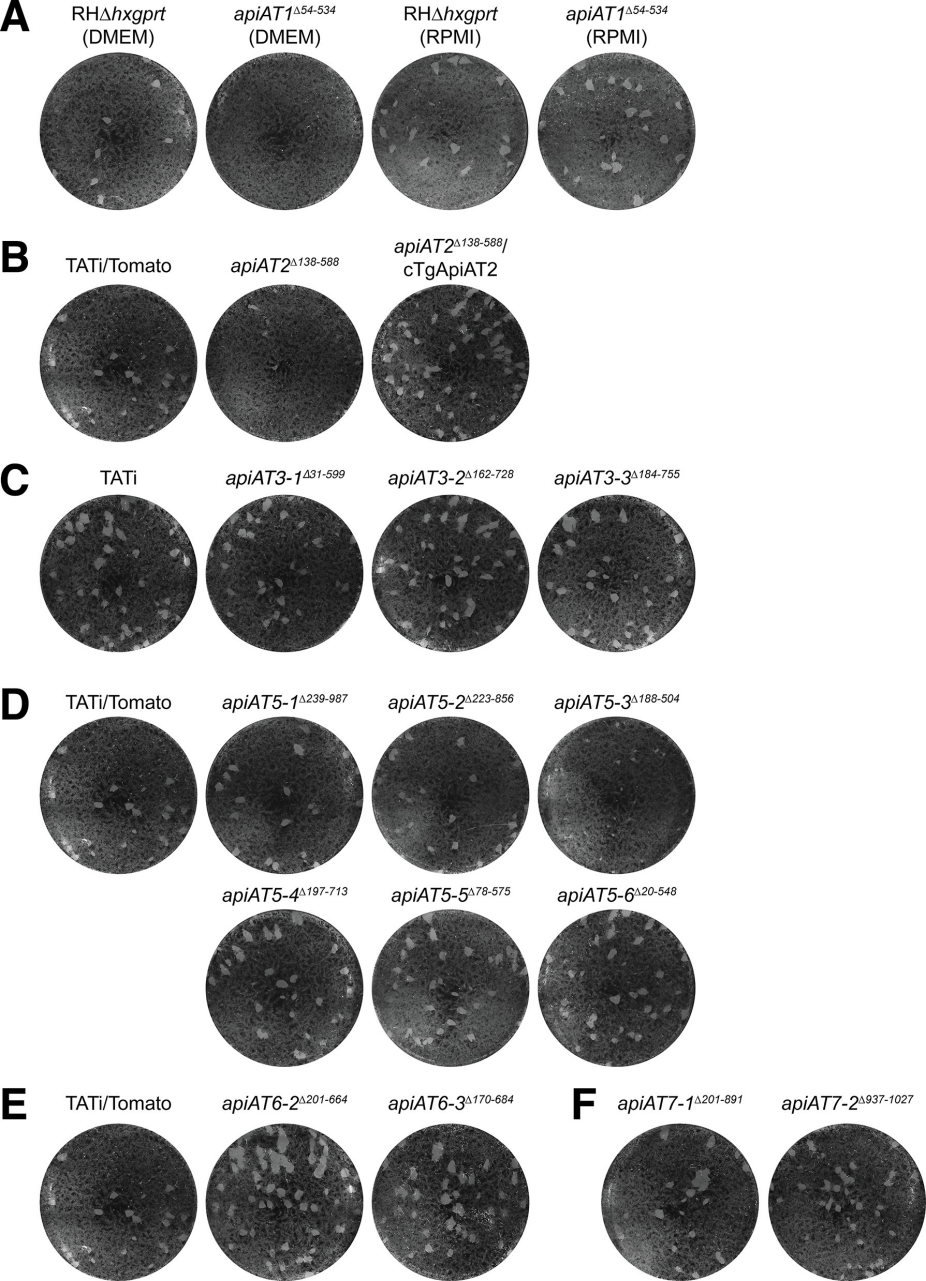
Genetic disruption of *T*. *gondii* ApiAT family proteins reveals the importance of *Tg*ApiAT2 and *Tg*ApiAT5-3 for parasite growth *in vitro*. (A-F) Plaque assays depicting growth of disrupted *Tg*ApiAT strains and their corresponding parental WT strain. 150 parasites were added to wells of a 6-well plate and cultured for 9 days in DMEM. (A) WT (RHΔ*hxpgrt*) and *apiAT1*^*Δ54–534*^ parasites grown in DMEM (left) or RPMI (right). (B) WT (TATi/Tomato), *apiAT2*^*Δ138–588*^ and *apiAT2*^*Δ138–588*^ parasites complemented with a constitutively expressed *Tg*ApiAT2 (*apiAT2*^*Δ138-588*^/c*Tg*ApiAT2). (C) WT (TATi) and *apiAT3* sub-family mutants. (D) WT (TATi/Tomato) and *apiAT5* sub-family mutants. (E) WT (TATi/Tomato) and *apiAT6* sub-family mutants. (F) *apiAT7* sub-family mutants. Note that the TATi/Tomato strain served as WT strain for the *apiAT2*, *apiAT5*, *apiAT6*, and *apiAT7* sub-family mutants, and the identical image of the TATi/Tomato plaque assay is shown in B, D and E to facilitate interpretation of the data. All images are from the same experiment, and are representative of three independent experiments.

To determine which *Tg*ApiATs were important for parasite growth, we performed plaque assays on each of the genetically disrupted *Tg*ApiAT lines grown in human foreskin fibroblasts (HFFs) and cultured in Dulbecco’s modified Eagle’s medium (DMEM). Compared to parental wild type (WT) controls, we observed greatly reduced plaque sizes in the *apiAT2*^*Δ138–588*^ and *apiAT5-3*^*Δ188–504*^ strains (Fig 3). No plaques were observed in the *apiAT1*^*Δ54–534*^ strain grown in DMEM (containing 400 μM L-arginine) but normal growth of this strain was observed when grown in Roswell Park Memorial Institute 1640 (RPMI) medium (containing 1.15 mM L-Arg; Fig 3A), mirroring previous results with *Tg*ApiAT1 knockouts [9]. By contrast, the remaining 12 mutant *apiAT* lines exhibited plaques that were similar in size to those observed in WT controls (Fig 3).

Our previous data indicated that some ApiAT family proteins transport amino acids [9]. We tested each of the mutant *apiAT* strains for whether growth defects were altered in medium containing reduced amino acid concentrations (Minimal Amino Acid Medium, MAAM). The growth defects in the *apiAT2*^*Δ138–588*^ and *apiAT5-3*^*Δ188–504*^ strains were exacerbated in MAAM (S5 Fig), implicating these ApiATs in the uptake of amino acids. By contrast, the *apiAT1*^*Δ54–534*^ strain grew better in MAAM than in DMEM (S5 Fig). Our previous study of the arginine transporter *Tg*ApiAT1 found that parasites lacking this transporter could grow in medium containing a high Arg:Lys ratio [9]. In this context, we note that MAAM contains proportionally more L-Arg than L-Lys (3:1 Arg:Lys; S3 Table) than DMEM (1:2 Arg:Lys). We observed no major growth differences between minimal amino acid and complete growth media for any of the other mutants (S5 Fig).

To test whether the growth defect in the *api*AT2^*Δ138–588*^ strain was due specifically to disruption of the *Tg*ApiAT2 locus, we complemented *api*AT2^*Δ138–588*^ with constitutively expressed *Tg*ApiAT2. This restored parasite growth (Fig 3B).

Together, these results indicate that *Tg*ApiAT1, *Tg*ApiAT2 and *Tg*ApiAT5-3 are required for normal intracellular growth of *T*. *gondii* in standard *in vitro* culture conditions.

### *Tg*ApiAT5-3 is important for amino acid homeostasis in *T*. *gondii*

**Fig 4:**
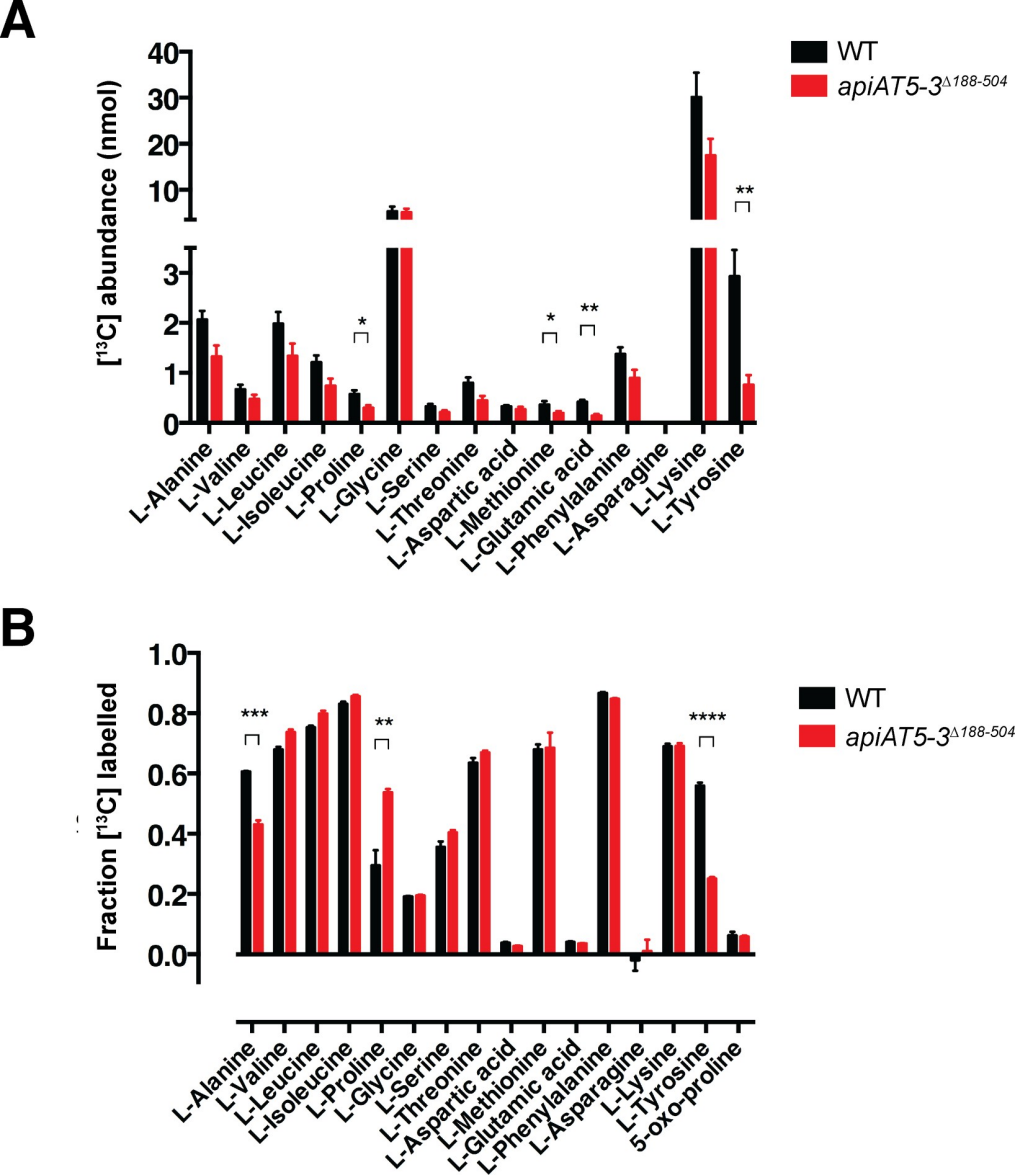
Analysis of [^13^C] amino acid uptake into WT and *apiAT5-3*^*Δ188–504*^ parasites reveals a role for *Tg*ApiAT5-3 in amino acid homeostasis. (A-B) Extracellular WT (TATi/Tomato) or *apiAT5-3*^*Δ188–504*^ tachyzoites were incubated in medium containing [^13^C]-L-amino acids for 15 min. Polar metabolites were extracted and amino acid abundance (A) and levels of [^13^C]-amino acid enrichment (B) in WT (black) and *apiAT5-3*^*Δ188–504*^ (red) tachyzoites determined by GC-MS. Only L-amino acids that could be detected in all experiments are shown. The data are averaged from three independent experiments and error bars represent ± s.e.m. (*, P < 0.05; **, P < 0.01; ***, P < 0.001, ****, P < 0.0001; Student’s *t* test. Where significance values are not shown, the differences were not significant; P > 0.05).

*Tg*ApiAT proteins that are important for parasite growth are likely to have critical roles in nutrient acquisition. In the remainder of this manuscript, we focus on one such protein, *Tg*ApiAT5-3. Our previous study of *Tg*ApiAT1 and *Pb*ApiAT8-1 indicated a key role for these transporters in cationic amino acid uptake [9], and we hypothesized that *Tg*ApiAT5-3 could also function as an amino acid transporter. To investigate this possibility, we incubated WT and *apiAT5-3*^*Δ188–504*^ parasites in medium containing a mixture of [^13^C]-labelled amino acids for 15 mins. Polar metabolites were extracted from parasite lysates and analyzed by GC-MS. These analyses were used to quantitate the levels of intracellular amino acids and determine the extent of labeling with exogenous amino acids based on [^13^C]-enrichment in each of the two strains. Strikingly, both the abundance and fractional labelling of [^13^C]-L-tyrosine (L-Tyr) was reduced significantly in the *apiAT5-3*^*Δ188–504*^ strain (Fig 4). Abundance and fractional labeling of a number of other [^13^C]-amino acids were altered significantly in the *apiAT5-3*^*Δ188–504*^ strain, although none to the same extent as L-Tyr (Fig 4). The levels of several other amino acids were reduced in the *apiAT5-3*^*Δ188–504*^ mutant, although the differences were not statistically significant (Fig 4A), possibly reflecting differences in the growth rate and physiological state of the two lines. In support of this notion, the rate of turnover of these amino acids, as indicated by rate of ^13^C-enrichment in intracellular pools was unchanged (Fig 4B). These data indicate that *Tg*ApiAT5-3 is important for amino acid homeostasis, playing a key role in the uptake of L-Tyr into the parasite.

### *Tg*ApiAT5-3 is an aromatic and large neutral amino acid uniporter with exchange activity

**Fig 5:**
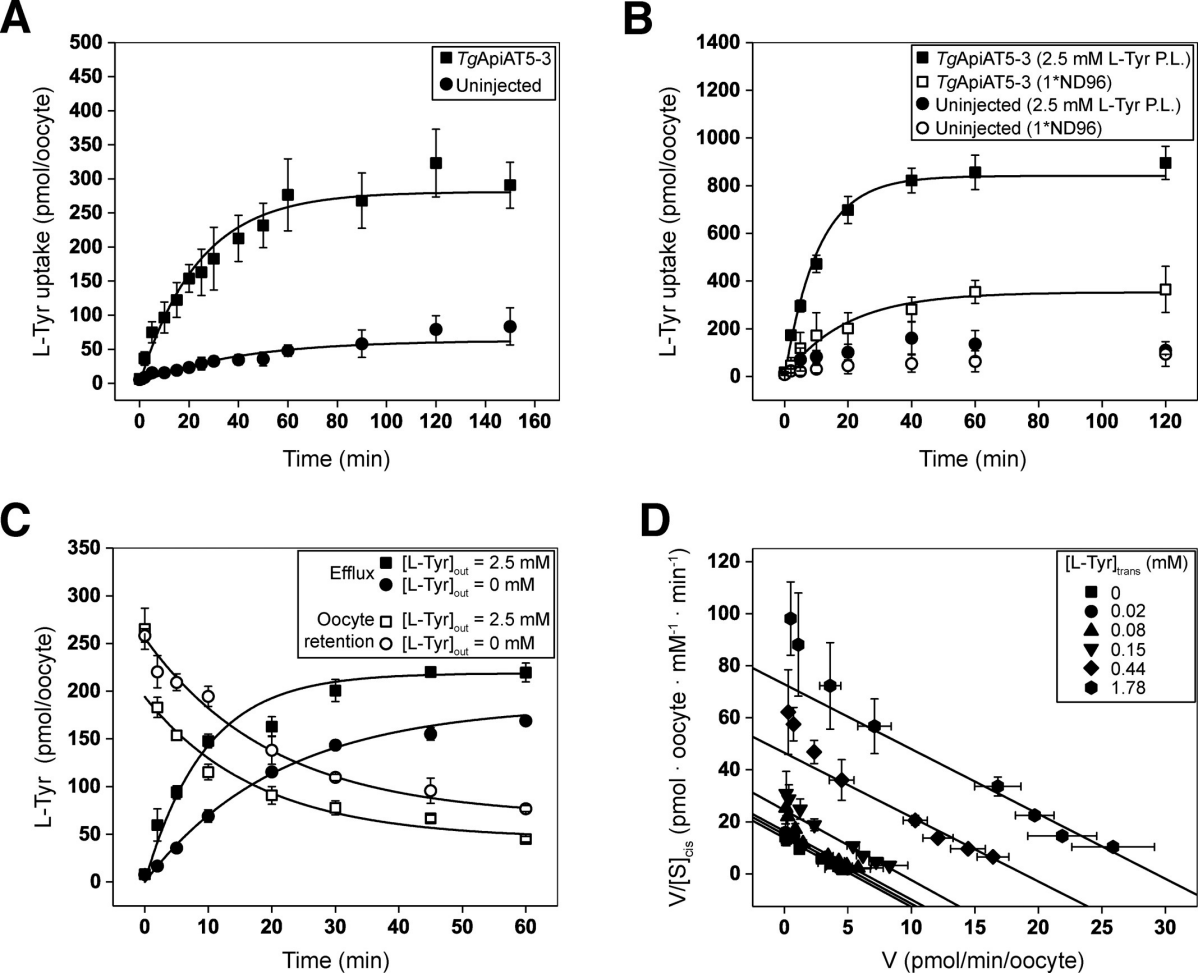
*Tg*ApiAT5-3 is an L-tyrosine transporter that is stimulated by the presence of L-tyrosine on the *trans* side of the membrane. (A) Timecourse for the uptake of L-Tyr into *X*. *laevis* oocytes expressing *Tg*ApiAT5-3 (squares) or into uninjected oocytes (circles). Uptake was measured in the presence of 1 mM L-Tyr containing 0.5 μCi/ml [^14^C]Tyr. Each data point represents the mean uptake in 10 oocytes from a single experiment ± standard deviation, and the data are representative of 3 independent experiments. A first order rate equation was fitted to each timecourse (R^2^ = 0.97 for *Tg*ApiAT5-3-expressing oocytes and R^2^ = 0.77 for uninjected controls). Both the rate constant for L-Tyr uptake and the maximal L-Tyr uptake measured in *Tg*ApiAT5-3-expressing oocytes were significantly higher than those measured in uninjected oocytes (P < 0.01, Student’s *t* tests). (B) *Tg*ApiAT5-3-expressing oocytes (squares) and uninjected oocytes (circles) were preloaded (P.L.) with L-Tyr by incubation in 2.5 mM unlabelled L-Tyr (filled symbols) for 32 or 72 hr, respectively, or not preloaded (open symbols). Following the preincubation period, uptake of L-Tyr was measured in a solution of 1 mM L-Tyr containing 0.5 μCi/ml [^14^C]Tyr. Data show the mean uptake in 10 oocytes from a single experiment ± standard deviation, and are representative of 3 independent experiments. First order rate equations were fitted to the uptake timecourses for the preloaded and non-preloaded *Tg*ApiAT5-3-injected oocytes (R^2^ = 0.98 for preloaded, and R^2^ = 0.95 for non-preloaded oocytes). Both the first order rate constants for L-Tyr uptake and the maximal L-Tyr uptake were significantly higher in preloaded compared to non-preloaded *Tg*ApiAT5-3-expressing oocytes (P < 0.01, Student’s *t* tests). (C) *Tg*ApiAT5-3-expressing oocytes were preloaded by incubation in 1 mM L-Tyr containing 0.5 μCi/ml [^14^C]Tyr for 32 hr. Subsequent efflux (filled symbols) and retention (open symbols) of the preloaded substrate was measured over the timecourse indicated, in the presence of an extracellular medium containing 2.5 mM L-Tyr (squares) or extracellular medium lacking of L-Tyr (circles). Data show the mean efflux and retention ± standard deviation in 3 replicates (measuring efflux/retention from 5 oocytes each) from a single experiment, and are representative of 3 independent experiments. (D) *Trans*-stimulated initial rate kinetic analysis of L-Tyr transport by *Tg*ApiAT5-3. The rate of L-Tyr uptake was measured at a range of [L-Tyr] concentrations in the external medium (i.e. [L-Tyr]_cis_) in *Tg*ApiAT5-3-expressing oocytes preloaded with 0 mM to 2.5 mM L-Tyr (i.e. [L-Tyr]_trans_). The *Tg*ApiAT5-3-mediated uptake (calculated by subtracting the uptake in uninjected oocytes from the uptake in *Tg*ApiAT5-3-expressing oocytes) at each [L-Tyr]_trans_ condition tested conformed to a Michaelis-Menten kinetic model (R^2^ > 0.90 for all non-linear regressions). The data were fitted to a Scatchard linear regression (0.89 ≤ R^2^ ≤ 0.98 for all linear regressions). Data show the mean uptake rate ± standard deviation in 10 oocytes from a single experiment, and are representative of 2 independent experiments.

To characterize the substrate specificity of *Tg*ApiAT5-3 further, we expressed HA-tagged *Tg*ApiAT5-3 in *Xenopus laevis* oocytes, and confirmed its expression and plasma membrane localization by western blotting (S6A Fig). Given the GC-MS data implicating *Tg*ApiAT5-3 in L-Tyr uptake (Fig 4), we hypothesised that *Tg*ApiAT5-3 transports L-Tyr. We compared the uptake of radiolabelled [^14^C]-tyrosine ([^14^C]Tyr) into oocytes expressing *Tg*ApiAT5-3 relative to uninjected oocytes. Under the conditions of the experiment, there was a significant, 7-fold increase in the initial uptake rate of [^14^C]Tyr into oocytes expressing *Tg*ApiAT5-3 compared to uninjected control oocytes (Fig 5A).

To investigate the possibility that *Tg*ApiAT5-3 has exchange activity, we tested whether the uptake of [^14^C]Tyr was stimulated by the presence of amino acids on the *trans* side of the membrane (i.e. *inside* the oocyte). Following preliminary experiments to optimise the preloading of L-Tyr into oocytes, we measured [^14^C]Tyr uptake over 2 hr at an extracellular concentration of 1 mM L-Tyr in *Tg*ApiAT5-3 cRNA-injected or uninjected oocytes that had been pre-loaded in medium containing 2.5 mM unlabelled L-Tyr. The initial rate of [^14^C]Tyr uptake in *Tg*ApiAT5-3-expressing oocytes preloaded with L-Tyr was 3-fold higher than in *Tg*ApiAT5-3-expressed oocytes that were not pre-loaded (Fig 5B), indicating that L-Tyr uptake into *Tg*ApiAT5-3-expressing oocytes was stimulated by L-Tyr on the *trans* side of the membrane.

We next tested whether L-Tyr efflux was also *trans*-stimulated. We preloaded *Tg*ApiAT5-3-expressing and uninjected oocytes with radiolabelled L-Tyr and measured the efflux and retention of the radiolabel upon the addition of 2.5 mM unlabelled L-Tyr to the external medium. [^14^C]Tyr efflux was increased, and [^14^C]Tyr retention reduced, in *Tg*ApiAT5-3-expressing oocytes exposed to 2.5 mM L-Tyr compared to those in external medium lacking L-Tyr (Fig 5C). Nevertheless, there was some [^14^C]Tyr efflux over time in the absence of *trans*-substrate (Fig 5C), indicating that *Tg*ApiAT5-3 can mediate unidirectional flux of L-Tyr. We observed no differences in [^14^C]Tyr efflux or retention in control uninjected oocytes upon incubation of oocytes in 2.5 mM L-Tyr compared to incubation in buffer lacking L-Tyr (S6B Fig). These data indicate that the transporter operates more effectively under ‘exchange conditions’ than under conditions in which it is mediating a unidirectional flux.

To examine the exchange activity of *Tg*ApiAT5-3 further, we investigated the kinetic properties of L-Tyr transport in more detail. Steady-state kinetic parameters for exchangers must be conducted at different *trans*- and *cis*-substrate concentrations to determine accurate K_0.5_ values [26]. We examined the kinetics for the uptake of [^14^C]Tyr following the preloading of oocytes with different L-Tyr concentrations (note that this, and all subsequent, uptake measurements were conducted over 10 min, which falls within the initial, approximately linear, phase of the influx timecourse; Fig 5B). Both Michaelis-Menten analysis (Table 1) and Scatchard linear regressions (Fig 5D) demonstrated that the apparent K_0.5_ values for L-Tyr uptake into oocytes was unaffected by the cytosolic L-Tyr concentration. The gradients of fitted Scatchard plots are negative reciprocals of the apparent K_0.5_ and, therefore, equivalent slopes reflect similar apparent substrate affinities. K_0.5_ values were consistent for both Scatchard and Michaelis-Menten plots derived from the same data, ranging from 0.25 to 0.40 μM (Table 1). As expected for *trans*-stimulated uptake, maximum rate (V_max_) values increased in proportion to the concentration of preloaded L-Tyr (Fig 5D; Table 1).

**Table 1:** Michaelis-Menten kinetic parameters for the initial rate of L-Tyr uptake by *Tg*ApiAT5-3^a^.

[L-Tyr]_*trans*_ (mM)	Scatchard Linear Regression		Michaelis-Menten Plot	
	*K*_0.5_ (μM)	*V*_*max*_ (pmol/min × oocyte)	*K*_0.5_ (μM)	*V*_*max*_ (pmol/min × oocyte)
0	0.36	5.23	0.37	5.43
0.02	0.37	5.70	0.31	6.06
0.08	0.38	6.25	0.29	7.91
0.15	0.34	9.13	0.25	8.93
0.44	0.40	18.83	0.32	15.98
1.78	0.39	29.25	0.36	27.0

^a^ Values are based on the experiment shown in Fig 5D.

To assess whether *Tg*ApiAT5-3 might function as an ion:amino acid co-transporter, we conducted systematic ion-replacement uptake experiments as described previously [9]. We observed no significant change in [^14^C]Tyr uptake under any ion-replacement conditions, suggesting that the transporter does not co-transport any of the ions tested (S6C Fig). To determine whether *Tg*ApiAT5-3 is an electrogenic transporter (as is the case for *Tg*ApiAT1; [9]), we perfused oocytes expressing *Tg*ApiAT5-3 with L-Tyr and measured the electrical current across the oocyte plasma membrane using a two-electrode voltage clamp configuration. We observed no net current under any of the conditions tested, including those in which the membrane potential and pH gradient across the membrane were altered (S6D Fig). Together, these data indicate that L-Tyr transport by *Tg*ApiAT5-3 does not co-transport any charged species.

**Fig 6:**
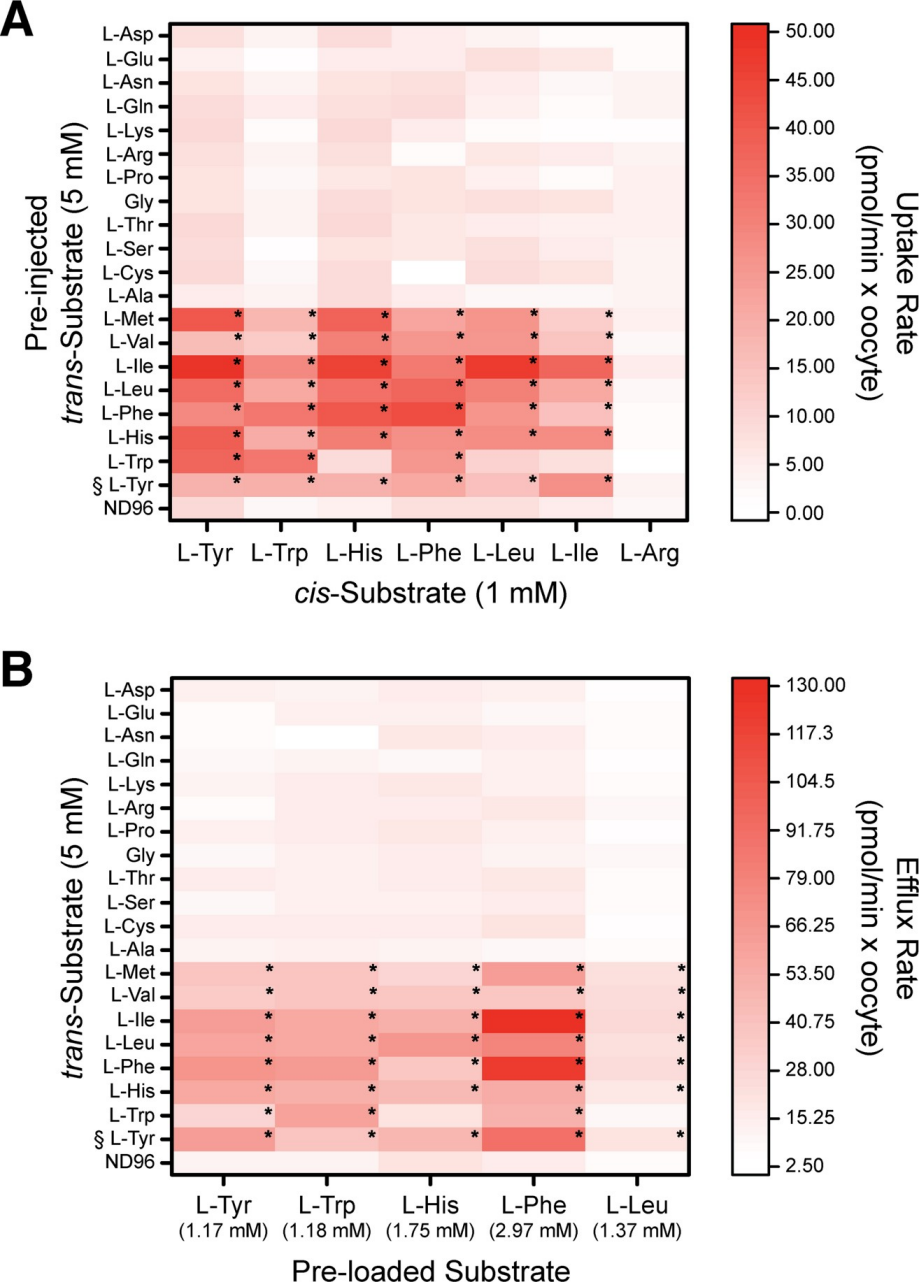
*Tg*ApiAT5-3 is an exchanger for aromatic and large neutral amino acids. (A) *Tg*ApiAT5-3-expressing oocytes were pre-injected with a range of L-amino acids at a calculated oocyte cytosolic concentration of 5 mM (with the exception of L-Tyr (§) which was preloaded into oocytes via incubation in 2.5 mM L-Tyr for 32 hr), or were not pre-injected (ND96 condition). Subsequent uptake of 1 mM L-amino acids containing 0.5 or 1 μCi/ml [^14^C]-labelled amino acid (*cis*-Substrate) was measured over 10 minutes and normalised to uptake per minute. Each box in the heat map shows the mean uptake in 10 oocytes from a single experiment, representative of 3 independent experiments. The statistical analyses compare pre-injected/pre-loaded oocytes to ND96 controls for each substrate tested (*, P < 0.05, one-way ANOVA, Dunnet’s post-hoc test. Where significance values are not shown, the differences are not significant, P > 0.05). (B) *Tg*ApiAT5-3-expressing oocytes were preloaded with a range of L-amino acids containing 0.5 or 1 μCi/ml [^14^C] radiolabelled amino acids (calculated final concentrations shown beneath each substrate), and efflux of these substrates was measured over 5 min in the absence of external amino acids (ND96) or in the presence of 5 mM external amino acids (with the exception of L-Tyr (§), which was present at a concentration of 2.5 mM), and normalised to efflux per minute. Each box in the heat map shows the mean rate of efflux from 3 replicates (each comprised of 5 oocytes) from a single experiment, representative of 3 independent experiments. Statistical analyses compare trans substrates to ND96 controls for each efflux substrate tested (*, P < 0.05, one-way ANOVA, Dunnet’s post-hoc test. Where significance values are not shown, the differences are not significant, P > 0.05).

Incubation of WT and *apiAT5-3*^*Δ188–504*^ parasites in a [^13^C]-amino acid mix resulted in significant increases or decreases in the ^13^C-labelling of several amino acids (Fig 4), suggesting that *Tg*ApiAT5-3 may transport a range of amino acids. To investigate the substrate specificity of *Tg*ApiAT5-3 further, we measured the uptake of 500 μM [^14^C]Tyr in oocytes expressing *Tg*ApiAT5-3 in the presence of equimolar amounts of unlabelled L-amino acids on the *cis* side of the membrane. Only unlabelled L-tryptophan (L-Trp) caused a significant reduction of L-Tyr uptake under the conditions tested (S7A Fig). To examine the *trans*-stimulated influx specificity of *Tg*ApiAT5-3, we pre-injected mixtures of L-amino acids and other metabolites into oocytes expressing *Tg*ApiAT5-3, then measured uptake of [^14^C]Tyr. *Tg*ApiAT5-3-mediated [^14^C]Tyr uptake was *trans*-stimulated by pre-injected mixtures of L-amino acids, but not amino acid derivatives, D-amino acids, nucleotides, nitrogenous bases, or sugars (S7B Fig; S4 Table). To identify which amino acids might be responsible for this *trans*-stimulation, we pre-injected oocytes expressing *Tg*ApiAT5-3 with 19 of the 20 proteinogenic amino acids, each to a final concentration of 5 mM. The one exception was L-Tyr which, in light of its low solubility, was preloaded to equilibrium instead of being injected. After pre-injecting or preloading of the amino acids, we measured the *trans*-stimulated uptake of [^14^C]Tyr. We found that aromatic and large neutral amino acids, but not smaller or charged amino acids, *trans*-stimulated [^14^C]Tyr uptake (Fig 6A).

We tested the uptake of a range of [^14^C]-labelled aromatic and large neutral amino acids, including L-Trp, L-histidine (L-His), L-phenylalanine (L-Phe), L-leucine (L-Leu) and L-isoleucine (L-Ile), into oocytes expressing *Tg*ApiAT5-3. In the absence of a trans substrate, the rates of uptake of aromatic and large neutral amino acids were significantly increased compared to uninjected oocytes (1.7-fold increase for L-Trp, 3.1-fold increase for L-His, 5.5-fold increase for L-Phe, 2.2-fold increase for L-Leu, and 2.4-fold increase for L-Ile; S7C Fig). The uptake of the cationic amino acid L-Arg did not differ between *Tg*ApiAT5-3 injected and uninjected oocytes (S7C Fig). As was seen for L-Tyr, uptake of the aromatic and large neutral amino acids tested were *trans*-stimulated by aromatic and large neutral amino acids, and not by smaller or charged L-amino acids (Fig 6A). The uptake of L-Arg was not *trans*-stimulated by any of the amino acids tested (Fig 6A). We next measured efflux of preloaded [^14^C]-labelled L-Tyr, L-Trp, L-His, L-Phe and L-Leu in the presence of all 20 proteinogenic amino acids in the extracellular medium. We observed the same substrate specificity, with aromatic and large neutral L-amino acids stimulating the efflux of the [^14^C]-labelled substrates tested (Fig 6B).

From the experiments conducted in this section, we conclude that *Tg*ApiAT5-3 mediates the transport of aromatic and large neutral amino acids. The transporter can function as a uniporter, but has a strong propensity for exchange, implying a role for *Tg*ApiAT5-3 in the homeostasis of a range of aromatic and large neutral amino acids.

### *Tg*ApiAT5-3 is important for tyrosine uptake into parasites

The [^13^C] amino acid uptake data and oocyte experiments indicate a role for *Tg*ApiAT5-3 in L-Tyr uptake into the parasite (Fig 4; Fig 5). We set out to test the importance of *Tg*ApiAT5-3 for the uptake of L-Tyr and other amino acids and, subsequently, its importance for parasite growth and virulence. Preliminary experiments indicated that the parental strain used to make the original *apiAT5-3*^*Δ188–504*^ strain was avirulent in mice, precluding its use for virulence studies. We therefore remade the *apiAT5-3*^*Δ188–504*^ mutant in virulent RHΔ*hxgprt*/Tomato strain parasites (S2 Table; [27]), and performed all subsequent experiments with this strain.

**Fig 7:**
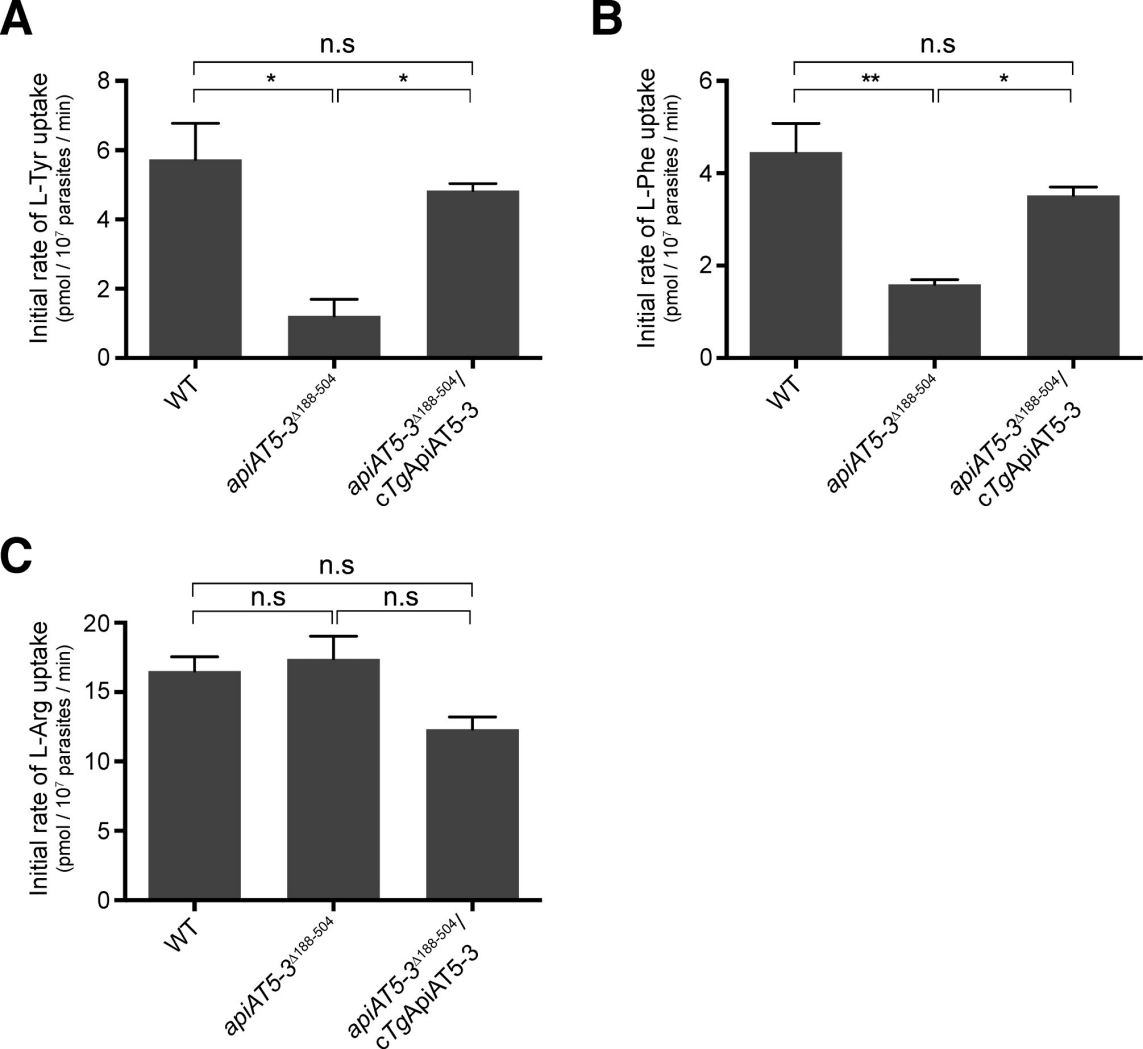
*Tg*ApiAT5-3 mediates the uptake of L-tyrosine and L-phenylalanine into *T*. *gondii*. Initial rate of uptake of (A) [^14^C]Tyr, (B) [^14^C]Phe, and (C) [^14^C]Arg, in WT (RHΔ*hxgprt*/Tomato), *apiAT5-3*^*Δ188–504*^, and *apiAT5-3*^*Δ188-504*^/c*Tg*ApiAT5-3 strain parasites. Uptake was measured in PBS-glucose containing either 60 μM unlabelled L-Tyr and 0.1 μCi/ml [^14^C]Tyr (A), 15 μM unlabelled L-Phe and 0.1 μCi/ml [^14^C]Phe (B), or 100 μM unlabelled L-Arg and 0.1 μCi/ml [^14^C]Arg (C). The initial rates of transport for each substrate were computed from the initial slopes of the fitted single-order exponential curves (S8 Fig), and represent the mean ± SEM from three independent experiments (* *P*<0.05; ** *P*<0.01; n.s. = not significant; Student’s *t* test).

To test the importance of *Tg*ApiAT5-3 for L-Tyr uptake in *T*. *gondii*, we measured the kinetics of [^14^C]Tyr uptake in WT (RHΔ*hxgprt*/Tomato) and *apiAT5-3*^*Δ188–504*^ parasites. The initial rate of [^14^C]Tyr uptake in *apiAT5-3*^*Δ188–504*^ parasites was decreased by 4.7-fold compared to that in WT parasites (Fig 7A; S8A Fig). Both [^14^C]Tyr uptake and parasite growth were restored upon complementation of the *apiAT5-3*^*Δ188–504*^ mutant with a constitutively expressed copy of *Tg*ApiAT5-3 (*apiAT5-3*^*Δ188-504*^/c*Tg*ApiAT5-3; Fig 7A; S9 Fig).

Our previous data indicated that *Tg*ApiAT5-3 can transport aromatic amino acids other than L-Tyr (Fig 6, S7A Fig). We measured uptake of [^14^C]Phe in WT and *apiAT5-3*^*Δ188–504*^ parasites. We observed a significant, 2.8-fold decrease in the initial rate of [^14^C]Phe uptake in parasites lacking *Tg*ApiAT5-3, and this was restored upon *Tg*ApiAT5-3 complementation (Fig 7B; S8B Fig). We were unable to detect robust levels of uptake of [^14^C]Trp in either WT or *apiAT5-3*^*Δ188–504*^ parasites, precluding analysis of the role of *Tg*ApiAT5-3 in uptake of this amino acid into parasites. As a control, we measured uptake of [^14^C]Arg, which is not transported by *Tg*ApiAT5-3 (Fig 6; S7A Fig), in WT, *apiAT5-3*^*Δ188–504*^ and *apiAT5-3*^*Δ188-504*^/c*Tg*ApiAT5-3 strain parasites. The rate of [^14^C]Arg uptake did not differ significantly between these parasite lines (Fig 7C; S8C Fig), indicating that the defect we observed in the uptake of aromatic amino acids is specific to this class of amino acids, and does not represent a general defect in amino acid uptake.

### Growth of parasites lacking *Tg*ApiAT5-3 is modulated by the concentrations of aromatic amino acids in the growth medium

**Fig 8:**
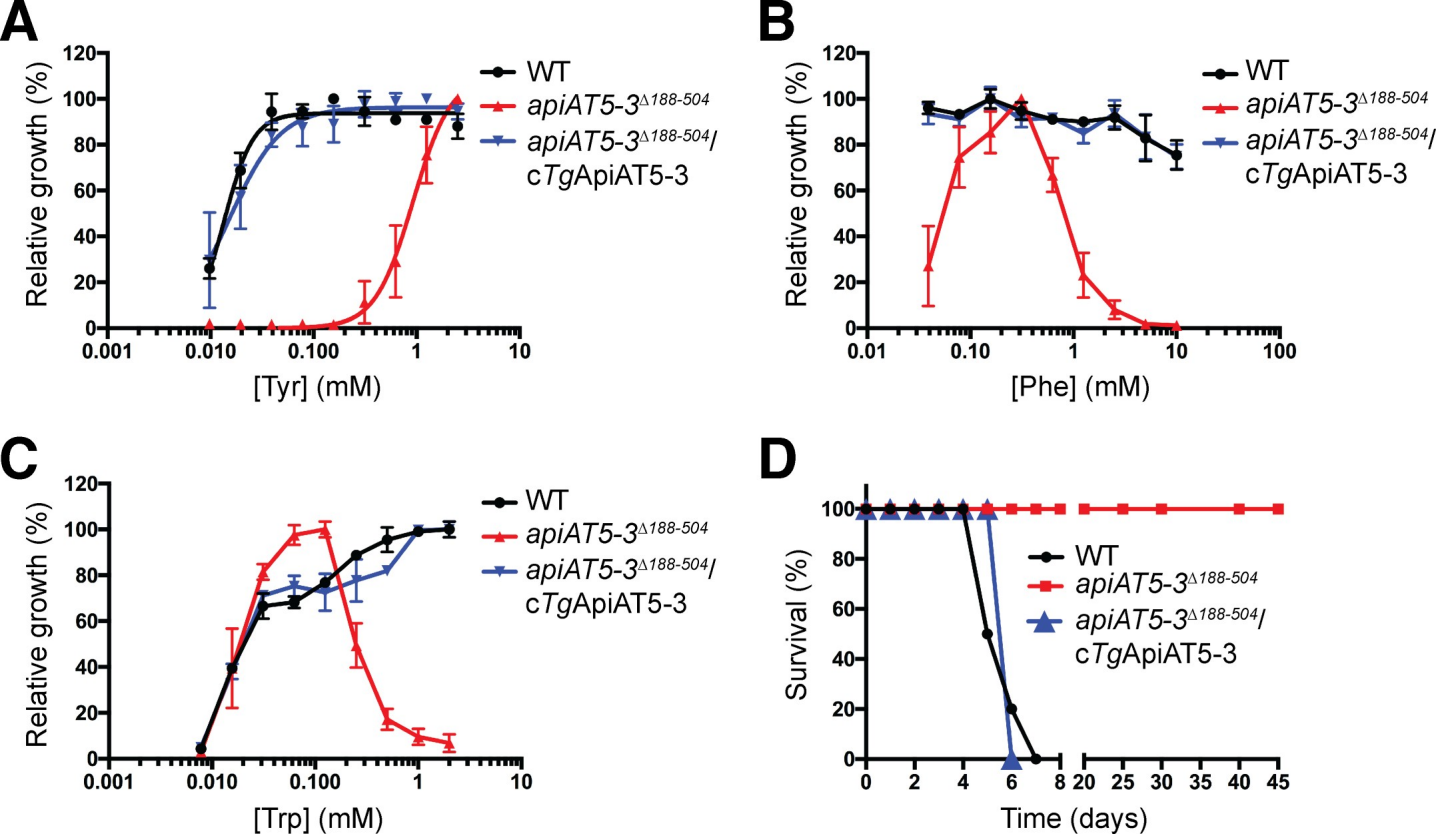
*In vitro* growth of parasites lacking *Tg*ApiAT5-3 is modulated by the concentration of aromatic amino acids in the growth medium, and *Tg*ApiAT5-3 is important for parasite virulence. (A-C) Fluorescence growth assay for WT (RHΔ*hxgprt*/Tomato, black), *apiAT5-3*^*Δ188–504*^ (red) and *apiAT5-3*^*Δ188-504*^/c*Tg*ApiAT5-3 strain parasites cultured for 5 days in DMEM containing a range of L-Tyr (A), L-Phe (B), or L-Trp (C) concentrations. Growth is expressed as a percentage of maximum growth measured on day 5 for each parasite strain. Sigmoidal curves were fitted to the data in (A). All data shown are averaged from three biological replicates (mean ± SEM). (D) Balb/c mice were infected intraperitoneally with 1,000 WT (black), *apiAT5-3*^*Δ188–504*^ (red), or *apiAT5-3*^*Δ188-504*^/c*Tg*ApiAT5-3 (blue) strain parasites and monitored for symptoms of toxoplasmosis. Data for WT and *apiAT5-3*^*Δ188–504*^ parasites are derived from 2 biological replicates consisting of 5 mice each, whereas data for *apiAT5-3*^*Δ188-504*^/c*Tg*ApiAT5-3 parasites is derived from a single experiment consisting of 5 mice.

We next investigated the dependence of the growth of *apiAT5-3*^*Δ188–504*^ parasites on the concentration of L-Tyr in the culture medium. WT, *apiAT5-3*^*Δ188–504*^ and complemented (*apiAT5-3*^*Δ188-504*^/c*Tg*ApiAT5-3) parasites were grown in DMEM containing 0–2.5 mM L-Tyr. Growth of all three parasite strains in the absence of L-Tyr was significantly impaired (S10A Fig), consistent with a previous study that indicated *T*. *gondii* parasites are auxotrophic for this amino acid [28]. WT and complemented parasites grew normally in [L-Tyr] as low as 39 μM (Fig 8A). By comparison, growth of *apiAT5-3*^*Δ188–504*^ parasites was negligible at [L-Tyr] of 156 μM and below, and severely impaired at concentrations below 1 mM (Fig 8A). We also measured growth of WT, *apiAT5-3*^*Δ188–504*^ and complemented parasites grown in DMEM vs DMEM containing 2.5 mM L-Tyr, by plaque assay. Both WT and complemented parasites grew normally in both media (S9 Fig). Consistent with the results of the fluorescence growth assays, the growth of *apiAT5-3*^*Δ188–504*^ parasites was restored to wild type levels by growth in 2.5 mM L-Tyr (S9 Fig).

These data indicate that uptake of L-Tyr via *Tg*ApiAT5-3 is important for the growth of *T*. *gondii* parasites in standard *in vitro* conditions. They also point to the existence of an alternative L-Tyr uptake pathway that can mediate sufficient L-Tyr uptake to rescue growth of *apiAT5-3*^*Δ188–504*^ parasites if the culture medium contains ≥ 1 mM L-Tyr.

To assess the physiological importance of *Tg*ApiAT5-3-mediated L-Phe uptake for parasite growth, WT, *apiAT5-3*^*Δ188–504*^ and complemented parasites were cultivated in medium containing 0 to 10 mM L-Phe (at a constant [L-Tyr] of 2.5 mM). Both WT and complemented parasites showed minimal growth in the absence of L-Phe (S10B Fig), indicating that *T*. *gondii* is auxotrophic for this amino acid, but both lines grew normally at [L-Phe] of 39 μM and above (Fig 8B). By contrast, growth of *apiAT5-3*^*Δ188–504*^ parasites was impaired at 39 μM [L-Phe] (Fig 8B). *apiAT5-3*^*Δ188–504*^ parasites grew optimally at [L-Phe] between 78 μM and 625 μM, but parasite growth decreased at [L-Phe] of 1.25 mM and above (Fig 8B). These data indicate that *Tg*ApiAT5-3 is required for parasite growth at both low and high exogenous concentrations of L-Phe, but not at intermediate concentrations. This points to the existence of other L-Phe uptake pathways in the parasite.

We next measured the growth of WT, *apiAT5-3*^*Δ188–504*^ and complemented parasites in medium containing 0–2 mM L-Trp (and a constant [L-Tyr] of 2.5 mM). WT and complemented parasites exhibited minimal growth in the absence of L-Trp (S10C Fig), consistent with a previous study that indicated *T*. *gondii* is auxotrophic for this amino acid [29]. WT and complemented parasites grew optimally at [L-Trp] above approximately 0.5 mM, and exhibited decreased growth in [L-Trp] below 0.5 mM (Fig 8C). By contrast, *apiAT5-3*^*Δ188–504*^ parasites grew optimally at 31–125 μM L-Trp (Fig 8C). Growth of *apiAT5-3*^*Δ188–504*^ parasites was negligible below 8 μM L-Trp, and decreased in [L-Trp] of 250 μM and above (Fig 8C). Together, these data reveal that *Tg*ApiAT5-3 is required for parasite growth at high exogenous L-Phe and L-Trp concentrations, consistent with high concentrations of L-Phe or L-Trp competitively inhibiting uptake of L-Tyr via an alternative, non-*Tg*ApiAT5-3 L-Tyr uptake pathway(s). This is considered further in the Discussion.

### *Tg*ApiAT5-3 is important for parasite virulence

Standard media formulations used to culture parasite *in vitro* do not necessarily reflect the amino acid concentrations that parasites encounter *in vivo*. Since the importance of *Tg*ApiAT5-3 for parasite growth *in vitro* varied with the concentrations of aromatic amino acids in the growth medium (Fig 8A–8C), we investigated the importance of *Tg*ApiAT5-3 for parasite virulence in a mouse infection model. We infected BALB/c mice intraperitoneally with 10^3^ WT (RHΔ*hxgprt*/Tomato), *apiAT5-3*^*Δ188–504*^ or complemented (*apiAT5-3*^*Δ188-504*^/c*Tg*ApiAT5-3) parasites and monitored disease progression. Mice infected with WT and complemented parasites exhibited symptoms of toxoplasmosis and were euthanized 5–7 days post-infection (Fig 8D). By contrast, mice infected with *apiAT5-3*^*Δ188–504*^ parasites exhibited no symptoms of toxoplasmosis across the entire 45 days of the experiments (Fig 8D), indicating that *Tg*ApiAT5-3 is essential for parasite virulence.

## Discussion

Parasites must scavenge essential amino acids from their environment, although, in the case of apicomplexan parasites, how they do so is poorly understood. We showed recently that uptake of cationic amino acids in *T*. *gondii* and *P*. *berghei* is mediated by members of a family of polytopic membrane transporters [9]. Here, we have investigated the function of other members of this family and show that one of these proteins, *Tg*ApiAT5-3 functions is an aromatic amino acid transporter. Based in these findings, we have renamed this family of proteins Apicomplexan Amino acid Transporters (ApiATs).

The ApiAT protein family is found in all apicomplexan species that we analysed. The similarity of ApiATs to mammalian LAT3/4-type amino acid transporters suggests that the ancestral function of ApiATs was amino acid transport. The ApiATs appear to have undergone expansion in various apicomplexan lineages, including *Plasmodium* spp, *T*. *gondii* and piroplasms such as *Babesia* spp and *Theileria* spp, whereas only a single representative is present in *Cryptosporidium* spp, an early-diverging lineage of apicomplexans [30]. These observations are consistent with ancestral apicomplexans having a single ApiAT protein that diversified in various lineages of the phylum to encompass new and/or more discriminating amino acid substrate selectivities.

A similar expansion has been observed in the amino acid/auxin permease (AAAP) family of trypanosomatid parasites, in which fourteen AAAP genes arose from a single AAAP gene locus through a series of gene duplication events in ancestral trypanosomatids [31]. AAAP expansion is likely to reflect an early parasitic innovation that contributed to establishing parasite dependency on the host organism, and thereby facilitating the evolution of parasitism in trypanosomatids [31, 32]. By contrast, much of the expansion in the ApiAT family appears to have occurred subsequent to the diversification of the major lineages in the phylum. Of the eleven ApiAT subfamilies that we define, only the ApiAT2 subfamily is broadly distributed amongst the major apicomplexan lineages (Fig 1), suggesting its presence before these lineages diverged. Several subfamilies have undergone expansion within lineages. For example, the ApiAT3, 5, 6 and 7 subfamilies contain multiple members within coccidians (*T*. *gondii*, *N*. *caninum* and *E*. *tenella*), while piroplasms contain multiple ApiAT2 subfamily proteins (Fig 1).

**Fig 9:**
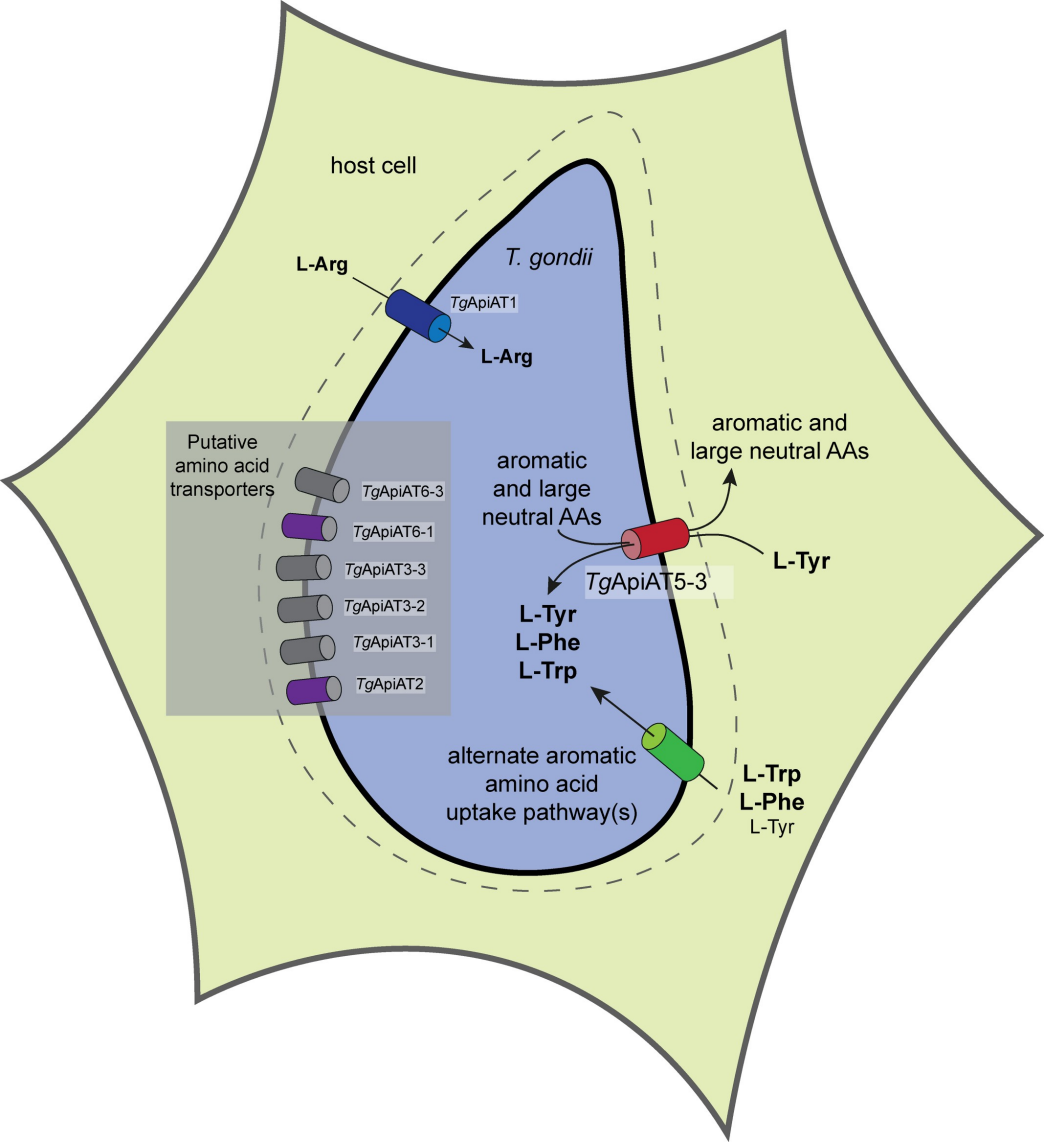
Model for the roles of ApiAT proteins in *T*. *gondii* tachyzoites. Depiction of a *T*. *gondii* parasite (blue) inside a host cell. Aromatic amino acids, including L-Tyr, L-Phe and L-Trp, and large neutral amino acids are thought to be translocated across the parasitophorous vacuole membrane surrounding the parasite (dashed line) through non-selective channels [64]. *Tg*ApiAT5-3 (red cylinder) functions as the major L-Tyr uptake pathway in *T*. *gondii*. Additionally, *Tg*ApiAT5-3 functions as an exchanger, exporting aromatic and large neutral amino acids from the parasite, and thereby contributing to the homeostasis of these amino acids. The uptake of L-Phe and L-Trp is primarily mediated by alternate, and as yet undefined, uptake pathways (green cylinder). These alternate pathways can mediate sufficient L-Tyr uptake for parasite growth in the absence of *Tg*ApiAT5-3 at high L-Tyr concentrations (when L-Phe and L-Trp concentrations are not correspondingly high). We have previously demonstrated that *Tg*ApiAT1 (blue cylinder) facilitates the uptake of L-Arg into parasites [9], and propose that other ApiAT-family proteins expressed in tachyzoites (gray and purple cylinders) facilitate the transport of other amino acids. Of these, *Tg*ApiAT2 and *Tg*ApiAT6-1 (purple cylinders) are important for parasite growth.

Much of the expansion of ApiAT proteins, then, appears to have occurred subsequent to the evolution of parasitism in this phylum. An intriguing possibility is that expansion within ApiAT subfamilies is linked to expansion of these parasites into different hosts, and cell types within those hosts. Across their life cycles, apicomplexans such as *T*. *gondii*, *Plasmodium* spp and piroplasms must infect different hosts and/or different cell types with those hosts, which may necessitate amino acid transporters with different substrate affinities and specificities. Our data indicate that ten of the sixteen ApiAT family proteins in *T*. *gondii* are expressed in the tachyzoite stage of the life cycle (Fig 2; [9]). Of the ApiAT5 subfamily, we could only detect expression of *Tg*ApiAT5-3 in tachyzoites (Fig 2), and only *Tg*ApiAT5-3 is important for growth of the tachyzoite stage (Fig 3). This raises the possibility that other *Tg*ApiAT5 subfamily proteins are expressed, and function, at other stages of the life cycle. Interestingly, proteomic studies identified *Tg*ApiAT5-5 in the oocyst proteome (www.toxodb.org), and it could be that this transporter has particular importance at this stage of the parasite life cycle.

Of the fifteen ApiAT family proteins that we were able to disrupt genetically, only the *Tg*ApiAT1, *Tg*ApiAT2 and *Tg*ApiAT5-3 mutants exhibited defects in tachyzoite growth (Fig 3). This corresponds to results from a recent genome-wide CRISPR-based screen, in which these three *Tg*ApiAT family proteins all had low ‘phenotype scores’ (scores between –3.91 and –4.73), an indicator of a gene’s importance for *in vitro* growth of tachyzoites ([33]; scores below –1.8 are considered to be indicative of a gene being ‘important’ for parasite growth). The remaining *Tg*ApiAT family proteins had phenotype scores > –0.93 [33], consistent with the results of our targeted disruption approach which indicated that these proteins are not important for parasite growth *in vitro* (Fig 3). However, we cannot rule out that parasites were able to ‘skip’ over the frameshifts to produce functional proteins in these mutants, or that parasites were able to adapt to these loss of function mutants (e.g. by upregulating other transporters to compensate for their loss). We were unable to disrupt the reading frame of *Tg*ApiAT6-1, despite multiple attempts using a guide RNA that targets the *Tg*ApiAT6-1 locus (S2 Table). *Tg*ApiAT6-1 has a phenotype score of –5.4 [33]. It is likely, then, that our inability to generate a *Tg*ApiAT6-1 gene disruption is because it is essential for parasite growth.

Our studies of *Tg*ApiAT5-3 demonstrate that this protein is important for parasite growth. We demonstrated that *Tg*ApiAT5-3 is a high affinity L-Tyr uniporter (Fig 5; K_0.5_ ~0.3 μM, Table 1) and that loss of *Tg*ApiAT5-3 leads to defects in L-Tyr uptake into parasites (Fig 4, Fig 7A). These observations are supported by the results of a recent study by Wallbank *et al*., who also demonstrated that disruption of *Tg*ApiAT5-3 led to a depletion of L-Tyr levels in the parasite, and replicated our initial observations [34] that *Tg*ApiAT5-3 can transport L-Tyr [35]. In our study, we demonstrate that parasites lacking *Tg*ApiAT5-3 were avirulent in mice (Fig 8D), and could only grow at extracellular L-Tyr concentrations above ~1 mM (Fig 8A; S9 Fig), well above the plasma concentration of L-Tyr in mammals (estimated to be 55–90 μM in human plasma and 50–70 μM in mouse plasma; [36, 37]). Together, our data point to an essential role for *Tg*ApiAT5-3 in scavenging L-Tyr from the host.

Our oocyte studies indicated that *Tg*ApiAT5-3 can function as an exchanger, with the rate of uptake of L-Tyr and other aromatic and large neutral amino acids enhanced when equivalent amino acids were present on the *trans* side of the membrane (Fig 6). Mammalian amino acid transporters function either as ‘loaders’ (i.e. uniporters that facilitate the uptake of amino acids into cells) or ‘harmonizers’ (i.e. exchangers that are essential for the maintenance of homeostatic amino acid concentrations) [38]. By contrast, our data indicate that *Tg*ApiAT5-3 performs an unusual dual function in facilitating both the net uptake of L-Tyr into the parasite, and maintaining intracellular pools of aromatic and large neutral amino acids through exchange. Maintaining amino acid homeostasis is critical for facilitating cellular metabolism and growth, and it is likely that *Tg*ApiAT5-3 has a critical role in balancing the intracellular concentrations of aromatic and large neutral amino acids in the parasite.

*X*. *laevis* expression studies revealed that *Tg*ApiAT5-3 transports L-Phe and L-Trp, as well as large neutral amino acids such as L-Leu (Fig 6). This raises the possibility that *Tg*ApiAT5-3 also functions in the net uptake of these amino acids in the parasite. We saw no differences in the fractional labelling of [^13^C]-labelled L-Leu or L-Ile in *apiAT5-3*^*Δ188–504*^ parasites compared to WT parasites (Fig 4), implying that the uptake of these branched-chain amino acids is facilitated by other transporters in the parasite. Similarly, we observed no defect in the fractional labelling of [^13^C]-labelled L-Phe in parasites lacking *Tg*ApiAT5-3 (Fig 4), although we did observe a defect in the uptake of [^14^C]Phe in *apiAT5-3*^*Δ188–504*^ parasites (Fig 7C), and we observed decreased growth of the mutant strain at [L-Phe] of 39 μM and below (Fig 8B). Notably, uptake of [^14^C]Tyr into oocytes expressing *Tg*ApiAT5-3 was not impaired by the addition of equimolar amounts of unlabelled L-Phe (S7A Fig), indicating that *Tg*ApiAT5-3 has a greater affinity for L-Tyr than L-Phe. If a transporter’s affinity for L-Tyr is much greater than that for L-Phe, uptake of the latter will be minimal in conditions where the amino acids are present at similar concentrations (as we observed in the oocyte experiments, and as appears to be the case in mammalian cells [36]). This is consistent with the hypothesis that *Tg*ApiAT5-3 plays little role in net L-Phe uptake at physiological concentrations in the tachyzoite stage of the parasite life cycle. Instead, L-Phe is likely to be taken up via alternative transport pathways (Fig 9).

The importance of *Tg*ApiAT5-3 for L-Trp uptake in *T*. *gondii* is less clear. We were unable to detect the uptake of either [^13^C]Trp or [^14^C]Trp into parasites under different experimental conditions. In oocyte experiments, L-Trp can effectively out-compete L-Tyr for uptake via *Tg*ApiAT5-3 when present in equimolar amounts (S7A Fig), suggesting that *Tg*ApiAT5-3 has similar affinities for L-Trp and L-Tyr.

*apiAT5-3*^*Δ188–504*^ parasites are capable of growth when the concentration of L-Tyr in the growth medium is ≥1 mM (Fig 8A). This indicates the existence of an alternative L-Tyr uptake pathway that takes up sufficient L-Tyr to enable parasite growth when L-Tyr levels are high (Fig 9). By contrast, *apiAT5-3*^*Δ188–504*^ parasites grew normally at intermediate concentrations of L-Phe (78–625 μM) and L-Trp (31–250 μM), but exhibited a dramatic decrease in growth at higher concentrations of both these amino acids (Fig 8B and 8C). These observations are consistent with the hypothesis that the alternative L-Tyr uptake pathway(s) also functions in L-Phe and L-Trp uptake (Fig 9). At high concentrations of L-Phe and L-Trp, uptake of L-Tyr by this alternative pathway(s) is inhibited by competition with the other aromatic amino acids, preventing parasite growth in the absence of *Tg*ApiAT5-3. This mirrors similar observations in our previous study on the alternative L-Arg uptake pathway in the parasite, in which we showed that high levels of other cationic amino acids inhibit parasite growth in the absence of the selective L-Arg transporter [9].

On the basis of the results of this study, we propose a model in which the uptake of L-Tyr into *T*. *gondii* parasites is mediated primarily by *Tg*ApiAT5-3 (Fig 9). The uptake of L-Phe and L-Trp is mediated primarily by one or more alternative aromatic amino acid transporters. These transporters can also transport L-Tyr, and compensate for loss of *Tg*ApiAT5-3 when L-Tyr levels are high and corresponding levels of L-Phe and L-Trp are lower (Fig 9). *Tg*ApiAT5-3 has an additional role in facilitating homeostasis of aromatic amino acids and large neutral amino acids, enabled by its exchange activity (Fig 9).

This model is based on our observations of tachyzoite stage parasites. A recent study examined *T*. *gondii* aromatic amino acid hydroxylase enzymes, which can interconvert L-Phe and L-Tyr [39]. Knockout of these enzymes revealed that they are not required for tachyzoite growth, but are particularly important for producing oocysts following the sexual stages of the parasite life cycle that occur in the felid hosts [39]. Given the differences in aromatic amino acid metabolism between tachyzoite and oocyst stages of the life cycle, it is likely that the nature and requirements for aromatic amino acid transporters differs across the life cycle of the parasite. Future studies that investigate the expression and importance of *Tg*ApiAT5-3 and other aromatic amino acid transporters (perhaps other members of the *Tg*ApiAT5 family) across the entire life cycle will be of particular interest.

In summary, we describe an apicomplexan-specific family of plasma membrane transporters that appear to function primarily in amino acid uptake. Our findings highlight the evolutionary novelties that arise to enable parasites to scavenge essential nutrients from their hosts, as well as highlighting the importance of amino acid scavenging for the growth and virulence of the disease-causing tachyzoites stage of *T*. *gondii*. Future studies that examine the role of other members of this transporter family across the entire life cycle of the parasite will facilitate a better understanding of how these parasites acquire amino acids from their hosts.

## Materials and methods

### Phylogenetic analysis of the ApiAT protein family

We sought to identify orthologues of the five previously identified *Plasmodium falciparum* NPT proteins [10] in the proteomes of the apicomplexans *Plasmodium berghei*, *Toxoplasma gondii*, *Cryptosporidium parvum*, *Eimeria tenella*, *Neospora caninum*, *Babesia bovis* and *Theileria annulata*, and the chromerids *Chromera velia and Vitrella brassicaformis*. First we performed Protein BLAST searches using each *P*. *falciparum* NPT protein against each target species (www.eupathdb.org). We examined all ‘hits’ with an E value of <10. Candidates that had <8 or >15 transmembrane domains were excluded from further analysis, since this would be inconsistent with them being MFS transporters, which typically have 12 transmembrane domains [20]. We also excluded any candidates annotated as proteins that were not transporters, or proteins already identified as members of other transporter families. Each remaining candidate was used as the query sequence for BLAST searches against the *P*. *falciparum* proteome, and we only accepted candidates for which the top match in the reciprocal BLAST search was to one of the five *P*. *falciparum* NPTs. We next undertook BLAST searches with the candidate ApiAT against all organisms (using the NCBI BLAST search tool, excluding proteins in its own genus), and accepted only those candidates where the top hit was to proteins from other apicomplexans/chromerids. Of those, we included only candidate ApiATs where the top hit in members of the other target species was to proteins that had met all of the previous criteria (i.e. were already included as candidates on the list of potential ApiATs). This would theoretically exclude proteins that were not descended from the common ancestor of ApiATs. None of the candidate chromerid proteins met these latter criteria (all had top ‘hits’ in the NCBI BLAST search to proteins outside apicomplexans), and so were excluded from the subsequent phylogenetic analyses. We identified 66 proteins that met all the criteria. The gene IDs and nomenclature of these are listed in S1 Table.

The 66 ApiAT protein sequences were aligned using Clustal Omega Version 1.2.2 [40, 41]. The multiple sequence alignment was edited in Jalview (www.jalview.org) to remove poorly aligned blocks. After sequence editing, 464 residues were left for subsequent phylogenetic analysis using PHYLIP v3.69 (evolution.genetics.washington.edu/phylip/getme.html) as described previously [42]. Briefly, a consensus maximum likelihood tree and bootstrap values were generated by running the alignment file through the ‘seqboot’ program, which was used to generate 300 pseudosamples of the alignment. Next, multiple phylogenetic trees were generated from the pseudosamples using the ‘proml’ tree algorithm, using a randomised order of entry and three jumbles. Finally, the multiple phylogenetic trees were converted to a consensus tree with bootstrap values using the program ‘consense’.

Trees were viewed using the program FigTree (http://tree.bio.ed.ac.uk/software/figtree/) and annotated using Inkscape (https://inkscape.org/en). For visual representation of the alignment, shading for sequence identity was carried out using the TeXshade package for LaTex [43], using similarity mode with the ‘\fingerprint’ command (https://ctan.org/pkg/texshade?lang=en) and a texshade.sty file kindly provided by Dr Eric Beitz (Uni. Kiel).

### Parasite culture

Parasites were maintained in human foreskin fibroblasts (HFFs; a kind gift from Holger Schülter, Peter MacCallum Cancer Centre) cultured at 37°C in a humidified 5% CO_2_ incubator. Unless otherwise noted, parasites were cultured in Dulbecco’s modified Eagle’s medium (DMEM) supplemented with 1% (v/v) fetal calf serum and antibiotics. All fluorescence growth assay experiments were conducted in medium containing dialyzed serum. ‘Homemade’ media were generated as described previously [9], with amino acids at the concentrations found in DMEM, or modified as specified in the text. Amino acid concentrations in the Minimal Amino Acid Medium (MAAM) are specified in S3 Table. *apiAT5-3*^*Δ188–504*^ parasites were grown continuously in DMEM supplemented with 2.5 mM L-Tyr. TATi [44], TATi/Tomato, TATi/Δ*ku80* [45], and RHΔ*hxgprt*/Tomato [27] parasites were used as parental strains for the genetically modified parasites generated in this study.

### Generation of genetically modified *T*. *gondii* parasites

Guide RNA (gRNA)-encoding sequences specific to target genes were introduced into the vector pSAG1::Cas9-U6::sgUPRT (Addgene plasmid # 54467; [25]) using Q5 site-directed mutagenesis (New England Biolabs) as described previously [25]. A list of the forward primers used to generate gRNA-expressing vectors for introducing frame-shift mutations are described in S5 Table. In each instance, the reverse primer 5’-AACTTGACATCCCCATTTAC was used. For generating frameshift mutations in *Tg*ApiAT genes, gRNAs were designed to target the open reading frames of *Tg*ApiAT genes, and transfected into parasites on a vector that also expressed Cas9-GFP. Transfections were performed as described previously [46]. GFP positive parasites were selected and cloned using flow cytometry 2–3 days following transfection using a FACSAria I or FACSAria II cell sorter (BD Biosciences). The region of the candidate genes targeted by the gRNAs were sequenced in clonal parasites, and clones in which the target gene had been disrupted by a frameshift mutation or insertion of a premature stop codon (i.e. where the open reading frame was disrupted) were selected for subsequent analyses. For 3’ replacements, gRNAs were selected to target a region near the stop codon of the gene of interest, using the primers listed in S6 Table. In addition, a donor DNA sequence encoding a 3x HA tag was amplified by PCR to contain 50 bp of flanking sequences homologous to the target gene either side of the stop codon. Template DNA encoding the 3x HA tag was generated as a gBlock (Integrated DNA Technologies), with the sequence listed in S7 Table. Forward and reverse primers used to amplify the HA tag for each target gene are also listed in S7 Table. gRNA-expressing vectors, which simultaneously encode Cas9 fused to GFP, were co-transfected into *T*. *gondii* parasites with the donor DNA sequence. 2–3 days after transfection, GFP-Cas9-expressing parasites were selected and cloned into wells of a 96-well plate by flow cytometry as described above.

3’ replacement plasmids were created to epitope tag *Tg*ApiAT2, *Tg*ApiAT3-2, *Tg*ApiAT3-3, *Tg*ApiAT5-3, *Tg*ApiAT6-1, *Tg*ApiAT6-2 and *Tg*ApiAT7-1 using conventional crossover recombination methods as described previously [47]. Regions of DNA homologous to the 3’ ends of the genes were amplified by PCR using primers described in S8 Table, and ligated into the *Bgl*II and *Avr*II sites of the vector pgCH [9] or the *Pac*I and *Avr*II sites of pLIC-HA_3_-DHFR [47]. Resulting plasmids were linearized in the flanking sequence using restriction enzymes (S8 Table), then transfected into TATi/Δ*ku80* parasites. Parasites were selected on chloramphenicol or pyrimethamine as described [46]. In cases where we were unable to subsequently detect a protein of approximately the expected molecular mass by western blotting, we confirmed correct integration of the HA tag by sequencing of CRISPR-modified 3’ ends (*Tg*ApiATs 5–1, 5–2, 5–4, 5–5 and 5–6), or by PCR screening (*Tg*ApiAT7-1). For assessing HA integration into the *Tg*ApiAT7-1 locus, DNA was extracted from *Tg*ApiAT7-1-HA parasite clones, and used as template in a PCR with the primers 5’-GGCGAAGAGAAGGCGTTG and 5’-GTCATCCCTTTTCTTCGATAA, with the presence of a 2.5 kb band indicative of successful integration (S3C Fig).

To complement the *apiAT2*^*Δ138–588*^ mutant with a constitutively-expressed copy of *Tg*ApiAT2, we amplified the open reading frame of *Tg*ApiAT2 from genomic DNA with the primers 5’-GATCGGATCCAAAATGGCGGCTGCTCAG and 5’-GATCCCTAGGCACAGCGACCTCTGGACTCGGT. We digested the resultant PCR product with *Bam*HI and *Avr*II and ligated this into the *Bgl*II and *Avr*II sites of the pUgCTH_3_ vector [9]. The resultant vector was linearised with *Mfe*I, transfected into *apiAT2*^*Δ138–588*^ parasites and selected on chloramphenicol. To complement the *apiAT5-3*^*Δ188–504*^ mutant with a constitutively-expressed copy of *Tg*ApiAT5-3, we amplified the open reading frame of *Tg*ApiAT5-3 with the primers 5’-GATCGGATCCAAAATGGAGTCGACCGAGGCGACTAT and 5’-GATCCCTAGGCAGCACCTTCGGGACTTTTCTCTTC, using the *Tg*ApiAT5-3-expressing oocyte vector (described below) as template. We digested the resultant PCR product with *Bam*HI and *Avr*II and ligated this into the *Bgl*II and *Avr*II sites of the pUgCTH_3_ vector. The resultant vector was linearised with *Mfe*I, transfected into *apiAT5-3*^*Δ188–504*^ parasites and selected on chloramphenicol.

### Immunofluorescence assays and western blotting

Immunofluorescence assays and western blotting were performed as described previously [9]. For western blotting, membranes were probed with rat anti-HA antibodies (clone 3F10, Sigma) at dilutions between 1:1,000 to 1:3,000, mouse anti-GRA8 (a kind gift from Gary Ward, U. Vermont, [48]) at a dilution of 1:80,000, or rabbit anti-*Tg*Tom40 antibodies [49] at 1:2,000 dilution, and horseradish peroxidase (HRP)-conjugated goat anti-rat (sc-2006, Santa Cruz Biotechnology), HRP-conjugated goat anti-mouse (sc-2005, Santa Cruz Biotechnology), or HRP-conjugated goat anti-rabbit (sc-2004, Santa Cruz Biotechnology) antibodies at dilutions of 1:5,000 to 1:10,000.

For immunofluorescence assays, samples were probed with the following primary antibodies: rat anti-HA (clone 3F10, Sigma) at a 1:200 dilution, mouse anti-P30 (clone TP3, Abcam) at a 1:2,000 dilution, rabbit anti-P30 (a kind gift from John Boothroyd, Stanford U) at dilutions between 1:25,000 and 1:90,000, rabbit anti-GFP (a kind gift from Alex Maier, ANU) at a 1:200 dilution, or mouse anti-IMC (a kind gift from Gary Ward, U. Vermont) at a 1:500 dilution. Samples were next probed with the following secondary antibodies: CF488A-conjugated goat anti-rat (SAB4600046, Sigma) at a dilution of 1:500, AlexaFluor 488-conjugated goat anti-rat (4416, Cell Signaling Technology) at dilution of 1:250, AlexFluor488-conjugated goat anti-rabbit (A11008, Life Technologies) at a dilution of 1:500, AlexFluor546-conjugated goat anti-rabbit (A11035, Life Technologies) at a dilution of 1:500, AlexFluor546-conjugated goat anti-mouse (A11030, Life Technologies) at a dilution of 1:500, or AlexFluor647-conjugated goat anti-mouse (A21236, Life Technologies) at a dilution of 1:500.

Fluorescence microscopy was performed on a DeltaVision Elite system (GE Healthcare) using an Olympus IX71 inverted microscope with a 100X UPlanSApo objective lens (NA 1.40). Images were recorded using a CoolSNAP HQ2 camera. Images were deconvolved using SoftWoRx Suite 2.0 software, and images were linearly adjusted for contrast and brightness.

### Parasite growth and virulence assays

To measure parasite growth by plaque assays, either 150 parasites were added to wells of a 6-well plate containing confluent HFFs, or 500–1,000 parasites were added to confluent HFFs in 25 cm^2^ tissue culture flasks. Parasites were grown for 8 to 9 days before fixation and staining with crystal violet as described previously [46].

Fluorescence growth assays were performed as described previously [50, 51], with slight modifications. Briefly, wells of an optical bottom 96-well plate containing confluent HFFs were washed twice in medium lacking L-Tyr, L-Phe or L-Trp. Wells were filled with medium containing a range of L-Tyr, L-Phe or L-Trp concentrations. 2,000 parasites were inoculated into each of these wells, and plates were incubated at 37°C in a 5% CO_2_ incubator. Well fluorescence was measured 5 days post-inoculation in a FluoStar Optima fluorescence plate reader (BMG Labtech), a time point at which WT parasites were in mid-logarithmic stage of growth. Relative growth was expressed as a percentage of the well fluorescence in the optimum amino acid concentration for WT, *apiAT5-3*^*Δ188–504*^ or *apiAT5-3*^*Δ188-504*^/c*Tg*ApiAT5-3 parasites at this time point.

To measure parasite virulence, freshly egressed WT (RHΔ*hxgprt*/Tomato), *apiAT5-3*^*Δ188–504*^ or *apiAT5-3*^*Δ188-504*^/c*Tg*ApiAT5-3 parasites were filtered through a 3 μm polycarbonate filter, washed once in phosphate-buffered saline (PBS), then were diluted to 10^4^ parasites/ml in PBS. 10^3^ parasites were injected intraperitoneally into 7-week-old, female Balb/c mice using a 26-gauge needle. Mice were weighed regularly, and monitored for symptoms of toxoplasmosis (weight loss, ruffled fur, lethargy and hunched posture). Mice exhibiting terminal symptoms of toxoplasmosis were euthanized in accordance with protocols approved by the Australian National University Animal Experimentation Ethics Committee (protocol number A2016/42).

### [^13^C]Amino acid labelling and detection

Freshly egressed WT (TATi/Tomato) or *apiAT5-3*^*Δ188–504*^ tachyzoites (10^8^ parasites each sample) were incubated in 500 μl of amino acid-free Roswell Park Memorial Institute 1640 medium supplemented with 2 mg/ml algal [^13^C]amino acid mix (Cambridge Isotope Laboratories) for 15 minutes at 37°C in a 5% CO_2_ incubator. [^13^C]amino acid labelling was terminated by rapid dilution in 14 ml of ice cold PBS. Parasite metabolites were extracted in chloroform:methanol:water (1:3:1 v/v/v) containing 1 nmol *scyllo*-inositol (Sigma). The aqueous phase metabolites were dried in a heated speedvac concentrator, methoxymated by treatment with 20 mg/ml methoxyamine in pyridine overnight, then trimethylsilylated by treatment with N,O-bis(trimethylsilyl)trifluoroacetamide containing 1% trimethylsilyl for 1 hr at room temperature. Samples were analyzed using GC-MS as described previously [52]. The fractional labelling of all detected amino acids was estimated as the fraction of the metabolite pool containing one or more ^13^C-atoms after correction for natural abundance using the program DExSI, as described previously [53]. Total metabolite counts were normalized to *scyllo*-inositol as an internal standard.

### *Xenopus laevis* oocyte preparation and *Tg*ApiAT5-3 expression

The open reading frame of *Tg*ApiAT5-3 was amplified from RHΔ*hxgprt* strain cDNA template using the primers 5’- GATCACCGGTCCACCATGGAGTCGACCGAGGCGACTAT and 5’- GATCCCTAGGCAGCACCTTCGGGACTTTTCTCTTC. The resultant product was digested with *Age*I and *Avr*II, and ligated into the *Xma*I and *Avr*II sites of the vector pGHJ-HA [9]. The plasmid was linearised by incubation in *Not*I overnight, and complementary RNA (cRNA) encoding HA-tagged *Tg*ApiAT5-3 was prepared for injection into oocytes as previously described [54–56]. *Xenopus laevis* oocytes were surgically removed and prepared for cRNA injection as described [55]. For all transporter assays in oocytes, 15 ng of *Tg*ApiAT5-3 cRNA was micro-injected into stage 5 or 6 oocytes using a Micro4 micro-syringe pump controller and A203XVY nanoliter injector (World Precision Instruments).

### Oocyte surface biotinylation and whole membrane preparation

Oocyte surface biotinylation and whole membrane preparations were performed as described previously [55, 57]. Briefly, for surface biotinylation, 15 oocytes were selected 3–6 days post cRNA injection, washed thrice in ice-cold PBS (pH 8.0), incubated for 45 mins at room temperature in 0.5 mg/ml of EZ-Link Sulfo-NHS-LC-Biotin (Thermo Fisher Scientific), and then washed thrice more in ice-cold PBS. Oocytes were subsequently solubilised in oocyte lysis buffer (20 mM Tris-HCl pH 7.6, 150 mM NaCl, 1% v/v Triton X-100) for 2 hr on ice. Samples were centrifuged at 16,000 *g*, and the supernatant was mixed with 50 μl of streptavidin-coated agarose beads (Thermo Fisher Scientific). The mixture was incubated at 4°C on slow rotation overnight. Beads were washed 4 times with oocyte lysis buffer before elution in SDS-PAGE sample buffer. For whole membrane preparation, 10–25 oocytes were homogenised by trituration in homogenisation buffer (50 mM Tris-HCl pH 7.4, 100 mM NaCl, 1 mM EDTA, protease inhibitors). Homogenised oocytes were centrifuged at 2,000 *g* for 10 min at 4°C and the resulting supernatant further centrifuged for 30 min at 140,000 *g* at 4°C. The resulting pellet was washed with homogenisation buffer and solubilised in homogenisation buffer containing 4% (w/v) SDS, and then in SDS-PAGE sample buffer. Protein samples from surface biotinylation and whole membrane preparations were separated by SDS-PAGE then detected by western blotting as described above.

### Oocyte assays: Uptake, efflux and electrophysiology

For uptake experiments in either non-preloaded, preloaded, or pre-injected oocytes, batches of 10 oocytes were washed 4 times in ND96 buffer (96 mM NaCl, 2 mM KCl, 1 mM MgCl_2_, 1.8 mM CaCl_2_, 5 mM HEPES, pH 7.4) at RT, and then incubated in the desired concentration of radiolabelled substrates as indicated in figure legends. For all substrate screening measurements, uptake was measured over 10 mins. For kinetic experiments, parallel batches of oocytes were preloaded with 0–1.78 mM L-Tyr, and uptake of [^14^C]Tyr at a range of L-Tyr concentrations was measured over 10 mins. For other uptake experiments, uptake was measured for the timecourse indicated in the figures. Uptake was quenched by washing oocyte batches four times in ice-cold ND96.

For efflux experiments, batches of 5 oocytes/substrate were preloaded with [^14^C]L-amino acids as described below. Following preloading, oocytes were washed 4 times in ND96 and incubated in *trans*-stimulating substrates at concentrations described in the figure legends. To measure efflux, the 5 oocytes were incubated in 500 μl aliquots of the extracellular solution, of which 100 μl was removed for each time point and the efflux immediately quenched by washing the oocytes four times in ice-cold ND96. Retention of radiolabel within the oocytes was measured by removing the oocytes following quenching of efflux. For the substrate efflux screen depicted in Fig 6B, the extracellular solution was sampled at 5 min, which fell within the initial, approximately linear phase of the uptake timecourse. For efflux experiments depicted in Fig 5C, efflux was measured for the timecourse indicated in the figure.

All oocyte uptake and efflux experiments were performed in solutions containing both [^14^C]-labelled and unlabelled isotopes of the amino acid being studied, with the total substrate concentrations and concentration of [^14^C]-labelled amino acids specified in the relevant figure legend.

Following the relevant incubation periods, oocytes or aliquots were distributed into OptiPlate96-well plates (Perkin-Elmer) and oocytes were lysed overnight in 10% (w/v) SDS. 150 μl/well of Microscint-40 scintillation fluid (Perkin-Elmer) was added to the samples, and plates covered and shaken for 5 min before radioactivity was counted on a Perkin-Elmer MicroBeta^2^ 2450 microplate scintillation counter.

All steady-state electrical recordings were made with an Axon GeneClamp 500B amplifier (Axon Instruments) in a two-voltage clamp configuration as previously described [57, 58]. Voltage clamp was set to −50 mV or 0 mV and data were sampled at 3 Hz using pClamp 8.2 software (Axon Instruments). Boron silicate microelectrodes capillaries (World Precision Instruments) with a tip resistance of: 1.5 ≥ *R*_*e*_ ≥ 0.5 MΩ were pulled by a P-97 Flaming/Brown micropipette puller (Sutter Instruments) and filled with 3 M KCl. Silver microelectrodes were coated using a 5 M NaCl single-chamber galvanic cell to form AgCl_2_ electrodes. The membrane potential was adjusted digitally in voltage-clamp mode between 0 and −50 mV. Oocytes were chosen for recording when they had a resting membrane potential −25 mV < E_m_ < −45 mV. ND96 (pH 7.4) was used as the control solution for all electrophysiological recordings. Assay buffer pH was varied by mixing different ratios of acidic ND96 (pH 3.6) (5 mM MES instead of HEPES) with basic ND96 (pH 10) (5 mM Tris instead of HEPES).

### Preloading of oocytes with substrates

For uptake experiments measuring *trans*-stimulation by a range of amino acids and other metabolites (Fig 6A and S7B Fig), all L-amino acids substrates and metabolite mixes, except for L-Tyr, were pre-injected at 25 nl/oocyte using a Micro4 micro-syringe pump controller and A203XVY nanoliter injector (World Precision Instruments). All pre-injected oocytes were incubated on ice for 30 mins prior to uptake experiments to allow time for recovery from the injection. Stock solutions containing 100 mM L-amino acids in ND96 were pre-injected to give a calculated cytosolic concentration of 5 mM, based on an assumed free aqueous volume of 500 nl/oocyte. Stage 5 or 6 oocytes diameters vary from 1−1.3 mm and free aqueous oocyte volumes measured from 368 to > 500 nl [59, 60]. Therefore, calculations of cytosolic concentrations from pre-injection should be treated as approximations only. For the experiments giving rise to S7B Fig, different metabolite groups were pre-injected to give estimated final concentrations of each metabolite as indicated in S4 Table. The low solubility of L-Tyr in aqueous solutions (0.453 g/L at 25°C, pH 7.4; [61]) necessitated preloading of this amino acid by incubation, rather than injection. L-Tyr solutions were prepared by dissolving L-Tyr in ND96 to a final concentration of 2.5 mM and performing dilutions in ND96 to the required concentrations. Pre-loading of L-Tyr in *Tg*ApiAT5-3-injected oocytes was optimized by monitoring the time-dependent uptake of radiolabelled L-Tyr by oocytes in media containing either 2.5 or 1 mM L-Tyr. In *Tg*ApiAT5-3-expressing oocytes, L-Tyr equilibrium was reached after 10 to 12 hr, while uninjected oocytes reached a similar cytosolic concentration after incubation of approximately 68–72 hr. As a consequence, L-Tyr was preloaded for 32 hr in *Tg*ApiAT5-3 injected oocytes and for 72 hr in uninjected oocytes for all *trans*-stimulation experiments.

All efflux substrate screening with [^14^C]labelled amino acids (Fig 6B) were conducted by preloading L-amino acids for 3 hr prior to uptake. L-Tyr containing 0.5 μCi/ml [^14^C]Tyr was preloaded at a concentration of 2.5 mM, while the other L-amino acids (containing 0.5–1 μCi/ml [^14^C]-labelled substrate) were preloaded at a concentration of 5 mM. Calculation of the pre-loaded [^14^C]labelled L-amino acids concentrations were conducted using a control set of oocytes for each substrate, pre-loaded in parallel to those used for efflux *trans*-stimulation. Calculations were made assuming a cytosolic volume of 500 nl/oocyte (see above).

### Oocyte data analysis and statistics

All oocyte data were analyzed using OriginPro (2015). All data displayed in figures represent the mean ± S.D. except where otherwise indicated. Unless uptake data from uninjected oocytes is included in figures, uptake in uninjected oocytes was subtracted from uptake in *Tg*ApiAT5-3-injected oocytes to give the ‘*Tg*ApiAT5-3-mediated uptake’. All data sets were analysed for Gaussian normalcy by first running a Shapiro-Wilk test prior to analysis and used only if passing the normalcy test at the P < 0.05 level. Multi-variant experiments with 3 or more experimental conditions were subjected to a one-way ANOVA with Dunnet’s post-hoc test and significance tested at the P < 0.05 level.

Timecourse analysis of uptake and oocyte retention data of L-Tyr in *Tg*ApiAT5-3-injected oocytes were fitter to 1^st^ order integrated rate equations:

$$S_{t}=S_{0}e^{-kt}$$ (1)

Or:

$$S_{t}=S_{max}{(1-e}^{-kt})$$ (2)

Eq 2 being the Box Lucas 1 model with zero offset [62], where S_t_, and S_0_, and are the amount of substrate (S) at variable time (t), or when t = 0, S_max_ is the vertical asymptote of substrate amount, and k is the 1^st^ order rate constant.

Steady-state kinetic data collected under initial rate conditions were fitted to both the Michaelis-Menten equation:

$$V=\frac{V_{max}∙[S]}{K_{0.5}+[S]}$$ (3)

And a Scatchard linear regression equation:

$$\frac{V}{\left[S\right]}=\frac{V_{max}}{K_{0.5}}-\frac{V}{K_{0.5}}$$ (4)

In the Scatchard regression, the apparent Michaelis constant (*K*_0.5_) is derived from the slope (1/−*K*_0.5_) and the maximal rate (*V*_*max*_) from the ordinate intercept (*V*_*max*_/*K*_0.5_).

All curve fittings were evaluated using adjusted R^2^ values as indicated in the text and figure legends. All non-linear fitting was conducted using the Levenburg-Marquardt algorithm, with iteration numbers varying from 4 to 11 before convergence was attained.

### Amino acid uptake assays in *T*. *gondii* parasites

Amino acid uptake assays were carried out as described previously [9], with slight modifications. Briefly, extracellular parasites were washed twice in Dulbecco’s PBS pH 7.4 (Sigma) supplemented with 10 mM glucose (PBS-glucose). Parasites were incubated in PBS-glucose containing radiolabelled amino acids, and 200 μl aliquots removed at regular time points. Parasite samples were centrifuged through an oil mix to separate parasites from unincorporated radiolabel as described previously [9]. L-Arg uptake was measured by incubation in 0.1 μCi/ml [^14^C]Arg and 100 μM L-Arg, L-Tyr uptake was measured by incubation in 0.25 μCi/ml [^14^C]Tyr and 60 μM L-Tyr, and L-Phe uptake was measured by incubation in 0.1 μCi/ml [^14^C]Phe and 15 μM L-Phe. The timecourses of radiolabel uptake in each amino acid tested were fitted by a single exponential function and the initial rate of transport was estimated from the initial slope of the fitted line.

### Ethics statement

All animal research was conducted in accordance with the National Health and Medical Research Council’s Australian Code for the Care and Use of Animals for Scientific Purposes, and the Australian Capital Territory Animal Welfare Act 1992. Mice were maintained and handled in accordance with protocols approved by the Australian National University Animal Experimentation Ethics Committee (protocol number A2016/42). Maintenance of *Xenopus laevis* and preparation of oocytes was approved by the Australian National University Animal Experimentation Ethics Committee (protocol number A2014/20).

## Supporting information

S1 Fig Multiple sequence alignment of ApiAT family proteins from apicomplexans. A multiple sequence alignment of the 66 ApiAT family proteins examined in this study. The alignment is presented as a “fingerprint”, where each residue is represented by a thin vertical line that has been shaded to represent the degree of conservation (as described previously; [65]). Residues with >70% identity in the ApiAT alignment are depicted in purple, residues with 50–70% identity are depicted in cyan, residues where > 50% of residues have similar amino acids or where amino acids are similar to residues in the above identity groupings are depicted in magenta, non-conserved residues are depicted in gray, and gaps in the sequences are white. The approximate locations of the predicted transmembrane domains are represented by numbered bars.
(PDF)

S2 Fig Multiple sequence alignment of a selection of ApiAT family proteins from apicomplexans with human LAT3 and LAT4 proteins. A multiple sequence alignment of ApiAT-family proteins from apicomplexans (*Tg*ApiAT1, *Tg*ApiAT2, *Pb*ApiAT8, *Tg*ApiAT6-1 and *Tg*ApiAT5-3) and the human LAT3 and LAT4 proteins (*Hs*LAT3 and *Hs*LAT4). Residues with >70% sequence identity are shaded in black and residues with >70% sequence similarity are shaded in gray. The red box highlights the MFS signature sequence.
(TIF)

S3 Fig Genetic modifications to introduce HA tags into the native loci of *Tg*ApiAT genes in *T*. *gondii*. (A) Single cross-over recombination approach, where a vector containing a homologous flanking sequence to the target gene, in addition to a chloramphenicol resistance marker (ChlR), is introduced into *T*. *gondii* parasites. Single cross-over recombination results in the insertion of a HA tag into the 3’ region of the open reading frame of the target gene. The approximate position of the primers used to screen *Tg*ApiAT7-1-HA clones are depicted. (B) CRISPR/Cas9 genome editing approach, where a guide RNA (gRNA) is designed to target a region near the stop codon of the target gene. When co-expressed with Cas9-GFP, the gRNA mediates a double-stranded break in the parasite genome near the stop codon of the target gene. The gRNA/Cas9-GFP vector is co-transfected with a donor DNA product that contains a HA tag flanked on either side with 50 bp of sequence homologous to regions immediately up and downstream of the stop codon in the target gene. Homologous repair results in introduction of the HA tag into the 3’ region of the open reading frame of the target gene. (C) PCR screen to test for integration of the HA tag into the *Tg*ApiAT7-1 locus. The presence of a 2.5 kb band that is absent from the wild type (WT) control indicates that clones 2–5 have successfully integrated the HA tag.
(TIF)

S4 Fig Localization of *Tg*ApiAT5-3-HA in relation to the IMC. (A-B) Immunofluorescence assays to determine the localisation of *Tg*ApiAT5-3-HA (anti-HA; green in merge) in relation to the inner membrane complex (anti-IMC; red in merge). Arrows indicate regions at the basal (A) and apical (B) ends of the parasite where *Tg*ApiAT5-3-HA is present and the IMC marker is absent. Both scale bars are 2 μm.
(TIF)

S5 Fig Comparisons of the growth of wild type and *Tg*ApiAT mutant parasites in complete vs minimal amino acid medium. (A-F) Plaque assays depicting growth of parental (RHΔ*hxgprt*, TATi or TATi tdTomato) and *Tg*ApiAT1 mutant parasites in complete medium (DMEM; top) or minimal amino acid medium (MAAM; bottom). 150 parasites were added to wells of a 6-well plate and cultured for 9 days. (A) WT (RHΔ*hxpgrt*) and *apiAT1*^*Δ54–534*^ parasites. (B) WT (TATi/Tomato) and *apiAT2*^*Δ138–588*^ parasites. (C) WT (TATi) and *apiAT3* sub-family mutants. (D) WT (TATi/Tomato) and *apiAT5* sub-family mutants. (E) WT (TATi/Tomato) and *apiAT6* sub-family mutants. (F) *apiAT7* sub-family mutants. Note that the TATi/Tomato strain served as WT strain for the *apiAT2*, *apiAT5*, *apiAT6*, and *apiAT7* sub-family mutants, and identical images of the TATi/Tomato plaque assay in DMEM and MAAM are shown in B, D and E to facilitate interpretation of the data. The DMEM images are from the same experiment as depicted in Fig 3. All images are from the same experiment, and are representative of three independent experiments.
(TIF)

S6 Fig Characterisation of *Tg*ApiAT5-3 expressed in oocytes. (A) Western blot with anti-HA antibodies on whole membrane preparations (WMP) and surface biotinylated proteins (SB) in oocytes expressing HA-tagged *Tg*ApiAT5-3 (5–3) or oocytes that were uninjected (u.i.). (B) Efflux and retention of preloaded [^14^C]Tyr in uninjected oocytes. Uninjected oocytes were preloaded by incubation in 1 mM [^14^C]Tyr for 72 hr as described in methods. Subsequent efflux (filled shapes) and retention (open shapes) of the preloaded labelled substrate was measured over the timecourse indicated in the presence in the extracellular buffer of 2.5 mM L-Tyr (squares) or in the absence of L-Tyr (circles). Data show the mean efflux and retention in 5 oocytes from a single experiment ± standard deviation, and are representative of 3 independent experiments. (C) *Tg*ApiAT5-3-expressing oocytes (black) or uninjected oocytes (white) were preloaded via incubation in 2.5 mM L-tyrosine for 32 or 72 hr, respectively, as described in methods. Subsequent uptake of 1 mM L-Tyr containing 0.5 μCi/ml [^14^C]Tyr was measured in buffer where the ions were replaced as indicated. For Na^+^ replacement conditions, the replacement cation is written at the top of the respective histogram. Data show the mean uptake in 10 oocytes from a single experiment ± standard deviation, and are representative of 3 independent experiments. Uptake in *Tg*ApiAT5-3-expressing oocytes was not significantly different in any condition tested (P > 0.05, one-way ANOVA, Dunnet’s post-hoc test). (D) *Tg*ApiAT5-3-expressing oocytes were impaled and recorded using a two-voltage clamp amplifier configuration 4–5 days post-cRNA injection. Oocytes were continuously perfused with gravity-fed ND96 buffer (pH 7.4) until otherwise indicated by the arrows in the current tracings. Top: representative current trace upon the addition of 1 mM L-Tyr at E_m_ = −50 mV or 0 mV. Bottom: representative current trace upon the change to pH 9.0 and incubation in 1 mM L-Tyr. No baselines were corrected in either tracing. Data are representative of 12 replicates.
(TIF)

S7 Fig Substrate specificity of *Tg*ApiAT5-3. (A) Uptake of 500 μM L-Tyr containing 0.5 μCi/ml [^14^C] Tyr was measured in *Tg*ApiAT5-3-expressing oocytes (black) or uninjected oocytes (white) over 10 mins in presence of 500 μM unlabelled L-amino acids. Data show the mean uptake in 10 oocytes from a single experiment ± standard deviation, and are representative of 2 independent experiments (*, P < 0.05, one-way ANOVA, Dunnet’s post-hoc test. Where significance values are not shown, the differences are not significant, P > 0.05). (B) Uptake of L-Tyr in *Tg*ApiAT5-3-expressing oocytes (black) or uninjected oocytes (white) over 10 mins, where oocytes were pre-injected with uptake buffer (ND96), preloaded with 2.5 mM L-Tyr, or pre-injected with various substrate mixes, including L-amino acids (L-AA1-3), amino acid derivatives (AA derivatives 1–3), D-amino acids (D-AA), nucleosides, nitrogen bases, or sugars (see S4 Table for compositions). Data show the mean uptake in 8–10 oocytes from a single experiment ± standard deviation, and are representative of 3 independent experiments (*, P < 0.05, one-way ANOVA, Dunnet’s post-hoc test. Where significance values are not shown, the differences are not significant, P > 0.05). (C) Uptake of various [^14^C]Amino acids (at 1 mM final substrate concentration) was measured in *Tg*ApiAT5-3-expressing oocytes (black) or uninjected oocytes (white) over 10 mins. Data show the mean uptake in 10 oocytes from a single experiment ± standard deviation, and are representative of 3 independent experiments (*, P < 0.05, one-way ANOVA, Dunnet’s post-hoc test, for differences between *Tg*ApiAT5-3-injected and uninjected oocytes for the same substrate. Where significance values are not shown, the differences are not significant, P > 0.05).
(TIF)

S8 Fig Timecourses for the uptake of [^14^C]Tyr, [^14^C]Phe and [^14^C]Arg in *T*. *gondii*. Uptake of [^14^C]Tyr (A), [^14^C]Phe (B), and [^14^C]Arg (C) in WT, *apiAT5-3*^*Δ188–504*^, and *apiAT5-3*^*Δ188-504*^/c*Tg*ApiAT5-3 strain parasites. Uptake was measured in PBS-glucose containing either 60 μM unlabelled L-Tyr and 0.1 μCi/ml [^14^C]Tyr (A), 15 μM unlabelled L-Phe and 0.1 μCi/ml [^14^C]Phe (B), or 100 μM unlabelled L-Arg and 0.1 μCi/ml [^14^C]Arg (C). Data points represent the mean ± SEM from three independent experiments. Lines represent fitted single-order exponential curves, from which the initial rates were calculated and depicted in Fig 7.
(TIF)

S9 Fig Complementation of *apiAT5-3*^*Δ188–504*^ strain parasites with a constitutive copy of *Tg*ApiAT5-3 restores parasite growth in DMEM. Plaque assays depicting growth of WT parasites (top), *apiAT5-3*^*Δ188–504*^ (middle), and *apiAT5-3*^*Δ188-504*^/c*Tg*ApiAT5-3 parasites (bottom). 500 parasites were added to 25 cm^2^ tissue culture flasks and cultured in DMEM (left) or DMEM containing 2.5 mM L-Tyr (right) for 8 days before fixation and staining with crystal violet. Data are representative of three independent experiments.
(TIF)

S10 Fig *T*. *gondii* parasites are auxotrophic for all three proteinogenic aromatic amino acids. Fluorescence growth assays measuring the growth of WT (black), *apiAT5-3*^*Δ188–504*^ (red), and *apiAT5-3*^*Δ188-504*^/c*Tg*ApiAT5-3 (blue) parasites in DMEM containing the indicated concentrations of L-Tyr (A), L-Phe (B) and L-Trp (C). The growth of parasites is expressed as a percentage of the optimal concentration for each amino acid tested in each parasite strain, and was measured at mid-log phase for this optimal concentration (5 days post-inoculation). Parasite growth was determined using the same amino acid concentrations used in Fig 8, but included a 0 mM concentration (which was not possible to depict in Fig 8 because of the log scale on the x axes). For simplicity, only the following amino acid concentrations are depicted in this figure: 0 mM, 0.423 mM and 2.5 mM L-Tyr (A), 0 mM, 0.32 mM and 10 mM L-Phe (B), and 0 mM, 0.063 mM and 1 mM L-Trp (C). The data for 0.423 mM L-Tyr (the normal DMEM concentration of L-Tyr) were interpolated from curve fitting while 0.32 mM L-Phe and 0.063 mM L-Trp (the nearest tested concentrations to those present in DMEM) were experimental data points. Data represent the mean ± SEM from three independent experiments (**** P < 0.0001; two-way ANOVA with Tukey’s multiple comparison test).
(TIF)

S1 Table Gene identification numbers of ApiAT proteins identified in this study, and predicted molecular mass of *T*. *gondii* ApiAT proteins. (DOCX)

S2 Table Summary of the mutations generated through CRISPR/Cas9-based genome editing of targeted *Tg*ApiAT genes in *T*. *gondii*. (DOCX)

S3 Table The amino acid compositions of Roswell Park Memorial Institute 1640 medium (RPMI), Dulbecco’s Modified Eagle’s Medium (DMEM), and Minimal Amino Acid Medium (MAAM) used in this study. (DOCX)

S4 Table Composition and concentrations of metabolite mixes used in S7B Fig. (DOCX)

S5 Table Forward primers used to generate CRISPR/Cas9 vectors targeting *Tg*ApiAT genes for gene disruption. (DOCX)

S6 Table Forward primers used to generate CRISPR/Cas9 vectors targeting *Tg*ApiAT genes for 3’ replacement with an HA epitope tag. (DOCX)

S7 Table Primers used to amplify 3x HA tags containing 50 bp flanking sequences to the target gene, as well as sequence of the gBlock oligonucleotide encoding the 3x HA tag used as template. (DOCX)

S8 Table Primers used to amplify 3’ flanks from target *Tg*ApiAT genes. Flanking sequences were ligated into the pgCH vector (unless indicated) and linearized with the indicated restriction enzyme.
(DOCX)
