# Supplementary figures and images for: The tyrosine transporter of Toxoplasma gondii is a member of the newly defined apicomplexan amino acid transporter (ApiAT) family
Source: PLoS Pathog. 2019 Feb 11;15(2):e1007577. doi: 10.1371/journal.ppat.1007577 (PMC6386423; doi:10.1371/journal.ppat.1007577)

*N-terminal extension (internal)*

1

2

3

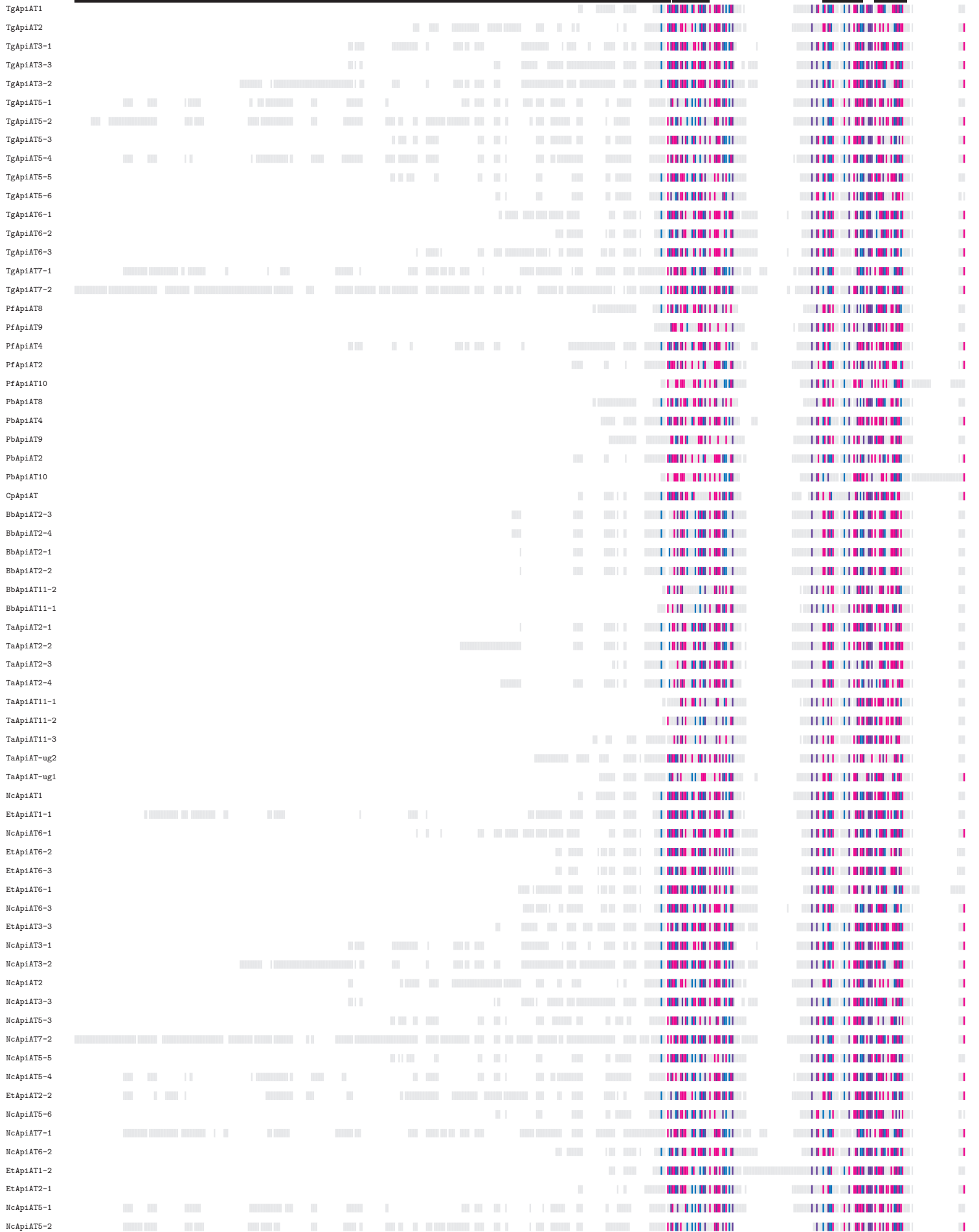

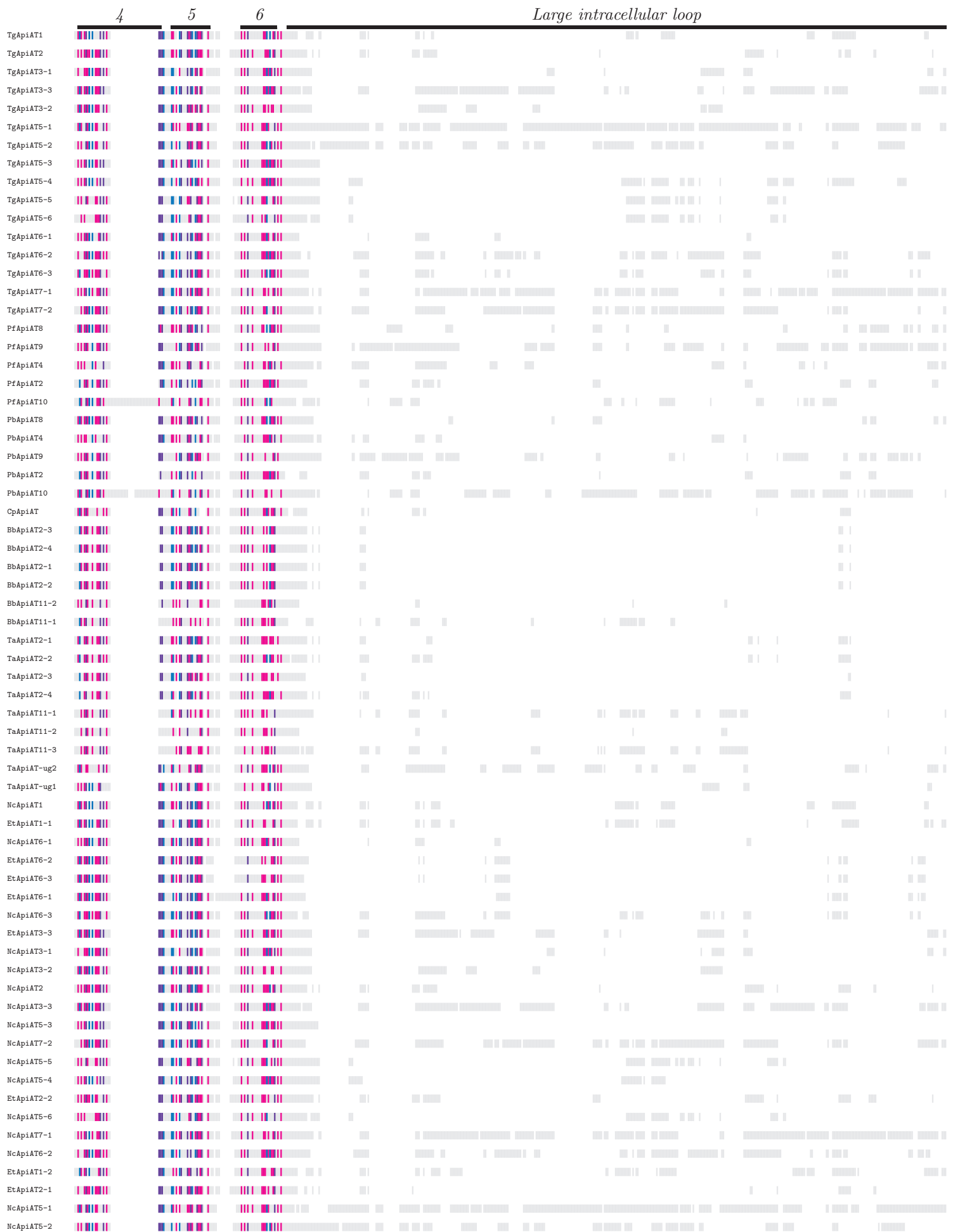

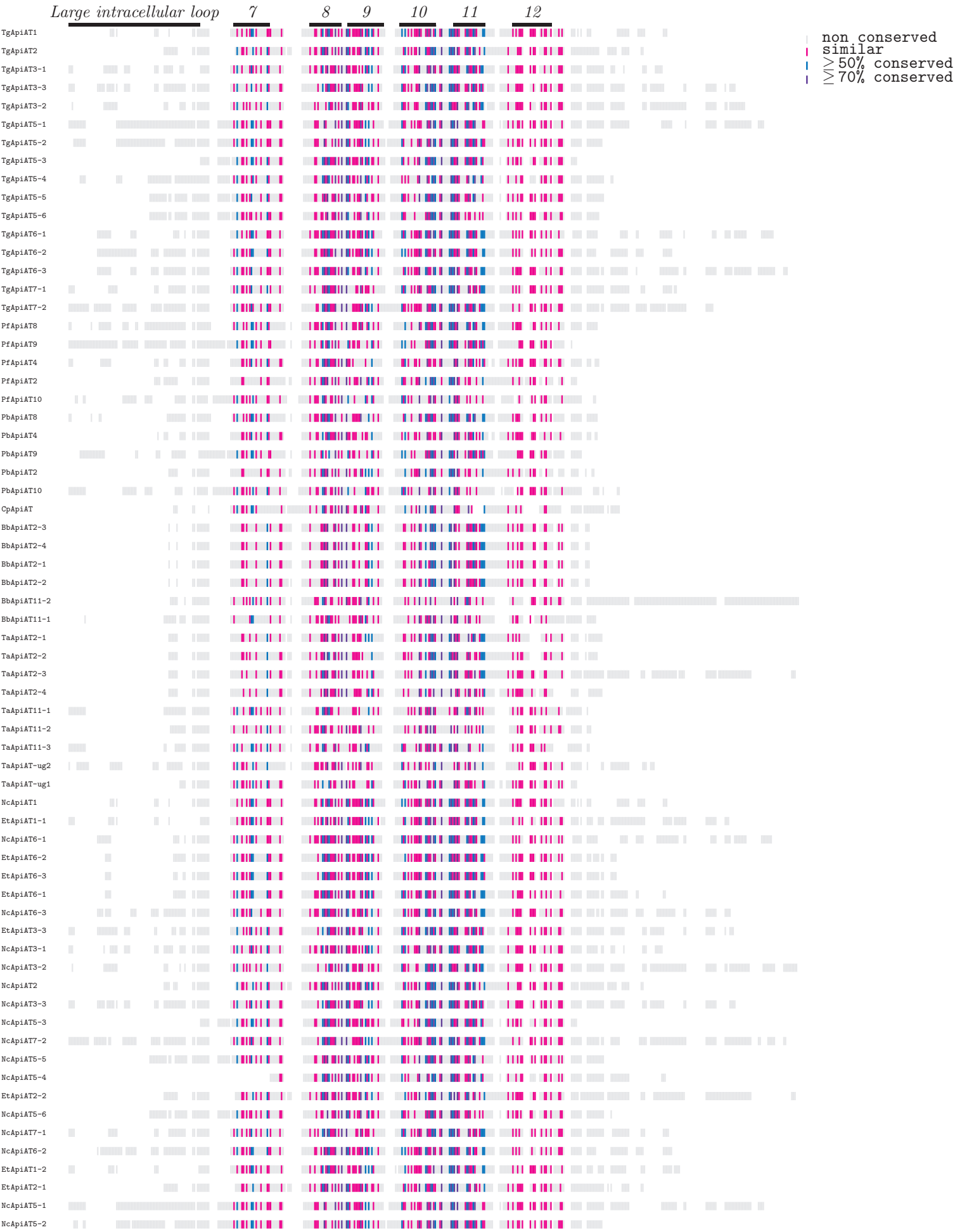

Supplement: S1 Fig — A multiple sequence alignment of the 66 ApiAT family proteins examined in this study. The alignment is presented as a “fingerprint”, where each residue is represented by a thin vertical line that has been shaded to represent the degree of conservation (as described previously; [65]). Residues with >70% identity in the ApiAT alignment are depicted in purple, residues with 50–70% identity are depicted in cyan, residues where > 50% of residues have similar amino acids or where amino acids are similar to residues in the above identity groupings are depicted in magenta, non-conserved residues are depicted in gray, and gaps in the sequences are white. The approximate locations of the predicted transmembrane domains are represented by numbered bars. (PDF) [file ppat.1007577.s001.pdf]

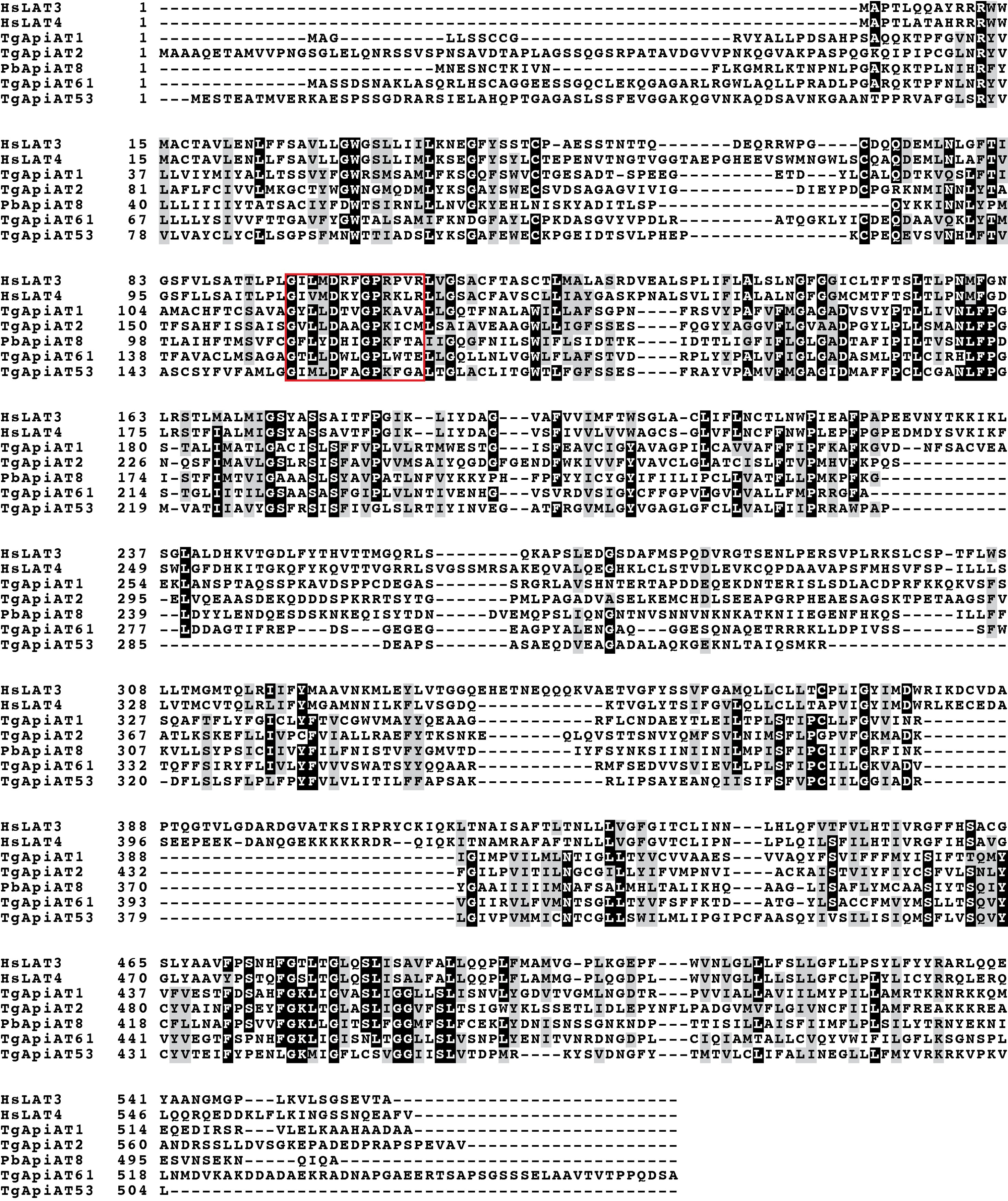

Supplement: S2 Fig — A multiple sequence alignment of ApiAT-family proteins from apicomplexans (TgApiAT1, TgApiAT2, PbApiAT8, TgApiAT6-1 and TgApiAT5-3) and the human LAT3 and LAT4 proteins (HsLAT3 and HsLAT4). Residues with >70% sequence identity are shaded in black and residues with >70% sequence similarity are shaded in gray. The red box highlights the MFS signature sequence. (TIF) [file ppat.1007577.s002.tif]

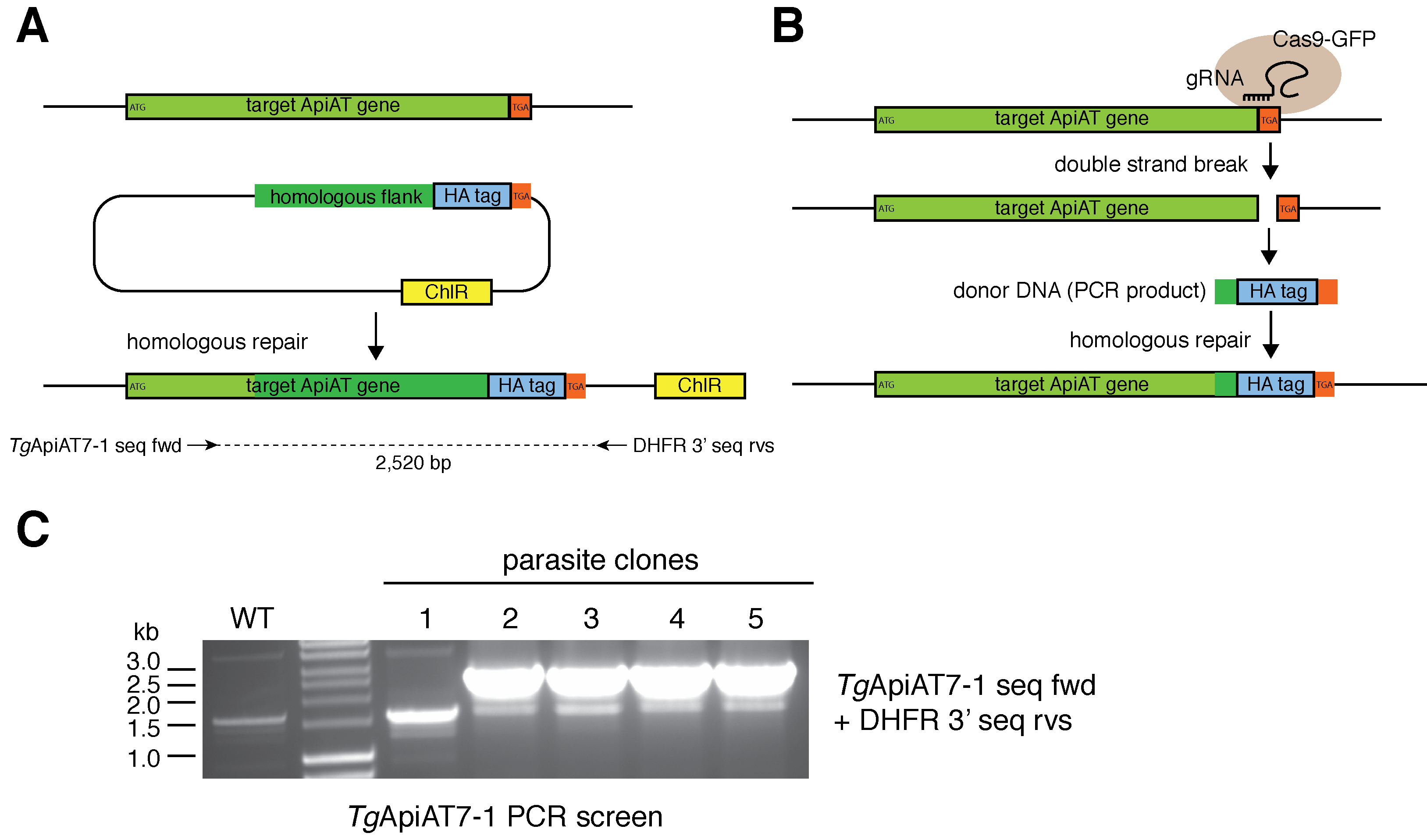

Supplement: S3 Fig — (A) Single cross-over recombination approach, where a vector containing a homologous flanking sequence to the target gene, in addition to a chloramphenicol resistance marker (ChlR), is introduced into T. gondii parasites. Single cross-over recombination results in the insertion of a HA tag into the 3’ region of the open reading frame of the target gene. The approximate position of the primers used to screen TgApiAT7-1-HA clones are depicted. (B) CRISPR/Cas9 genome editing approach, where a guide RNA (gRNA) is designed to target a region near the stop codon of the target gene. When co-expressed with Cas9-GFP, the gRNA mediates a double-stranded break in the parasite genome near the stop codon of the target gene. The gRNA/Cas9-GFP vector is co-transfected with a donor DNA product that contains a HA tag flanked on either side with 50 bp of sequence homologous to regions immediately up and downstream of the stop codon in the target gene. Homologous repair results in introduction of the HA tag into the 3’ region of the open reading frame of the target gene. (C) PCR screen to test for integration of the HA tag into the TgApiAT7-1 locus. The presence of a 2.5 kb band that is absent from the wild type (WT) control indicates that clones 2–5 have successfully integrated the HA tag. (TIF) [file ppat.1007577.s003.tif]

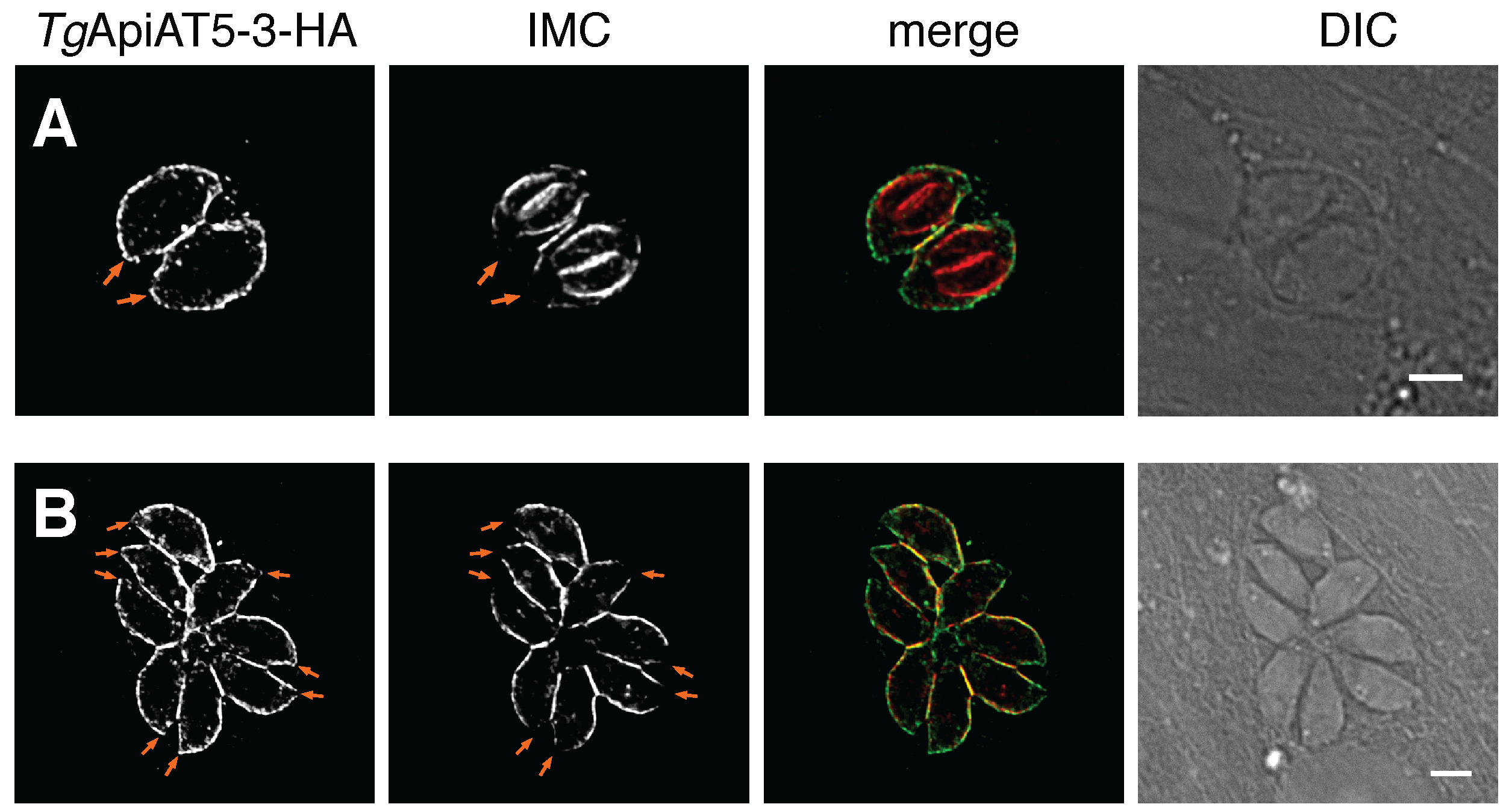

Supplement: S4 Fig — (A-B) Immunofluorescence assays to determine the localisation of TgApiAT5-3-HA (anti-HA; green in merge) in relation to the inner membrane complex (anti-IMC; red in merge). Arrows indicate regions at the basal (A) and apical (B) ends of the parasite where TgApiAT5-3-HA is present and the IMC marker is absent. Both scale bars are 2 μm. (TIF) [file ppat.1007577.s004.tif]

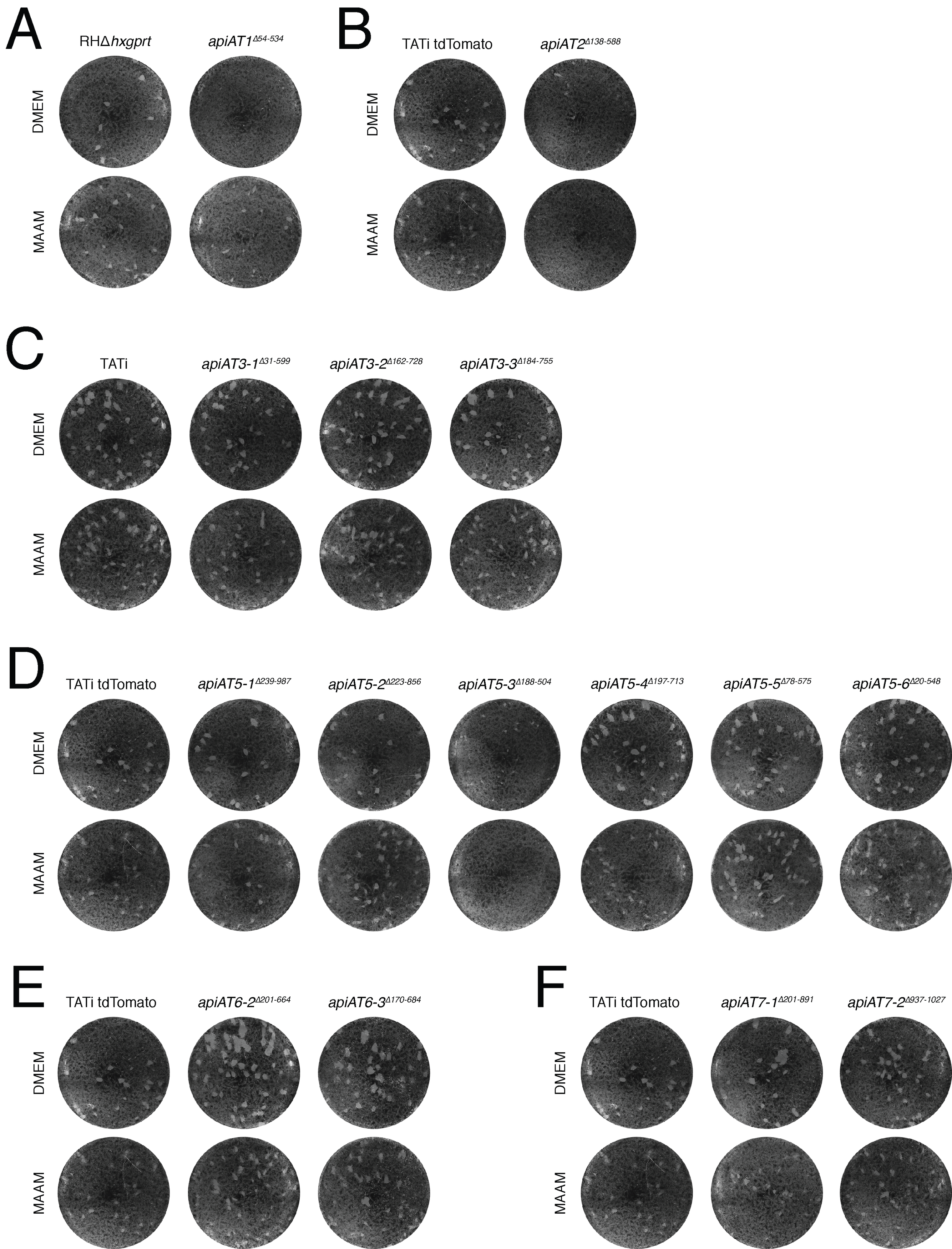

Supplement: S5 Fig — (A-F) Plaque assays depicting growth of parental (RHΔhxgprt, TATi or TATi tdTomato) and TgApiAT1 mutant parasites in complete medium (DMEM; top) or minimal amino acid medium (MAAM; bottom). 150 parasites were added to wells of a 6-well plate and cultured for 9 days. (A) WT (RHΔhxpgrt) and apiAT1Δ54–534 parasites. (B) WT (TATi/Tomato) and apiAT2Δ138–588 parasites. (C) WT (TATi) and apiAT3 sub-family mutants. (D) WT (TATi/Tomato) and apiAT5 sub-family mutants. (E) WT (TATi/Tomato) and apiAT6 sub-family mutants. (F) apiAT7 sub-family mutants. Note that the TATi/Tomato strain served as WT strain for the apiAT2, apiAT5, apiAT6, and apiAT7 sub-family mutants, and identical images of the TATi/Tomato plaque assay in DMEM and MAAM are shown in B, D and E to facilitate interpretation of the data. The DMEM images are from the same experiment as depicted in Fig 3. All images are from the same experiment, and are representative of three independent experiments. (TIF) [file ppat.1007577.s005.tif]

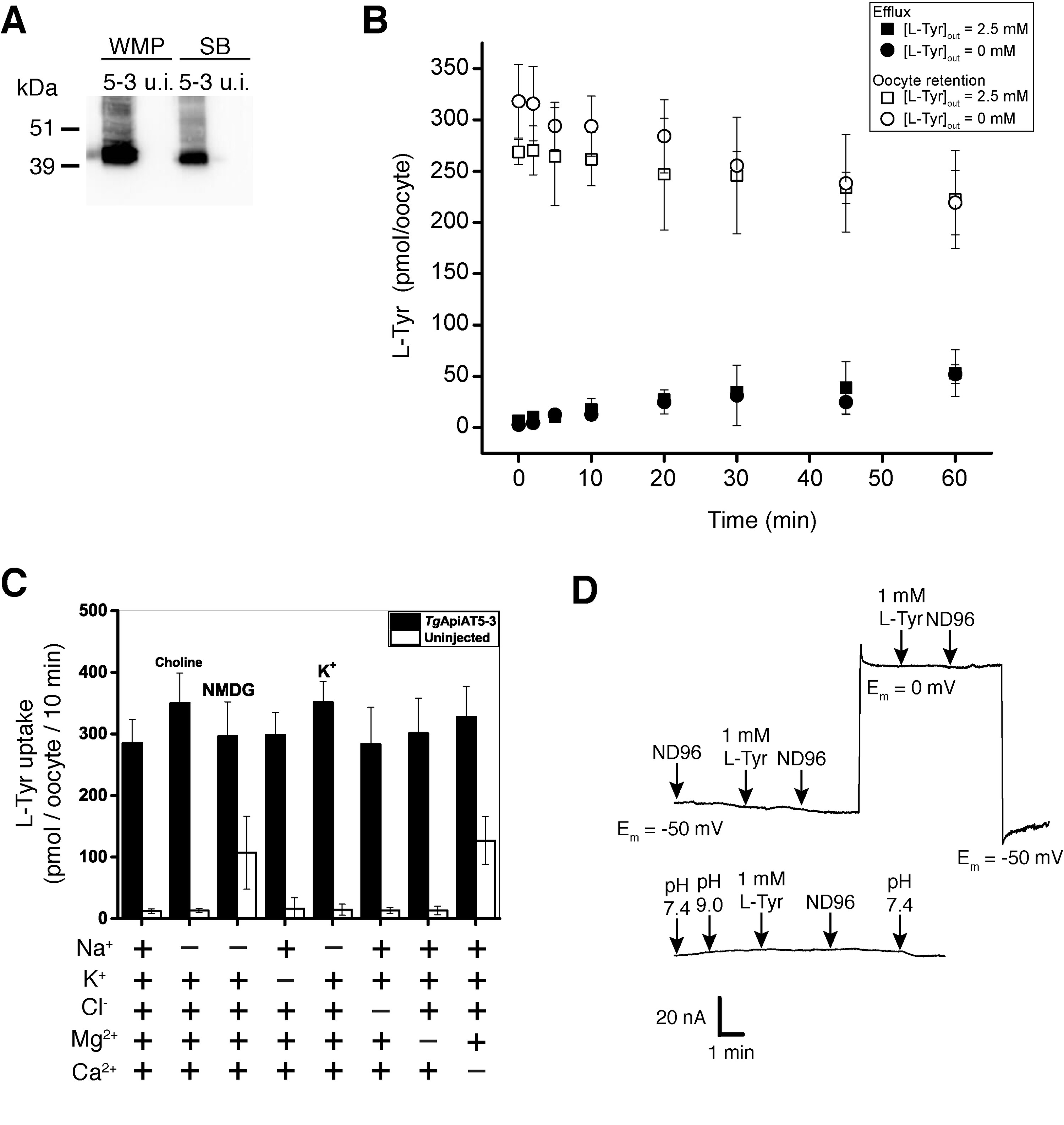

Supplement: S6 Fig — (A) Western blot with anti-HA antibodies on whole membrane preparations (WMP) and surface biotinylated proteins (SB) in oocytes expressing HA-tagged TgApiAT5-3 (5–3) or oocytes that were uninjected (u.i.). (B) Efflux and retention of preloaded [14C]Tyr in uninjected oocytes. Uninjected oocytes were preloaded by incubation in 1 mM [14C]Tyr for 72 hr as described in methods. Subsequent efflux (filled shapes) and retention (open shapes) of the preloaded labelled substrate was measured over the timecourse indicated in the presence in the extracellular buffer of 2.5 mM L-Tyr (squares) or in the absence of L-Tyr (circles). Data show the mean efflux and retention in 5 oocytes from a single experiment ± standard deviation, and are representative of 3 independent experiments. (C) TgApiAT5-3-expressing oocytes (black) or uninjected oocytes (white) were preloaded via incubation in 2.5 mM L-tyrosine for 32 or 72 hr, respectively, as described in methods. Subsequent uptake of 1 mM L-Tyr containing 0.5 μCi/ml [14C]Tyr was measured in buffer where the ions were replaced as indicated. For Na+ replacement conditions, the replacement cation is written at the top of the respective histogram. Data show the mean uptake in 10 oocytes from a single experiment ± standard deviation, and are representative of 3 independent experiments. Uptake in TgApiAT5-3-expressing oocytes was not significantly different in any condition tested (P > 0.05, one-way ANOVA, Dunnet’s post-hoc test). (D) TgApiAT5-3-expressing oocytes were impaled and recorded using a two-voltage clamp amplifier configuration 4–5 days post-cRNA injection. Oocytes were continuously perfused with gravity-fed ND96 buffer (pH 7.4) until otherwise indicated by the arrows in the current tracings. Top: representative current trace upon the addition of 1 mM L-Tyr at Em = −50 mV or 0 mV. Bottom: representative current trace upon the change to pH 9.0 and incubation in 1 mM L-Tyr. No baselines were corrected in either tracing. Data are repr [file ppat.1007577.s006.tif]

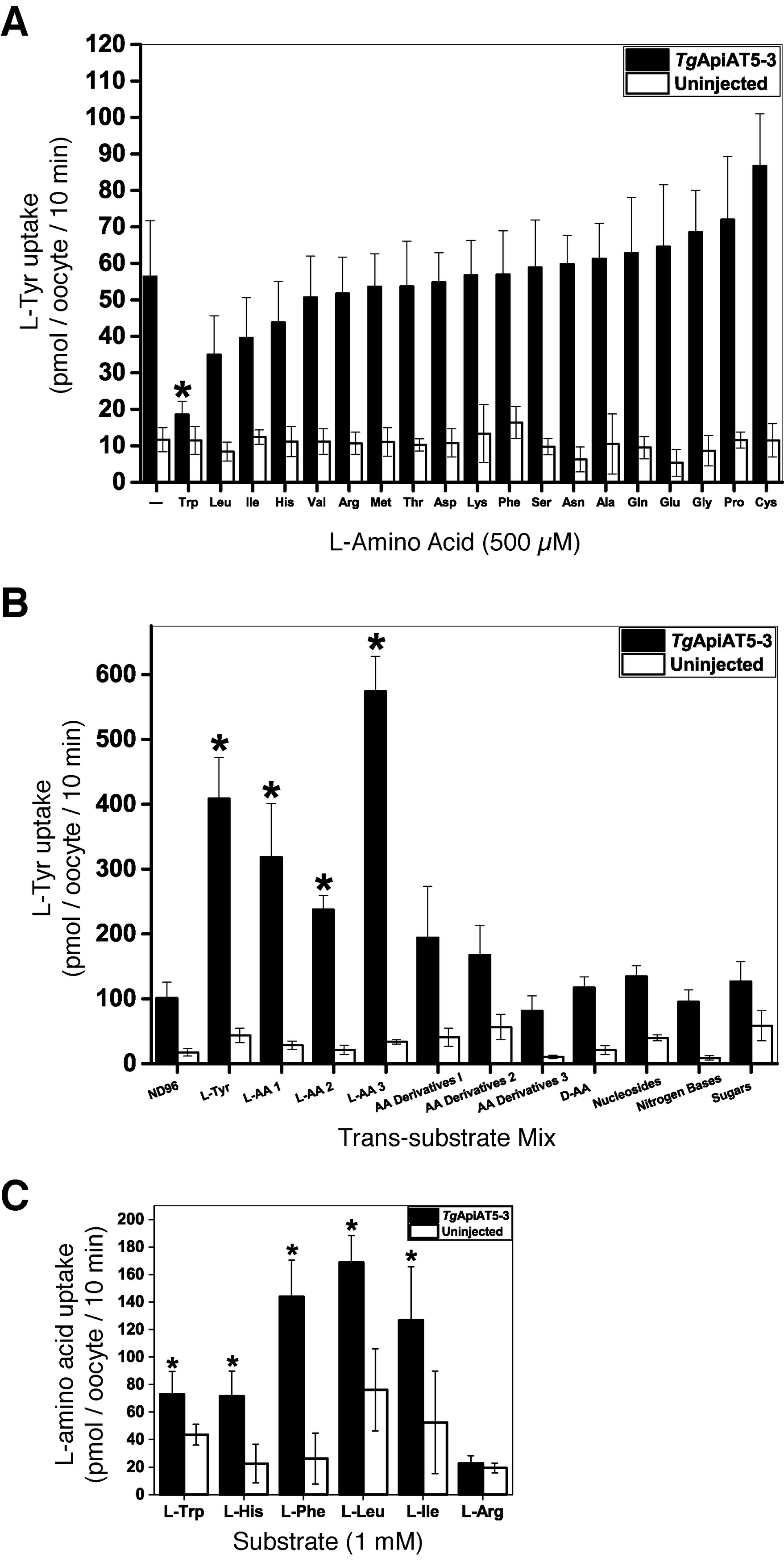

Supplement: S7 Fig — (A) Uptake of 500 μM L-Tyr containing 0.5 μCi/ml [14C] Tyr was measured in TgApiAT5-3-expressing oocytes (black) or uninjected oocytes (white) over 10 mins in presence of 500 μM unlabelled L-amino acids. Data show the mean uptake in 10 oocytes from a single experiment ± standard deviation, and are representative of 2 independent experiments (*, P < 0.05, one-way ANOVA, Dunnet’s post-hoc test. Where significance values are not shown, the differences are not significant, P > 0.05). (B) Uptake of L-Tyr in TgApiAT5-3-expressing oocytes (black) or uninjected oocytes (white) over 10 mins, where oocytes were pre-injected with uptake buffer (ND96), preloaded with 2.5 mM L-Tyr, or pre-injected with various substrate mixes, including L-amino acids (L-AA1-3), amino acid derivatives (AA derivatives 1–3), D-amino acids (D-AA), nucleosides, nitrogen bases, or sugars (see S4 Table for compositions). Data show the mean uptake in 8–10 oocytes from a single experiment ± standard deviation, and are representative of 3 independent experiments (*, P < 0.05, one-way ANOVA, Dunnet’s post-hoc test. Where significance values are not shown, the differences are not significant, P > 0.05). (C) Uptake of various [14C]Amino acids (at 1 mM final substrate concentration) was measured in TgApiAT5-3-expressing oocytes (black) or uninjected oocytes (white) over 10 mins. Data show the mean uptake in 10 oocytes from a single experiment ± standard deviation, and are representative of 3 independent experiments (*, P < 0.05, one-way ANOVA, Dunnet’s post-hoc test, for differences between TgApiAT5-3-injected and uninjected oocytes for the same substrate. Where significance values are not shown, the differences are not significant, P > 0.05). (TIF) [file ppat.1007577.s007.tif]

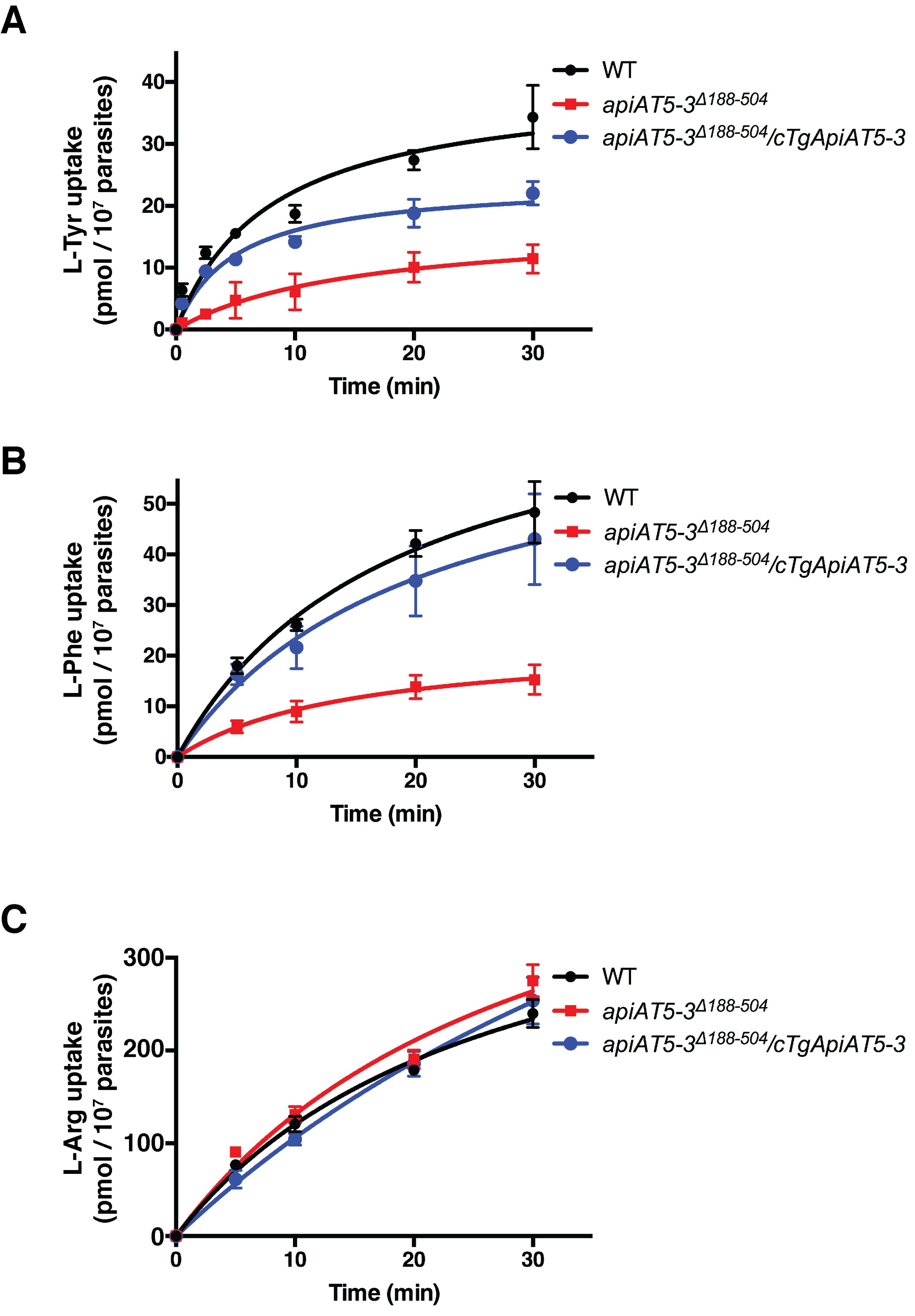

Supplement: S8 Fig — Uptake of [14C]Tyr (A), [14C]Phe (B), and [14C]Arg (C) in WT, apiAT5-3Δ188–504, and apiAT5-3Δ188-504/cTgApiAT5-3 strain parasites. Uptake was measured in PBS-glucose containing either 60 μM unlabelled L-Tyr and 0.1 μCi/ml [14C]Tyr (A), 15 μM unlabelled L-Phe and 0.1 μCi/ml [14C]Phe (B), or 100 μM unlabelled L-Arg and 0.1 μCi/ml [14C]Arg (C). Data points represent the mean ± SEM from three independent experiments. Lines represent fitted single-order exponential curves, from which the initial rates were calculated and depicted in Fig 7. (TIF) [file ppat.1007577.s008.tif]

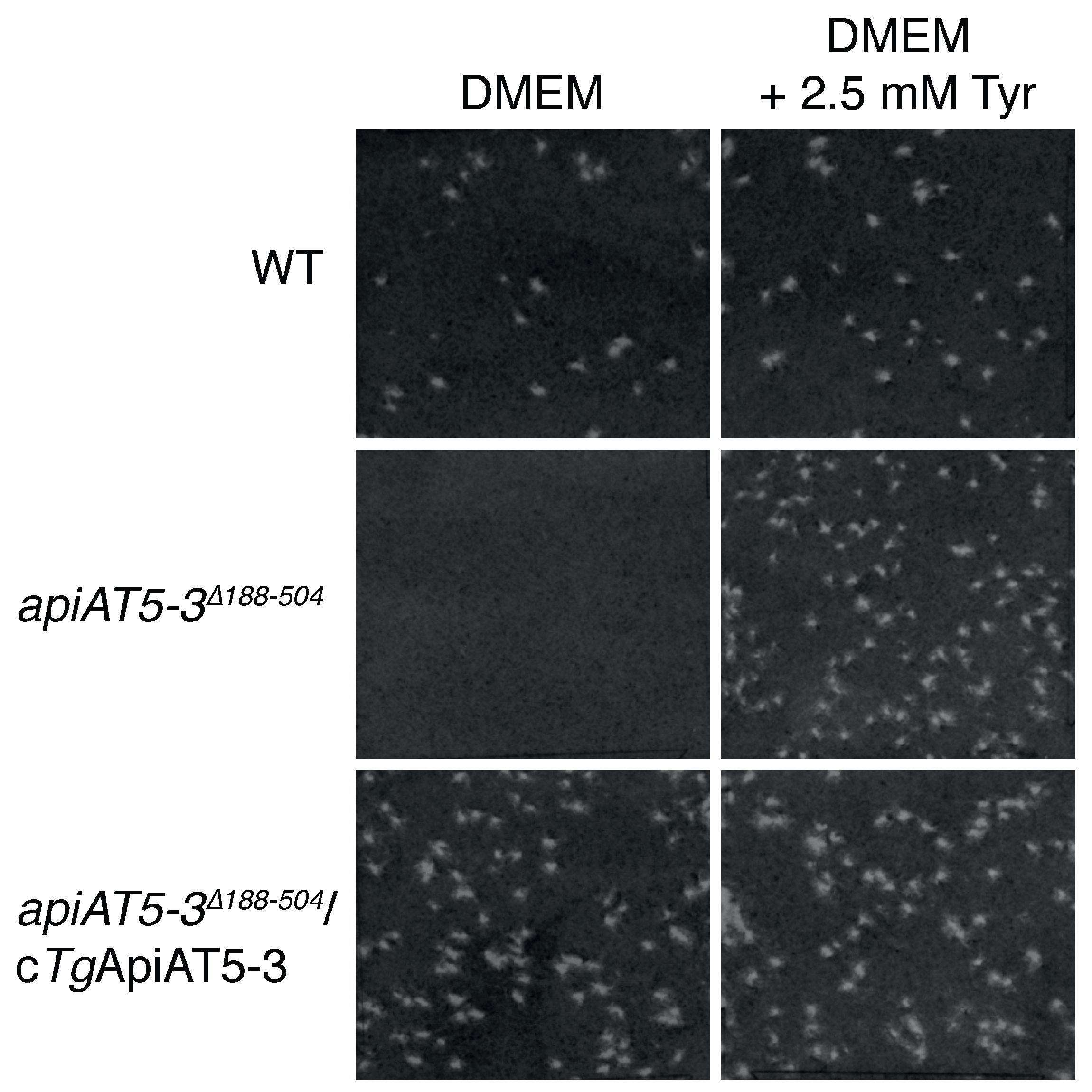

Supplement: S9 Fig — Plaque assays depicting growth of WT parasites (top), apiAT5-3Δ188–504 (middle), and apiAT5-3Δ188-504/cTgApiAT5-3 parasites (bottom). 500 parasites were added to 25 cm2 tissue culture flasks and cultured in DMEM (left) or DMEM containing 2.5 mM L-Tyr (right) for 8 days before fixation and staining with crystal violet. Data are representative of three independent experiments. (TIF) [file ppat.1007577.s009.tif]

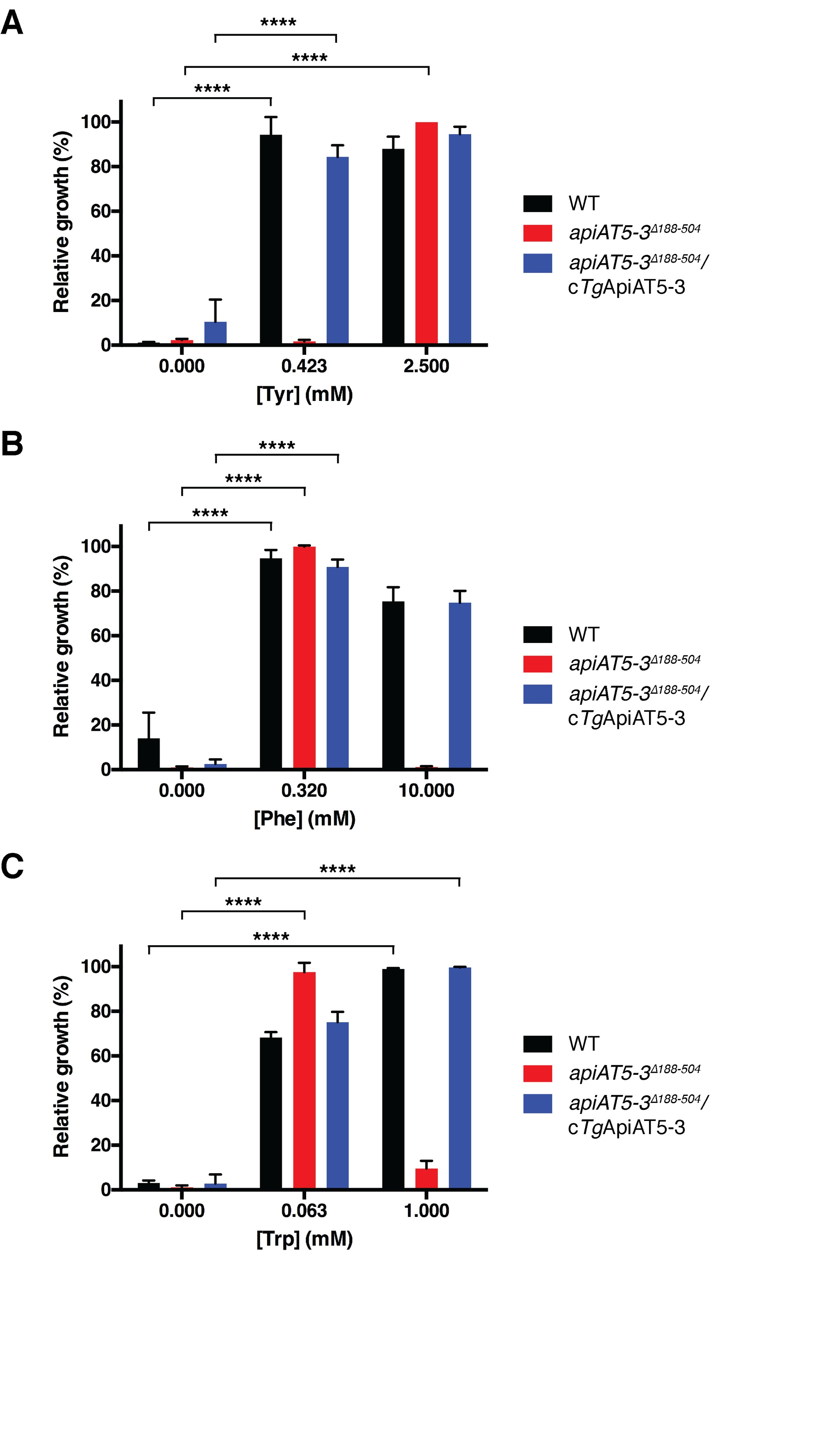

Supplement: S10 Fig — Fluorescence growth assays measuring the growth of WT (black), apiAT5-3Δ188–504 (red), and apiAT5-3Δ188-504/cTgApiAT5-3 (blue) parasites in DMEM containing the indicated concentrations of L-Tyr (A), L-Phe (B) and L-Trp (C). The growth of parasites is expressed as a percentage of the optimal concentration for each amino acid tested in each parasite strain, and was measured at mid-log phase for this optimal concentration (5 days post-inoculation). Parasite growth was determined using the same amino acid concentrations used in Fig 8, but included a 0 mM concentration (which was not possible to depict in Fig 8 because of the log scale on the x axes). For simplicity, only the following amino acid concentrations are depicted in this figure: 0 mM, 0.423 mM and 2.5 mM L-Tyr (A), 0 mM, 0.32 mM and 10 mM L-Phe (B), and 0 mM, 0.063 mM and 1 mM L-Trp (C). The data for 0.423 mM L-Tyr (the normal DMEM concentration of L-Tyr) were interpolated from curve fitting while 0.32 mM L-Phe and 0.063 mM L-Trp (the nearest tested concentrations to those present in DMEM) were experimental data points. Data represent the mean ± SEM from three independent experiments (**** P < 0.0001; two-way ANOVA with Tukey’s multiple comparison test). (TIF) [file ppat.1007577.s010.tif]
