# Supplementary material for: The tyrosine transporter of Toxoplasma gondii is a member of the newly defined apicomplexan amino acid transporter (ApiAT) family
Source: PLoS Pathog. 2019 Feb 11;15(2):e1007577. doi: 10.1371/journal.ppat.1007577 (PMC6386423; doi:10.1371/journal.ppat.1007577)
Supplement: S1 Table — (DOCX) [file ppat.1007577.s011.docx]

**S1 Table.** Gene identification numbers of ApiAT proteins identified in this study, and predicted molecular mass of *T. gondii* ApiAT proteins.

| **Name** | **Species** | **Gene ID** | **Predicted mass (kDa)** | **Notes** |
| --- | --- | --- | --- | --- |
| TgApiAT1 | *T. gondii* | TGGT1_215490 | 58.2 | Previously known as *Tg*NPT1 ^(1)^. |
| TgApiAT2 | *T. gondii* | TGGT1_320020 | 63.0 |  |
| TgApiAT3-1 | *T. gondii* | TGGT1_318150 | 65.1 |  |
| TgApiAT3-2 | *T. gondii* | TGGT1_248420 | 79.0 |  |
| TgApiAT3-3 | *T. gondii* | TGGT1_220600 | 82.4 |  |
| TgApiAT5-1 | *T. gondii* | TGGT1_248610 | 104.5 |  |
| TgApiAT5-2 | *T. gondii* | TGGT1_205520 | 93.7 |  |
| TgApiAT5-3 | *T. gondii* | TGGT1_257530 | 54.5 |  |
| TgApiAT5-4 | *T. gondii* | TGGT1_216710 | 77.0 |  |
| TgApiAT5-5 | *T. gondii* | TGGT1_293420 | 62.0 |  |
| TgApiAT5-6 | *T. gondii* | TGME49_293425 | 60.2 |  |
| TgApiAT6-1 | *T. gondii* | TGGT1_240810 | 61.3 |  |
| TgApiAT6-2 | *T. gondii* | TGGT1_290860 | 72.5 |  |
| TgApiAT6-3 | *T. gondii* | TGGT1_249580 | 74.3 |  |
| TgApiAT7-1 | *T. gondii* | TGGT1_263230 | 96.8 |  |
| TgApiAT7-2 | *T. gondii* | TGGT1_263260 | 109.9 |  |
| NcApiAT1 | *N. caninum* | NCLIV_052410 |  |  |
| NcApiAT2 | *N. caninum* | NCLIV_010230 |  |  |
| NcApiAT3-1 | *N. caninum* | NCLIV_011330 |  |  |
| NcApiAT3-2 | *N. caninum* | NCLIV_064380 |  |  |
| NcApiAT3-3 | *N. caninum* | NCLIV_012660 |  |  |
| NcApiAT5-1 | *N. caninum* | NCLIV_064770 |  |  |
| NcApiAT5-2 | *N. caninum* | NCLIV_020170 |  |  |
| NcApiAT5-3 | *N. caninum* | NCLIV_029570 |  |  |
| NcApiAT5-4 | *N. caninum* | NCLIV_059570 |  |  |
| NcApiAT5-5 | *N. caninum* | NCLIV_000370 |  |  |
| NcApiAT5-6 | *N. caninum* | NCLIV_000380 |  |  |
| NcApiAT6-1 | *N. caninum* | NCLIV_016970 |  |  |
| NcApiAT6-2 | *N. caninum* | NCLIV_042730 |  |  |
| NcApiAT6-3 | *N. caninum* | NCLIV_065740 |  |  |
| NcApiAT7-1 | *N. caninum* | NCLIV_024690 |  |  |
| NcApiAT7-2 | *N. caninum* | NCLIV_024670 |  |  |
| EtApiAT1-1 | *E. tenella* | ETH_00020400 |  |  |
| EtApiAT1-2 | *E. tenella* | ETH_00023675 |  |  |
| EtApiAT2-1 | *E. tenella* | ETH_00027485 |  |  |
| EtApiAT2-2 | *E. tenella* | ETH_00009430 |  |  |
| EtApiAT3-3 | *E. tenella* | ETH_00027960 |  |  |
| EtApiAT6-1 | *E. tenella* | ETH_00008540 |  |  |
| EtApiAT6-2 | *E. tenella* | ETH_00008500 |  |  |
| EtApiAT6-3 | *E. tenella* | ETH_00008465 |  |  |
| PfApiAT8 | *P. falciparum* | PF3D7_0104800 |  |  |
| PfApiAT9 | *P. falciparum* | PF3D7_0104700 |  |  |
| PfApiAT2 | *P. falciparum* | PF3D7_0914700 |  |  |
| PfApiAT4 | *P. falciparum* | PF3D7_1129900 |  |  |
| PfApiAT10 | *P. falciparum* | PF3D7_0312500 |  |  |
| PbApiAT8 | *P. berghei* | PBANKA_020830 |  | Previously known as *Pb*NPT1^(2)^ |
| PbApiAT9 | *P. berghei* | PBANKA_020840 |  | Previously known as *Pb*MFR2^(3)^ |
| PbApiAT2 | *P. berghei* | PBANKA_081570 |  | Previously known as *Pb*MFR4^(3)^ |
| PbApiAT4 | *P. berghei* | PBANKA_091830 |  | Previously known as *Pb*MFR5^(3)^ |
| PbApiAT10 | *P. berghei* | PBANKA_041050 |  | Previously known as *Pb*MFR3^(3)^ |
| TaApiAT2-1 | *T. annulata* | TA05125 |  |  |
| TaApiAT2-2 | *T. annulata* | TA05160 |  |  |
| TaApiAT2-3 | *T. annulata* | TA05145 |  |  |
| TaApiAT2-4 | *T. annulata* | TA05155 |  |  |
| TaApiAT11-1 | *T. annulata* | TA14485 |  |  |
| TaApiAT11-3 | *T. annulata* | TA14490 |  |  |
| TaApiAT11-2 | *T. annulata* | TA14495 |  |  |
| TaApiAT-ug2 | *T. annulata* | TA21100 |  |  |
| TaApiAT-ug1 | *T. annulata* | TA12370 |  |  |
| BbApiAT2-1 | *B. bovis* | BBOV_II000930 |  |  |
| BbApiAT2-2 | *B. bovis* | BBOV_II004180 |  |  |
| BbApiAT2-3 | *B. bovis* | BBOV_I005070 |  |  |
| BbApiAT2-4 | *B. bovis* | BBOV_I005040 |  |  |
| BbApiAT11-1 | *B. bovis* | BBOV_II006990 |  |  |
| BbApiAT11-2 | *B. bovis* | BBOV_II007000 |  |  |
| CpApiAT | *C. parvum* | cgd2_1310 |  |  |

1. Rajendran E, Hapuarachchi SV, Miller CM, Fairweather SJ, Cai Y, Smith NC, et al. Cationic amino acid transporters play key roles in the survival and transmission of apicomplexan parasites. Nature Communications. 2017 Feb 16;8:14455.

2. Boisson B, Lacroix C, Bischoff E, Gueirard P, Bargieri DY, Franke-Fayard B, et al. The novel putative transporter NPT1 plays a critical role in early stages of *Plasmodium berghei* sexual development. Molecular Microbiology. 2011 Sep;81(5):1343-57.

3. Kenthirapalan S, Waters AP, Matuschewski K, Kooij TW. Functional profiles of orphan membrane transporters in the life cycle of the malaria parasite. Nature Communications. 2016;7:10519.
