# Supplementary material for: The tyrosine transporter of Toxoplasma gondii is a member of the newly defined apicomplexan amino acid transporter (ApiAT) family
Source: PLoS Pathog. 2019 Feb 11;15(2):e1007577. doi: 10.1371/journal.ppat.1007577 (PMC6386423; doi:10.1371/journal.ppat.1007577)
Supplement: S2 Table — (DOCX) [file ppat.1007577.s012.docx]

**S2 Table.** Summary of the mutations generated through CRISPR/Cas9-based genome editing of targeted *Tg*ApiAT genes in *T. gondii*.

| **ToxoDB gene ID** | **ApiAT** | **Parental Line** | **Mutation** | **Position of mutation (bp from start codon)** | **Resultant mutant strain** |
| --- | --- | --- | --- | --- | --- |
| TGGT1_215490 | *Tg*ApiAT1 | RH∆*hxgprt* | 254 bp insertion | 160 | *apiAT1^∆54-534^* |
| TGGT1_320020 | *Tg*ApiAT2 | TATi/Tomato | 1 bp insertion | 411 | *apiAT2^∆138-588^* |
| TGGT1_318150 | *Tg*ApiAT3-1 | TATi | 203 bp insertion | 91 | *apiAT3-1^∆31-599^* |
| TGGT1_248420 | *Tg*ApiAT3-2 | TATi | 1 bp insertion | 484 | *apiAT3-2^∆162-728^* |
| TGGT1_220600 | *Tg*ApiAT3-3 | TATi | 1 bp deletion | 552 | *apiAT3-3^∆184-755^* |
| TGGT1_248610 | *Tg*ApiAT5-1 | TATi/Tomato | 1 bp insertion | 716 | *apiAT5-1^∆239-987^* |
| TGGT1_205520 | *Tg*ApiAT5-2 | TATi/Tomato | 55bp insertion | 668 | *apiAT5-2^∆223-856^* |
| TGGT1_257530 | *Tg*ApiAT 5-3 | TATi/Tomato | 1bp insertion | 687 | *apiAT5-3^∆188-504^* |
| TGGT1_257530 | *Tg*ApiAT 5-3 | RH∆*hxgprt*/ Tomato | 1 bp deletion | 685 | *apiAT5-3^∆188-504^* |
| TGGT1_216710 | *Tg*ApiAT5-4 | TATi/Tomato | 41 bp insertion | 1096 | *apiAT5-4^∆197-713^* |
| TGGT1_293420 | *Tg*ApiAT5-5 | TATi/Tomato | 2 bp insertion | 232 | *apiAT5-5^∆78-575^* |
| TGME49_293425 | *Tg*ApiAT5-6 | TATi/Tomato | 1 bp deletion | 58 | *apiAT5-6^∆20-548^* |
| TGGT1_240810 | *Tg*ApiAT6-1 | TATi/Tomato | Two clones with a 3 bp insertion and one clone with a 3 bp deletion; no changes in reading frame | 236 | N/A |
| TGGT1_290860 | *Tg*ApiAT6-2 | TATi/Tomato | 1 bp deletion | 602 | *apiAT6-2^∆201-664^* |
| TGGT1_249580 | *Tg*ApiAT6-3 | TATi/Tomato | 1 bp insertion | 509 | *apiAT6-3^∆170-684^* |
| TGGT1_263230 | *Tg*ApiAT7-1 | TATi/Tomato | 1 bp insertion | 601 | *apiAT7-1^∆201-891^* |
| TGGT1_263260 | *Tg*ApiAT7-2 | TATi/Tomato | 983 bp insertion | 2810 | *apiAT7-2^∆937-1027^* |
