# Supplementary material for: The tyrosine transporter of Toxoplasma gondii is a member of the newly defined apicomplexan amino acid transporter (ApiAT) family
Source: PLoS Pathog. 2019 Feb 11;15(2):e1007577. doi: 10.1371/journal.ppat.1007577 (PMC6386423; doi:10.1371/journal.ppat.1007577)
Supplement: S3 Table — (DOCX) [file ppat.1007577.s013.docx]

**S3 Table.** The amino acid compositions of Roswell Park Memorial Institute 1640 medium (RPMI), Dulbecco’s Modified Eagle’s Medium (DMEM) and Minimal Amino Acid Medium (MAAM) used in this study.

| Amino acid | Concentration (µM) | | |
| --- | --- | --- | --- |
|  | RPMI | DMEM | MAAM |
| Alanine | 0 | 0 | 0 |
| Asparagine | 379 | 0 | 0 |
| Aspartic acid | 150 | 0 | 0 |
| Glutamic acid | 136 | 0 | 0 |
| Hydroxyproline | 153 | 0 | 0 |
| Proline | 174 | 0 | 0 |
| Glycine | 133 | 400 | 0 |
| Serine | 286 | 400 | 0 |
| Tryptophan | 24.5 | 78 | 7.8 |
| Phenylalanine | 90.9 | 400 | 15.63 |
| Isoleucine | 382 | 800 | 31.25 |
| Leucine | 382 | 800 | 31.25 |
| Methionine | 101 | 201 | 31.25 |
| Threonine | 168 | 800 | 31.25 |
| Histidine | 96.8 | 200 | 62.5 |
| Tyrosine | 111 | 423 | 62.5 |
| Lysine | 219 | 800 | 100 |
| Cystine | 208 | 200 | 125 |
| Valine | 171 | 800 | 250 |
| Arginine | 1150 | 400 | 287 |
| Glutamine | 2050 | 4000 | 500 |
