# Supplementary material for: The tyrosine transporter of Toxoplasma gondii is a member of the newly defined apicomplexan amino acid transporter (ApiAT) family
Source: PLoS Pathog. 2019 Feb 11;15(2):e1007577. doi: 10.1371/journal.ppat.1007577 (PMC6386423; doi:10.1371/journal.ppat.1007577)
Supplement: S4 Table — (DOCX) [file ppat.1007577.s014.docx]

**S4 Table. Composition and concentrations of metabolite mixes used in S7B Fig.**

| **Heterogeneous Mix name** | **Composition** | **[final substrate] (mM)** |
| --- | --- | --- |
| L-amino acid mix 1 (L-AA 1) | L-aspartate | 5 |
|  | L-proline |  |
|  | L-leucine |  |
|  | glycine |  |
| L-amino acid mix 2 (L-AA 2) | L-glutamate | 5 |
|  | L-valine |  |
|  | L-methionine |  |
|  | L-arginine |  |
|  | L-serine |  |
|  | L-threonine |  |
|  | L-cysteine |  |
| L-amino acid mix 3 (L-AA 3) | L-Isoleucine | 5 |
|  | L-histidine |  |
|  | L-asparagine |  |
|  | L-alanine |  |
|  | L-glutamine |  |
|  | L-phenylalanine |  |
|  | L-tryptophan | 2 |
| Amino acid derivatives 1  (AA derivatives 1) | taurine | 5 |
|  | betaine |  |
|  | dopamine |  |
|  | GABA |  |
|  | 4-hydroxy-L-Proline |  |
|  | β-alanine |  |
|  | L-theanine |  |
|  | serotonin |  |
| Amino acid derivatives 2  (AA derivatives 2) | D-leucine | 2.5 |
|  | D-tryptophan | 0.1 |
|  | creatine | 0.25 |
| Amino acid derivatives 3  (AA derivatives 3) | agmatine | 5 |
|  | L-citrulline |  |
|  | L-ornithine |  |
|  | putrescine |  |
|  | spermidine |  |
|  | urea |  |
| D-amino acids (D-AA) | D-alanine | 5 |
|  | D-aspartate |  |
|  | D-serine |  |
|  | D-methionine |  |
|  | D-glutamine |  |
| Nucleosides | adenosine | 0.5 |
|  | guanosine |  |
|  | thymidine |  |
|  | uridine |  |
|  | cytidine |  |
| Nitrogenous bases  (nitrogen bases) | thymine | 0.5 |
|  | adenine |  |
|  | guanine |  |
|  | uracil |  |
|  | hypoxanthine |  |
| Sugars | D-glucose | 5 |
|  | D-galactose |  |
|  | D-mannose |  |
|  | D-sorbitol |  |
|  | D-trehalose |  |
|  | myo-inositol |  |
|  | D-fructose |  |
|  | sucrose |  |
