# Supplementary material for: The tyrosine transporter of Toxoplasma gondii is a member of the newly defined apicomplexan amino acid transporter (ApiAT) family
Source: PLoS Pathog. 2019 Feb 11;15(2):e1007577. doi: 10.1371/journal.ppat.1007577 (PMC6386423; doi:10.1371/journal.ppat.1007577)
Supplement: S5 Table — (DOCX) [file ppat.1007577.s015.docx]

**S5 Table.** Forward primers used to generate CRISPR/Cas9 vectors targeting *Tg*ApiAT genes for gene disruption.

| Target gene | Primer sequence |
| --- | --- |
| *Tg*ApiAT1 | 5’-CGTCATCTGTTTATTTCGGAGTTTTAGAGCTAGAAATAGCAAG |
| *Tg*ApiAT2 | 5’-AGTACCCGGACTGTCCAGGAGTTTTAGAGCTAGAAATAGCAAG |
| *Tg*ApiAT3-1 | 5’-ACCGCCACGTGCATCTTCTTGTTTTAGAGCTAGAAATAGCAAG |
| *Tg*ApiAT3-2 | 5’- GGGGGTCGGCTGGAGGCGGGGTTTTAGAGCTAGAAATAGCAAG |
| *Tg*ApiAT3-3 | 5’-AGGAAAGCAAGTTGCAGAGTGTTTTAGAGCTAGAAATAGCAAG |
| TgApiAT5-1 | 5’-GTTGATTGCTGTCTCCACGGGTTTTAGAGCTAGAAATAGCAAG |
| *Tg*ApiAT5-2 | 5’-CCTGCGGATCGCAAACCCAGGTTTTAGAGCTAGAAATAGCAAG |
| TgApiAT5-3 | 5’-GTTTCTCCTCGGAAAGCTTCGTTTTAGAGCTAGAAATAGCAAG |
| *Tg*ApiAT5-4 | 5’-ACTTGCCACTGACTGGCGACGTTTTAGAGCTAGAAATAGCAAG |
| *Tg*ApiAT5-5 | 5’-AAAGGCTGTCCAGTTCCAGTGTTTTAGAGCTAGAAATAGCAAG |
| *Tg*ApiAT5-6 | 5’-GCTCGCGCAAGAGCGGTTTCGTTTTAGAGCTAGAAATAGCAAG |
| *Tg*ApiAT6-1 | 5’-GCCGTAGAAGACCGCTCCCGGTTTTAGAGCTAGAAATAGCAAG |
| *Tg*ApiAT6-2 | 5’-CGCAGAGCTCGCCGAAGAGGGTTTTAGAGCTAGAAATAGCAAG |
| *Tg*ApiAT6-3 | 5’-AGCACCATGTGCGCATTCGCGTTTTAGAGCTAGAAATAGCAAG |
| *Tg*ApiAT7-1 | 5’-CAAATCTGGGGCGTTCAGCTGTTTTAGAGCTAGAAATAGCAAG |
| *Tg*ApiAT7-2 | 5’-GCGATCCCTTACCCGTTGCGGTTTTAGAGCTAGAAATAGCAAG |
