# Supplementary material for: The tyrosine transporter of Toxoplasma gondii is a member of the newly defined apicomplexan amino acid transporter (ApiAT) family
Source: PLoS Pathog. 2019 Feb 11;15(2):e1007577. doi: 10.1371/journal.ppat.1007577 (PMC6386423; doi:10.1371/journal.ppat.1007577)
Supplement: S6 Table — (DOCX) [file ppat.1007577.s016.docx]

**S6 Table.** Forward primers used to generate CRISPR/Cas9 vectors targeting *Tg*ApiAT genes for 3’ replacement with an HA epitope tag.

| *Target gene* | Primer sequence |
| --- | --- |
| *Tg*ApiAT3-1 | 5’-ACAGCTGAGAAGAGTTTTGTGTTTTAGAGCTAGAAATAGCAAG |
| *Tg*ApiAT5-1 | 5’-ACGTTTTTCTCAGACGGAATGTTTTAGAGCTAGAAATAGCAAG |
| *Tg*ApiAT5-2 | 5’-GATTTTTTACAGAGCAAGGAGTTTTAGAGCTAGAAATAGCAAG |
| *Tg*ApiAT5-4 | 5’-CTTCATCACTCCCACGTCCCGTTTTAGAGCTAGAAATAGCAAG |
| *Tg*ApiAT5-5 | 5’-AAAACAAAGCATTCAGCCGGGTTTTAGAGCTAGAAATAGCAAG |
| *Tg*ApiAT5-6 | 5’-ATTCAAAGTCACTCGTGCAAGTTTTAGAGCTAGAAATAGCAAG |
| *Tg*ApiAT6-3 | 5’-CACCAGACGGCTGAGAGCCTGTTTTAGAGCTAGAAATAGCAAG |
| *Tg*ApiAT7-2 | 5’-GAGTGAGACGGCGCAGAAAGGTTTTAGAGCTAGAAATAGCAAG |
