# Supplementary material for: The tyrosine transporter of Toxoplasma gondii is a member of the newly defined apicomplexan amino acid transporter (ApiAT) family
Source: PLoS Pathog. 2019 Feb 11;15(2):e1007577. doi: 10.1371/journal.ppat.1007577 (PMC6386423; doi:10.1371/journal.ppat.1007577)
Supplement: S7 Table — (DOCX) [file ppat.1007577.s017.docx]

**S7 Table.** Primers used to amplify 3x HA tags containing 50 bp flanking sequences to the target gene, as well as sequence of the gBlock oligonucleotide encoding the 3x HA tag used as template.

| Target gene | Oligo name | Primer sequence (5’ to 3’) |
| --- | --- | --- |
| *Tg*ApiAT3-1 | *Tg*ApiAT3-1 forward | GTGGCGGGGCTCTCGCAGCGGACCCGGCTGTGGCTGTCACAGTGCACAGCGGTGGAGGTAGCGGTGGTGGAAG |
|  | *Tg*ApiAT3-1 reverse | ACCCCACATCGGAGCTGGCGCGTGGTTGATTTAAGGAGACAAAACTCTTCGCTTCTGTGGGCGGTTATCAGG |
| *Tg*ApiAT5-1 | *Tg*ApiAT5-1 forward | AGCCGATCAAGAGCCTCTCTGCAACCTACGGCCTGGCGTCAGATTCCGTCGGTGGAGGTAGCGGTGGTGGAAG |
|  | *Tg*ApiAT5-1 reverse | TCCTCACCACATTTTTCGGTTCGTCTCCGAGACCGAGGAGAACGTTTTTCGCTTCTGTGGGCGGTTATCAGG |
| *Tg*ApiAT5-2 | *Tg*ApiAT5-2 forward | TGAAAAGACAAGCGGAAACCGCGACGCCGTGCACTGCTGTCCTTGCTCTGGGTGGAGGTAGCGGTGGTGGAAG |
|  | *Tg*ApiAT5-2 reverse | CGGCGTGCGTTGGTGTGCGTCGGTGCTGCGATGGTTCTTCCAAAGATTTTGCTTCTGTGGGCGGTTATCAGG |
| *Tg*ApiAT5-4 | *Tg*ApiAT5-4 forward | CAACAAAAACCAAGGTCGCGCGTTTGCAGCATCGACTCTCCAACGCCAACGGTGGAGGTAGCGGTGGTGGAAG |
|  | *Tg*ApiAT5-4 reverse | AAGTGGTAGCTCTTCATCACTCCCACGTCCCTATCTGTTCTGCGCGCGCGGCTTCTGTGGGCGGTTATCAGG |
| *Tg*ApiAT5-5 | *Tg*ApiAT5-5 forward | AACTCCTTGCGGAGACCGAAGTCGCCTTCCAATCTGATATGGCAACCGGCGGTGGAGGTAGCGGTGGTGGAAG |
|  | *Tg*ApiAT5-5 reverse | CGCCGCAACGGCTACAAGAAGTGTATGAAACTCTTTTCAAAACAAAGCATGCTTCTGTGGGCGGTTATCAGG |
| *Tg*ApiAT5-6 | *Tg*ApiAT5-6 forward | GAGCAACGACGGTACTCTCAAGCATTCATTCAGAGGACGCGTTGCACGAGGGTGGAGGTAGCGGTGGTGGAAG |
|  | *Tg*ApiAT5-6 reverse | GCAGATTTATTATCGAAACCGCAAGCTTCATCTTAACATCAAATTCAAAGGCTTCTGTGGGCGGTTATCAGG |
| *Tg*ApiAT6-3 | *Tg*ApiAT6-3 forward | GAGAAGGGCGAGCGGTAGAGTGTAGTGGAGACGGCACAGCACCAGACGGCGGTGGAGGTAGCGGTGGTGGAAG |
|  | *Tg*ApiAT6-3 reverse | CAAAGGTGGAACTGTGGAGTCGTCCCTGTTCACGTCTTCGTAGTAGGCTCGCTTCTGTGGGCGGTTATCAGG |
| *Tg*ApiAT7-2 | *Tg*ApiAT7-2 forward | TACAGCCCGCCGAAGAAAAGGAGCACGGCAAAGACGGAAGCGGAATGGAGGGTGGAGGTAGCGGTGGTGGAAG |
|  | *Tg*ApiAT7-2 reverse | AAGGACCCGACTTTCTTGCGCAGTTTCCTGTGATAGCTTTCTGCGCCGTCGCTTCTGTGGGCGGTTATCAGG |
| N/A | 3xHA gBlock template | GGTGGAGGTAGCGGTGGTGGAAGTTACCCGTACGACGTCCCGGACTACGCTGGCTATCCCTATGATGTGCCCGATTATGCGTATCCTTACGATGTTCCAGATTATGCCTGATAACCGCCCACAGAAGC |
