# Supplementary material for: The tyrosine transporter of Toxoplasma gondii is a member of the newly defined apicomplexan amino acid transporter (ApiAT) family
Source: PLoS Pathog. 2019 Feb 11;15(2):e1007577. doi: 10.1371/journal.ppat.1007577 (PMC6386423; doi:10.1371/journal.ppat.1007577)
Supplement: S8 Table — Flanking sequences were ligated into the pgCH vector (unless indicated) and linearized with the indicated restriction enzyme. (DOCX) [file ppat.1007577.s018.docx]

**S8 Table.** Primers used to amplify 3’ flanks from target *Tg*ApiAT genes. Flanking sequences were ligated into the pgCH vector (unless indicated) and linearized with the indicated restriction enzyme.

| Target gene | Restriction enzyme | Oligo name | Primer sequence (5’ to 3’) |
| --- | --- | --- | --- |
| *Tg*ApiAT2 | *Nsi*I | *Tg*ApiAT2 forward | CCCTAAGATCTGCATGCTCAGCG |
|  |  | *Tg*ApiAT2 reverse | GATCCCTAGGCACAGCGACCTCTGGACTCGGT |
| *Tg*ApiAT3-2 | *Nsi*I | *Tg*ApiAT3-2 forward | GATCAGATCTGTACACATGCGTGTGGCTCA |
|  |  | *Tg*ApiAT3-2 reverse | GATCCCTAGGTGATCTATCTATCGCAACGCAAACG |
| *Tg*ApiAT3-3  (ligated into pLIC-HA_3_-DHFR) | *Nco*I | *Tg*ApiAT3-3 forward | GATCTTAATTAACACTTCGCGGACACCTCGCC |
|  |  | *Tg*ApiAT3-3 reverse | GATCCCTAGGGGCCATCTCGACAGCATGCGCC |
| *Tg*ApiAT5-3 | *Bgl*II | *Tg*ApiAT5-3 forward | GATCGGATCCTGGAGCCAGCCTCTCCAGCTTCG |
|  |  | *Tg*ApiAT5-3 reverse | GATCCCTAGGCAGCACCTTCGGGACTTTTCTCTTC |
| *Tg*ApiAT6-1 | *Bst*Z17I | *Tg*ApiAT6-1 forward | GACTAGATCTTATCTGCGACGAACAGGACG |
|  |  | *Tg*ApiAT6-1 reverse | GTACCCTAGGAGCGGAGTCTTGCGG |
| *Tg*ApiAT6-2 | *Sph*I | *Tg*ApiAT6-2 forward | GATCGGATCCTCCTCCCCACCATGTGCGTC |
|  |  | *Tg*ApiAT6-2 reverse | GTCAACTAGTGACTTCACAAACAGGCTCGAAAGG |
| *Tg*ApiAT7-1 | *Sph*I | *Tg*ApiAT7-1 forward | GTCAGGATCCGTATCCTCGAAAAGCTCAAAATCC |
|  |  | *Tg*ApiAT7-1 reverse | GATCCCTAGGCGACTTGTCTTGAAAGAGCGCC |
